# Reduction of Rare‐Earth Stannole Sandwich Complexes to Tin‐Based Radical Ligands and Tin–Tin Bonds

**DOI:** 10.1002/anie.202516323

**Published:** 2025-09-14

**Authors:** Siddhartha De, Arpan Mondal, Jinkui Tang, Richard A. Layfield

**Affiliations:** ^1^ Department of Chemistry School of Life Sciences University of Sussex Brighton BN1 9RH UK; ^2^ State Key Laboratory of Rare Earth Resource Utilization Changchun Institute of Applied Chemistry Chinese Academy of Sciences Changchun P.R. China

**Keywords:** Lanthanides, Magnetic properties, Organometallics, Radical ligand, Tin

## Abstract

f‐Element organometallic chemistry is dominated by cyclopentadienyl ligands. In contrast, isoelectronic metallole ligands with the general formula [EC_4_R_4_]^2−^, where E is a heavier group 14 element, are rare in the f‐block, particularly stannole ligands. Here, we describe the synthesis of the dimetallic stannole complexes [(η^5^‐Cp^Sn^)M(η^5^‐Cp^ttt^)]_2_ (**1**_M_ ; M = Y, Gd, Dy; Cp^Sn^ = [SnC_4_‐2,5‐(SiMe_3_)_2_–3,4‐Me_2_]^2−^, Cp^ttt^ = [1,2,4‐C_5_ *^t^* Bu_3_H_2_]^−^), which form by virtue of Sn→M dative bonds. One‐electron reduction of **1_M_** with KC_8_/2.2.2‐cryptand produces the mono‐anionic complexes [{(η^5^‐Cp^Sn^)M(η^5^‐Cp^ttt^)}_2_]^−^ (**2_M_**), and two‐electron reduction gives di‐anionic [{(η^5^‐Cp^Sn^)M(η^5^‐Cp^ttt^)}_2_]^2−^ (**3_M_**) as [K(2.2.2‐crypt)]^+^ salts. Studies of the stannole complexes using crystallography, UV/vis and EPR spectroscopy, magnetometry and computational methods reveal that the reduction steps generate tin–tin bonds through population of a delocalized molecular orbital that spans the {M_2_Sn_2_} rings, with attendant dearomatization of the stannole rings. Complexes **2_M_** are the first tin‐radical ligands bound to rare earth elements. Spin density calculations of **2_Y_** and **2_Gd_** reveal significant build‐up of unpaired spin on the tin atoms, with magnetic measurements on **2_Gd_** yielding an unprecedentedly large tin–gadolinium exchange coupling constant of −112 cm^−1^ (−2*J* formalism).

## Graphical abstract

One‐electron reduction of dimetallic rare‐earth stannole complexes with KC_8_ produces a di‐tin radical ligand that shows strong coupling between tin and yttrium, gadolinium or dysprosium. Two‐electron reduction of the stannole complexes further populates a delocalized {M_2_Sn_2_} molecular orbital, enhancing the tin–tin bonding and simultaneously dearomatizing the stannole rings.

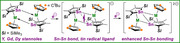

## Introduction

The synthesis and isolation of persistent main group radicals has emerged as a topic of considerable interest in recent years, particularly in the case of heavier p‐block metals and metalloids.^[^ ^1^, ^2^ ^]^ Impressive progress has been made by stabilizing neutral, anionic, and cationic radicals of heavier main group elements using kinetic (steric) and/or electronic (orbital‐based) approaches, notably with carbene ligands such as cyclic alkyl–amino carbenes bound to the formal radical centre.^[^ ^3^ ^]^ Whilst motivation for further research into heavier main group radicals stems from their reactivity, another application of these species lies in molecular magnetism, where they can be deployed as ligands to interact with the unpaired electrons of, for example, lanthanides. Indeed, the use of radical ligands based on light main group elements (carbon, nitrogen, and oxygen) in lanthanide single‐molecule magnets (SMMs) has proven to be an effective strategy for targeting “hard” magnet behaviour.^[^ ^4^, ^5^ ^]^ In some radical‐bridged multimetallic SMMs, strong lanthanide‐radical exchange coupling mitigates the low‐temperature rapid relaxation of magnetization that would otherwise occur,^[^ ^6^, ^7^, ^8^, ^9^, ^10^, ^11^, ^12^, ^13^, ^14^, ^15^ ^]^ forming the basis of proposals to use such molecular magnets in quantum technologies.^[^ ^16^ ^]^

In contrast to radicals composed of lighter main group elements, the use of heavier p‐block radicals in lanthanide molecular magnets is rare. To the best of our knowledge, this chemistry is limited to a few lanthanide metallocenes bound to a phosphorus‐centred heterocyclic radical,^[^ ^17^ ^]^ dimetallic lanthanide metallocenes containing the exotic [Bi_2_]^3−^ radical,^[^ ^18^ ^]^ and samarocene and ytterbocene complexes of an acyclic sulfur–nitrogen radical.^[^ ^19^ ^]^ Beyond magnetism, the diffuse orbitals of heavier p‐block elements also introduce the possibility of greater metal‐ligand covalency, a topic of fundamental importance in lanthanide chemistry.^[^ ^20^ ^]^

Our interests in lanthanide complexes of heavy p‐block radicals was recently piqued by the discovery that one‐electron reduction of the dimetallic germole‐ligated sandwich complexes [(η^5^‐Cp^Ge^)M(η^5^‐Cp^ttt^)]_2_, where Cp^Ge^ is the germole dianion [GeC_4_‐2,5‐(SiMe_3_)_2_–3,4‐Me_2_]^2−^, Cp^ttt^ is [1,2,4‐C_5_ *^t^* Bu_3_H_2_]^−^, and M is yttrium, gadolinium, or dysprosium, produces the corresponding mono‐anionic complexes [{(η^5^‐Cp^Ge^)M(η^5^‐Cp^ttt^)}_2_]^−^.^[^ ^21^ ^]^ Analysis of these germanium radical‐bridged species revealed that the unpaired electron occupies a highly delocalized molecular orbital (MO) spanning the {M_2_Ge_2_} core, including an unexpected germanium–germanium bond. Magnetic susceptibility measurements of [{(η^5^‐Cp^Ge^)Gd(η^5^‐Cp^ttt^)}_2_]^−^ yielded a gadolinium‐radical exchange coupling constant of −95 cm^−1^ (−2*J* formalism), i.e., roughly two orders of magnitude larger than typically found in gadolinium complexes of light main group radical ligands.^[^ ^4^, ^5^ ^]^

Having observed that further reduction of the radical‐bridged mono‐anion [{(η^5^‐Cp^Ge^)M(η^5^‐Cp^ttt^)}_2_]^−^ to the di‐anion [{(η^5^‐Cp^Ge^)M(η^5^‐Cp^ttt^)}_2_]^2−^ enhances the M─Ge and Ge─Ge bonding, we were interested to explore the analogous stannole chemistry. Whilst the use of group 14 metallole ligands in f‐element organometallic chemistry is an emerging area in general, the use of stannole ligands is currently limited to the erbium sandwich SMM [(η^5^‐Cp^Sn^)Er(η^8^‐COT)]^−^, where Cp^Sn^ is [SnC_4_‐2,5‐(SiMe_3_)_2_–3,4‐Me_2_]^2−^ and COT is cyclo‐octatetraenyl.^[^ ^22^ ^]^ The greater size of tin relative to germanium and the attendant weaker bonding involving the heavier group 14 metal could mean that complexes of the type [{(η^5^‐Cp^Sn^)M(η^5^‐Cp^ttt^)}_2_]*^n^* ^−^ (*n* = 0, 1, 2) show divergent structural and bonding properties, as well as providing a way of tuning the magnetism of paramagnetic versions via the group 14 element. The synthesis of mono‐anionic [{(η^5^‐Cp^Sn^)M(η^5^‐Cp^ttt^)}_2_]^−^ would also provide the first example of a tin‐based radical ligand in lanthanide chemistry.

## Results

### Synthesis and Structural Studies

**Scheme 1:**
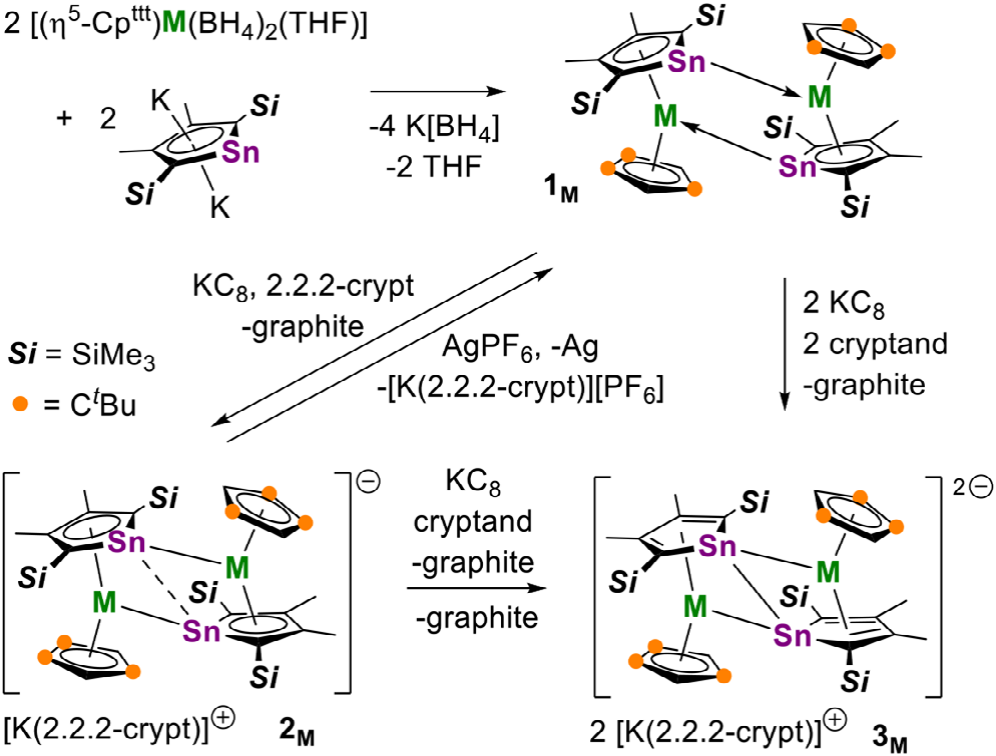
Synthesis of **1** **_M_**, [K(2.2.2‐crypt)][**2** **_M_**] and [K(2.2.2‐crypt)]_2_[**3** **_M_**] (M = Y, Gd, Dy).

The synthesis of the dimetallic stannole complexes [(η^5^‐Cp^Sn^)M(η^5^‐Cp^ttt^)]_2_ (**1_M_**) with M = Y, Gd, Dy was accomplished according to Scheme 1. The choice of yttrium was determined by its diamagnetic nature, facilitating characterization by NMR spectroscopy. Gadolinium was chosen to explore the magnetic exchange coupling using an isotropic 4f^7^ species, and dysprosium was selected for the potential SMM properties. The isolated yields of crystalline **1_M_** were typically in the region of 50%–55%. Subsequently, reduction of **1_M_** using one stoichiometric equivalent of KC_8_/2.2.2‐cryptand gave the 1:1 salts [K(2.2.2‐crypt)][{(η^5^‐Cp^Sn^)M(η^5^‐Cp^ttt^)}_2_] ([K(2.2.2‐crypt)][**2_M_**]) in isolated yields of 60%–65%. Adding two equivalents of KC_8_/2.2.2‐cryptand to **1_M_** produced the 2:1 salts [K(2.2.2‐crypt)]_2_[{(η^5^‐Cp^Sn^)M(η^5^‐Cp^ttt^)}_2_] ([K(2.2.2‐crypt)]_2_[**3_M_**]) in 50%–60% yields.

**Figure 1:**
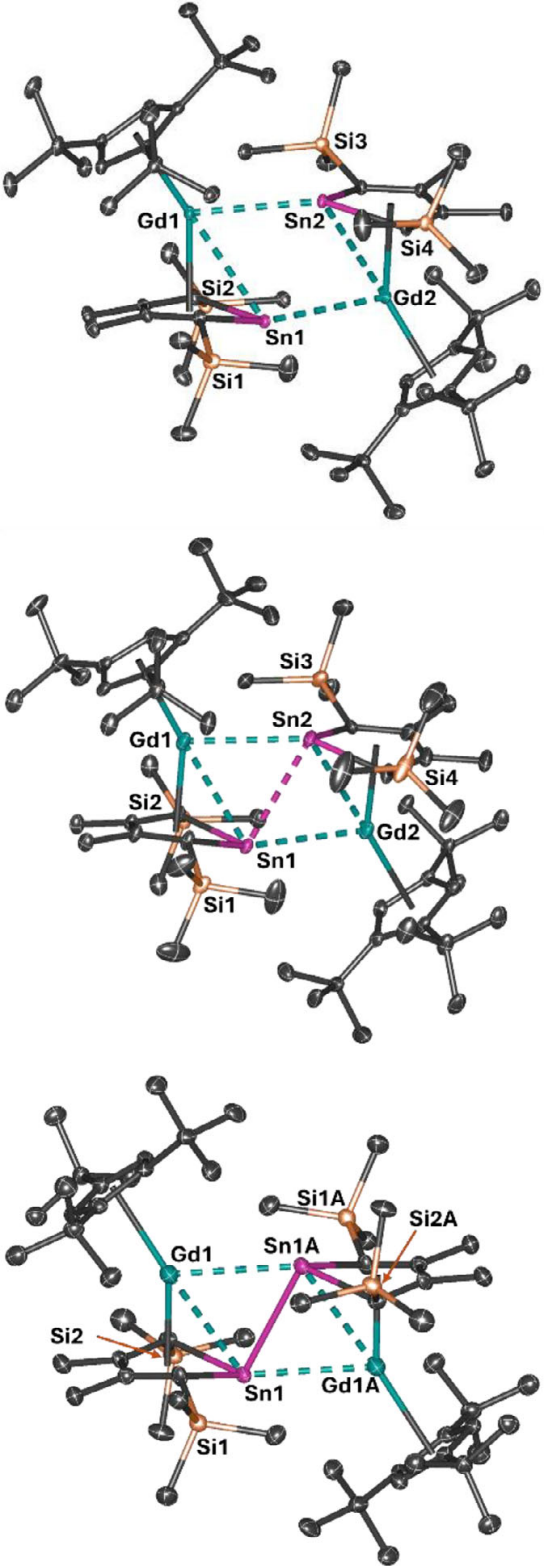
Molecular structures of **1_Gd_** (top), the mono‐anion **2_Gd_** (centre), and the di‐anion **3_Gd_** (bottom). Thermal ellipsoids are at the 50% probability level. For clarity, hydrogens atoms are not shown.

The molecular structures of all nine compounds were determined by X‐ray crystallography (Tables S1–S6, Figure 1, S1–S3).^[^ ^23^ ^]^ The three **1** **_M_** compounds are isostructural, consistent with their similar FTIR spectra, as are the singly reduced compounds [K(2.2.2‐crypt)][**2** **_M_**] and doubly reduced [K(2.2.2‐crypt)]_2_[**3** **_M_**] (Figures S4–S6). The structures of the gadolinium versions are discussed in detail, with details of the yttrium and dysprosium analogues provided in the Supporting Information.

In the series **1_Gd_**, **2_Gd_**, and **3_Gd_**, stepwise reduction results in a pronounced decrease of the Gd–(Cp^Sn^)_cent_ distances from 2.3821(11)/2.3911(11) Å in **1_Gd_** to 2.320(3)/2.300(3) Å in **2_Gd_** and then to 2.253(4) Å in **3_Gd_** (cent is the centroid of the ligand). Simultaneously, the Gd–(Cp^ttt^)_cent_ distances increase in the order 2.4273(13)/2.4247(15) Å in **1_Gd_** to 2.485(3)/2.465(3) Å in **2_Gd_**, and 2.514(4) Å in **3_Gd_**. Substantial shortening of the Gd–Sn distances to the η^5^‐Cp^Sn^ ligands occurs in the order 3.1826(6)/3.1882(5) Å, 3.1480(7)/3.1232(8) Å, and 3.1028 (7) Å for **1_Gd_**, **2_Gd_**, and **3_Gd_**, respectively. In contrast, only a slight variation occurs in the dative Gd–Sn interactions to the η^1^‐Cp^Sn^ ligands, with distances of 3.1849(6)/3.1676(5) Å in **1_Gd_**, 3.1649(6)/3.1760(8) Å in **2_Gd_**, and 3.1799(7) Å in **3_Gd_**. The stepwise reductions are also accompanied by a marked decrease in the tin–tin distance, i.e., from 3.2154(4) Å in **1_Gd_** to 3.1780(6) Å in **2_Gd_** and 3.0989(13) Å in **3_Gd_**, whereas a slight lengthening in the Gd⋅⋅⋅Gd separation occurs from 5.3506(7) Å in **1_Gd_** to 5.3672(7) Å in **2_Gd_**, followed by a much larger increase to 5.4658(9) Å in **3_Gd_**. The increase in separation between the gadolinium ions with each reduction, which is also found in the yttrium and dysprosium analogues (Tables S4, S6), presumably occurs to accommodate a progressively stronger transannular tin–tin interaction.

**Scheme 2:**
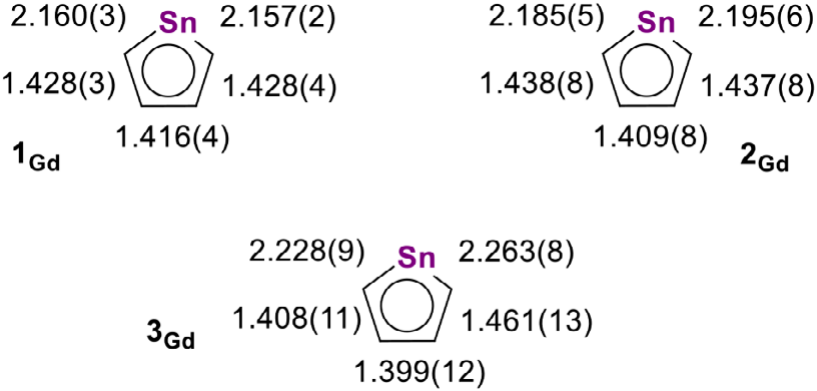
Bond lengths (Å) within the stannole ligands of **1_Gd_**, **2_Gd_**, and **3_Gd_** (shown for one of two unique stannole ligands in **1_Gd_** and **2_Gd_**).

Another distinct variation in the structures of the gadolinium complexes relates to the distribution of Sn–C and C–C distances within the stannole rings, as shown in Scheme 2. A significant increase in the Sn─C bond lengths from **1_Gd_** to **3_Gd_** occurs along with short–long–short alternating C─C bond lengths. In addition, puckering of the SnC_4_ ring is observed, reflected in the C1‐Sn‐C3‐C4 torsional angles of 12.86(17)/11.97(17)°, 14.9(4)/15.7(5)°, and 22.4(7)° in **1_Gd_**, **2_Gd_**, and **3_Gd_**, respectively. These structural data could indicate a loss in the aromatic character of the stannole ring upon reduction to give **3_Gd_**, with similar patterns observed for the yttrium and dysprosium versions (Tables S4, S6).

Turning to solution‐phase studies of the diamagnetic yttrium complex **1_Y_** by NMR spectroscopy, a 1:1 doublet occurs at *δ* = 529.6 ppm in the ^119^Sn{^1^H} NMR spectrum in toluene‐D_8_, presumably due to coupling between ^89^Y (*I* = 1/2, 100% abundance) and ^119^Sn via the η^1^–Cp^Sn^ interaction, with ^1^ *J* = 444.6 Hz (Figure S7). This observation is consistent with retention of the dimeric structure in solution. The ^1^H NMR spectrum of **1_Y_** consists of resonances at *δ* = 0.06 ppm for the SiMe_3_ groups, 1.28 and 1.49 ppm for the *tert*‐butyl groups, 2.70 ppm for the stannole methyl substituents, and at 6.42 ppm for the cyclopentadienyl protons (Figure S8). The ^1^H/^29^Si HMBC spectrum of **1_Y_** allows the ^29^Si chemical shift to be identified at *δ* = −8.42 ppm (Figure S9). In contrast to **1_Y_**, the ^1^H NMR spectrum of paramagnetic [K(2.2.2‐crypt)][**2_Y_**] in THF‐D_8_ only shows resonances due to the cryptand ligand, which are partially obscured by residual proton solvent resonances (Figure S11). The ^1^H NMR spectrum of [K(2.2.2‐crypt)]_2_[**3_Y_**] in THF‐D_8_ features resonances at −0.25 ppm for the SiMe_3_ groups, 1.02 and 1.54 ppm for the *tert*‐butyl groups, 2.58, 3.54, 3.61 ppm for the cryptand ligand, and 5.70 ppm for the cyclopentadienyl protons (Figure S12). A single resonance was observed for the trimethylsilyl groups in the ^29^Si/^1^H HMBC NMR spectrum at *δ* = −11.41 ppm (Figure S13), but no signal was observed in the ^119^Sn{^1^H} NMR spectrum (Figure S14).

Following characterization of the solution‐phase structures of the three yttrium–stannole complexes, ^1^H NMR spectroscopy was used to determine if [K(2.2.2‐crypt)][**2_Y_**] could be converted into **1_Y_** and [K(2.2.2‐crypt)]_2_[**3_Y_**] by means of simple oxidation and reduction reactions, respectively. Thus, adding one equivalent of AgPF_6_ to [K(2.2.2‐crypt)][**2_Y_**] produced **1_Y_** (Figure S15), and adding one equivalent of KC_8_/2.2.2‐crypt to [K(2.2.2‐crypt)][**2_Y_**] generates [K(2.2.2‐crypt)]_2_[**3_Y_**] (Figure S16), with both reactions proceeding cleanly and essentially quantitatively in THF‐D_8_.

### Bonding and Electronic Structure

To gain insight into the bonding in the yttrium and gadolinium stannole complexes, density functional theory (DFT) calculations were performed using the coordinates obtained from the crystallographic studies. The ORCA 6.0.0 software package was used for these calculations,^[^ ^24^, ^25^ ^]^ with full details provided in the Supporting Information.

**Figure 2:**
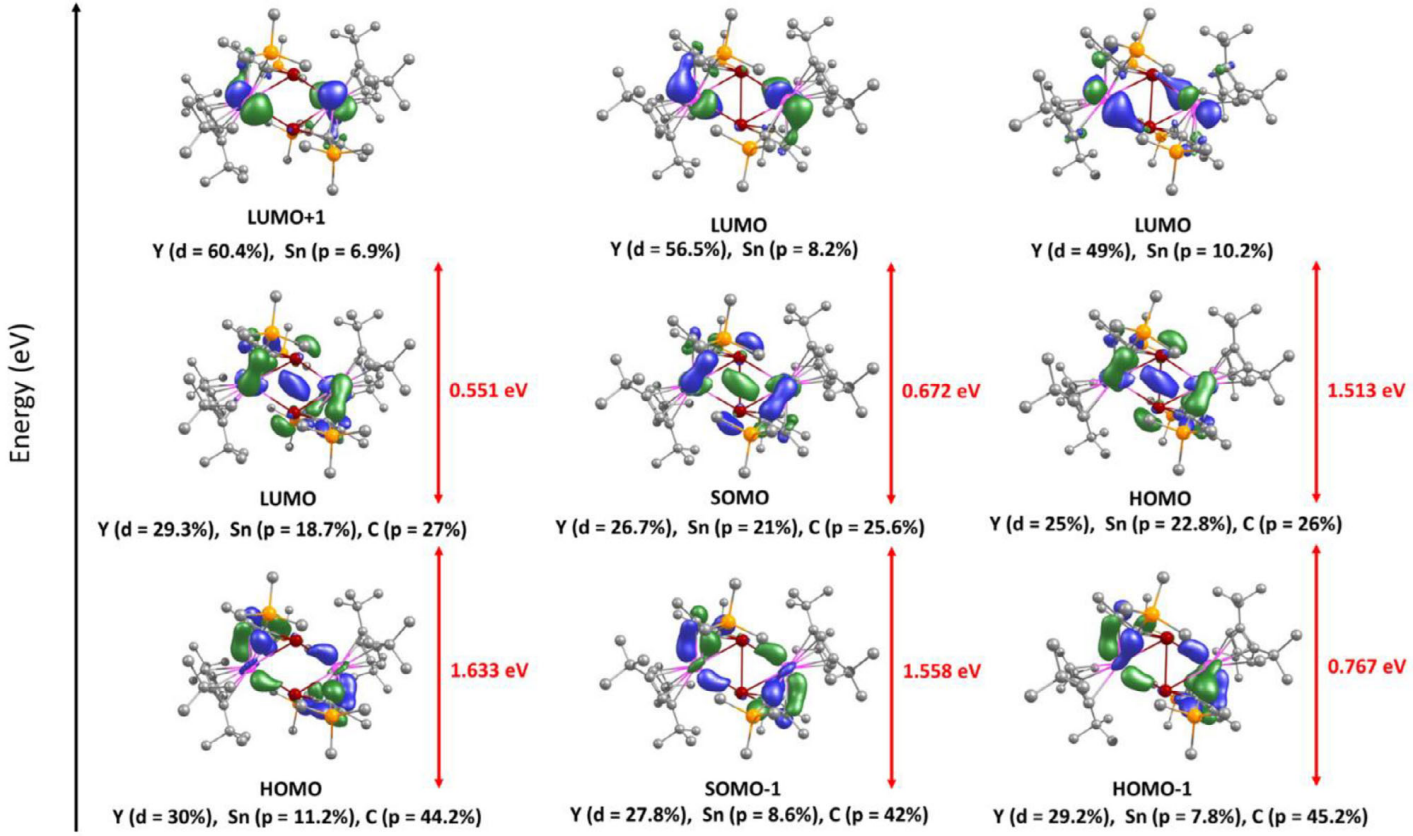
Frontier molecular orbitals for **1_Y_** (left), the mono‐anion **2_Y_** (centre), and the di‐anion **3_Y_** (right). Isosurface value = 0.04 a.u.

**Figure 3:**
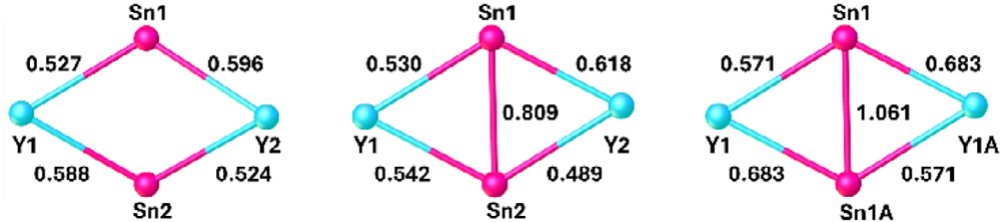
Wiberg bond index values for **1_Y_** (left), **2_Y_** (centre), and **3_Y_** (right).

The overall appearance and composition of the frontier MOs in compounds **1** **_M_**, the singly reduced complexes **2** **_M_**, and doubly reduced **3** **_M_** (M = Y, Gd) are similar (Figures 2, S17). In the case of **1** **_M_**, the HOMOs (highest‐occupied MOs) and LUMOs (lowest‐unoccupied MOs) consist of appreciable contributions from the yttrium 4d (30%) and gadolinium 5d (30%) orbitals in addition to the tin 5p orbitals (11% and 10%, respectively). Notably, the LUMOs in **1** **_M_** feature overlap between the tin‐based orbitals, which effectively become the SOMOs (singly occupied MOs) following one‐electron reduction to give **2** **_M_**. The SOMO for **2_Y_** features 27% yttrium 4d character and a tin 5p contribution of 21%, and for **2_Gd_** the gadolinium 5d and tin 5p orbitals contribute 33% and 18%, respectively. Adding a second electron to the SOMO in **2_Y_** and **2_Gd_** to give **3_Y_** and **3_Gd_** results in HOMOs with 25% 4d and 23% 5p character for the yttrium complex, and 31% 5d and 19% 5p character for the gadolinium complex. The Wiberg bond index (WBI) for the tin–tin interaction in **2_Y_** is 0.81, increasing to 1.06 in **3_Y_** (Figure 3). The WBIs for the yttrium–tin interactions were calculated for all three compounds and found to increase slightly with each reduction step. Overall, the pattern of WBIs for the yttrium–stannole complexes are consistent with the variation in the bond lengths within the {Y_2_Sn_2_} cores.

Noting the structural changes that occur within the stannole rings with each reduction step, nucleus‐independent chemical shift (NICS) calculations were carried out on diamagnetic **1_Y_** and **3_Y_** to explore the impact on the aromaticity of these formally 6π‐electron systems.^[^ ^26^ ^]^ In **1_Y_**, the aromatic character of both the stannole and Cp^ttt^ ligands is reflected in the negative NICS values of −14.97 and −8.64 ppm, respectively (Figure S18). In **3_Y_**, whereas the Cp^ttt^ ligand retains a negative NICS value of −6.21 ppm, that of the stannole ligand is now + 25.29 ppm. The loss in the aromatic character of the stannole is, presumably, in favour of the delocalized bonding in the {Y_2_Sn_2_} ring system.

**Figure 4:**
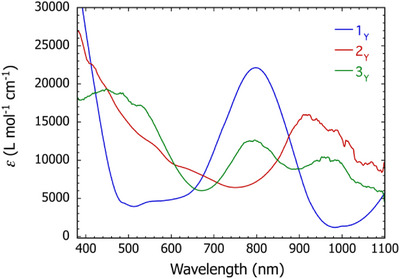
UV/vis spectra of **1_Y_** ·toluene (blue), [K(2.2.2‐crypt)][**2_Y_**]·1.5(hexane) (red), and [K(2.2.2‐crypt)]_2_[**3_Y_**]·3(THF) (green) in THF.

The electronic structure of the yttrium–stannole complexes was investigated further using UV/visible spectroscopy and time‐dependent DFT calculations. The UV/vis spectrum of **1_Y_** in THF (Figure 4) consists of a single major absorption centred on *λ*_max_ = 795 nm, which can be assigned to a HOMO‐to‐LUMO excitation calculated at 760 nm according to the TD‐DFT analysis (Figure S19, Table S7). The major absorption in the UV/vis spectrum of [K(2.2.2‐crypt)][**2_Y_**] in THF at *λ*_max_ = 918 nm (Figure 4) corresponds to a SOMO‐to‐LUMO transition calculated to occur at 886 nm (Table S8). The UV/vis spectrum of [K(2.2.2‐crypt)]_2_[**3_Y_**] features three significant absorptions centered on *λ*_max_ = 965 nm, *λ*_max_ = 795 nm, and *λ*_max_ = 460 nm (Figure 4). The lower‐ and intermediate‐energy absorptions correspond to the HOMO–LUMO transition calculated at 1066 nm, and transitions from the HOMO and HOMO–1 to the LUMO, LUMO + 1 and LUMO + 2 calculated at 871 and 788 nm (Table S9). The higher energy transitions are from the from the HOMO to higher‐lying LUMOs calculated at 405–545 nm.

The UV/vis spectra of **1_Gd_** and **1_Dy_** are qualitatively similar to those of yttrium analogues, with major absorptions at *λ*_max_ = 800 and 805 nm, respectively (Figures S20, S21). Likewise, the UV/vis spectra of [K(2.2.2‐crypt)][**2_Gd_**] and [K(2.2.2‐crypt)][**2_Dy_**] are also comparable to the yttrium versions, with absorptions at *λ*_max_ = 922 and 889 nm, respectively. The UV/vis spectra of [K(2.2.2‐crypt)]_2_[**3_Gd_**] and [K(2.2.2‐crypt)]_2_[**3_Dy_**] display much weaker absorptions located around *λ*_max_ = 455, 670, and 980 nm for gadolinium, with barely discernible *λ*_max_ values for dysprosium. Overall, the similarities in the UV/vis spectra of the yttrium–, gadolinium– and dysprosium–stannole complexes indicate similarities in the orbital structure and relative energies, based on the TD‐DFT analysis of the yttrium versions.

### EPR Spectroscopy and DC Magnetic Properties

**Figure 5:**
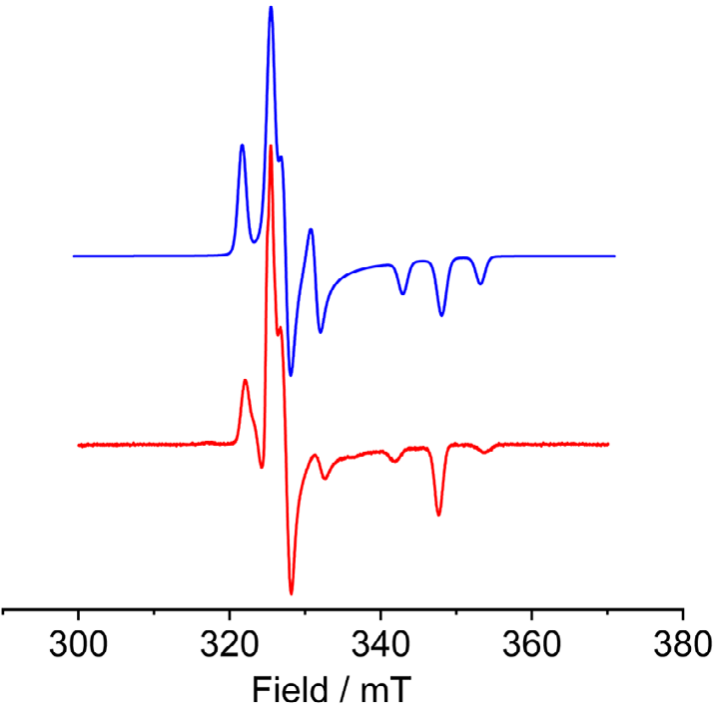
X‐Band EPR spectrum of [K(2.2.2‐crypt)][**2_Y_**] in 2‐Me‐THF at 100 K (red) and simulated spectrum (blue) using the parameters stated in the text.

The X‐band EPR spectrum of [K(2.2.2‐crypt)][**2_Y_**] was recorded as a frozen solution in 2‐Me‐THF at 100 K and is anisotropic in nature due to delocalization of the unpaired electron (Figure 5). A simulation was achieved using *g_x_* = 2.057, *g_y_* = 2.058, and *g_z_* = 1.932, the anisotropic hyperfine coupling constants *A_x_* = 161 MHz, *A*_y_ = 30 MHz and *A*_z_ = 140 MHz for coupling to ^89^Y, and *A_x_* = 112 MHz, *A_y_* = 13 MHz and *A_z_* = 132 MHz for coupling to ^119^Sn, with anisotropic linewidths of 16, 30, and 22 MHz.

**Figure 6:**
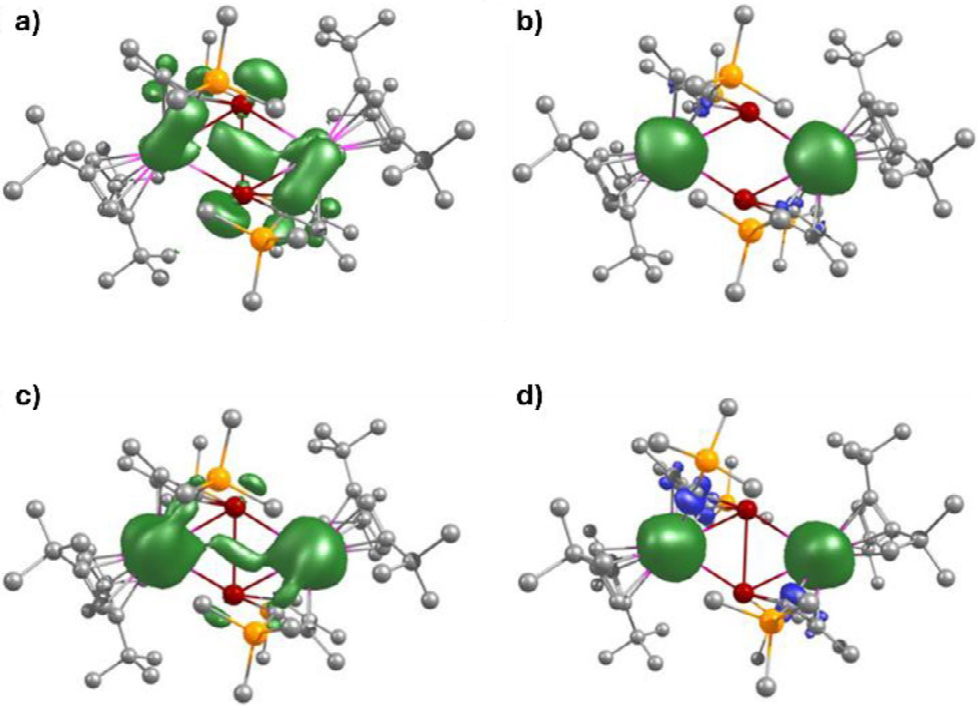
Spin density plots for a) **2_Y_**, b) **1_Gd_**, c) **2_Gd_**, and d) **3_Gd_**. The isosurface value is 0.0015 for **2_Y_** and 0.0026 for the gadolinium complexes.

Ddelocalization of the radical electron in [K(2.2.2‐crypt)][**2_Y_**] is reflected in a DFT calculation of the spin density in the anion **2_Y_**, which shows appreciable unpaired spin on the yttrium and tin atoms, including along the tin–tin axis (Figure 6). Similar calculations on the three gadolinium complexes revealed the spin density to reside solely on the Gd^3+^ ions in **1_Gd_** and **3_Gd_**, as expected, with additional spin density located within the {Gd_2_Sn_2_} ring of **2_Gd_** (Figure 6). The spin density properties of **2_Gd_** therefore prompted measurement of the magnetic susceptibility properties to determine the nature of the exchange coupling between gadolinium and the tin radical ligand.

**Figure 7:**
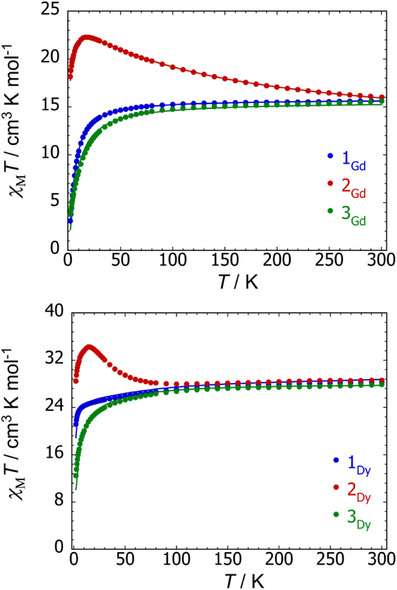
Plots of *χ*_M_ *T*(*T*) for the gadolinium–stannole (top) and dysprosium–stannole (bottom) complexes. Data were collected in an applied field of 1000 Oe. Solid lines for the gadolinium compounds represents fits to the data using the spin Hamiltonian parameters stated in the text. Solid lines for **1_Dy_** and **3_Dy_** represent ab initio simulations of the data using the parameters in the text.

The molar magnetic susceptibility (*χ*_M_) of the gadolinium– and dysprosium–stannole compounds was measured as a function of temperature in a DC field of 1000 Oe. For compounds **1_Gd_** and [K(2.2.2‐crypt)]_2_[**3_Gd_**], the plots of *χ*_M_ *T*(*T*) in the temperature range 2–300 K are characteristic of weak antiferromagnetic exchange coupling between the gadolinium centres (Figure 7). At 300 K, *χ*_M_ *T* is 15.63 cm^3^ K mol^−1^ for **1_Gd_** and 15.61 cm^3^ K mol^−1^ for [K(2.2.2‐crypt)]_2_[**3_Gd_**], similar to the theoretical value of 15.76 cm^3^ K mol^−1^ for two uncoupled Gd^3+^ ions (^8^S_7/2_ ground term). The susceptibility for both compounds then decreases gradually down to 20 K before reaching 3.07 and 3.77 cm^3^ K mol^−1^, respectively, at 2 K. In contrast, the radical‐bridged compound [K(2.2.2‐crypt)][**2_Gd_**] has a markedly different *χ*_M_ *T*(*T*) profile, with the susceptibility increasing from 16.02 cm^3^ K mol^−1^ at 300 K, close to the predicted value of 16.14 cm^3^ K mol^−1^ for two Gd^3+^ ions and a single unpaired electron,^[^ ^27^ ^]^ to a maximum of 22.28 cm^3^ K mol^−1^ at 18 K. The susceptibility then decreases to 18.17 cm^3^ K mol^−1^ at 2 K. These data indicate much stronger antiferromagnetic exchange coupling in [K(2.2.2‐crypt)][**2_Gd_**].

For **1_Gd_** and [K(2.2.2‐crypt)]_2_[**3_Gd_**], a −2*J* spin Hamiltonian formalism (Equation 1) was used to fit the data with terms to account for the gadolinium–gadolinium exchange (*J*_GdGd_), the Zeeman interaction and an intermolecular exchange term (*zJ*'). For [K(2.2.2‐crypt)][**2_Gd_**], an additional term (*J*_GdSn_) was used to account for the gadolinium‐radical exchange (Equation 2). The parameters used in the fits are presented in Table 1. The most striking result from this analysis is the very large *J*_GdSn_ value of –112 cm^−1^ for [K(2.2.2‐crypt)][**2_Gd_**], which is accompanied by an unusually large *J*_GdGd_ value of −5.13 cm^−1^. In stark contrast, the values of *J*_GdGd_ for **1_Gd_** and [K(2.2.2‐crypt)]_2_[**3_Gd_**] are more characteristic of the weak exchange coupling normally found in polynuclear gadolinium compounds,^[^ ^13^ ^]^ being −0.23 and −0.24 cm^−1^, respectively.

**Table 1:** Parameters used to fit the *χ*_M_ *T*(*T*) data for the gadolinium–stannole compounds using a −2*J* spin Hamiltonian formalism.

	1_Gd_	2_Gd_	3_Gd_
*g*	2.003	1.94	1.98
*J*_Gd‐Gd_/cm^−1^	−0.23	−5.13	−0.24
*J*_Gd‐Sn_/cm^−1^		−112	
*zJ*'/cm^−1^	−0.005	−0.006	−0.04

The *χ*_M_ *T*(*T*) data for the three dysprosium–stannole complexes follow a qualitatively similar profile to the gadolinium versions (Figure 7). For **1_Dy_** and [K(2.2.2‐crypt)]_2_[**3_Dy_**], the values of *χ*_M_ *T* at 300 K are 28.76 and 27.88 cm^3^ K mol^−1^, respectively, in good agreement with the expected value of 28.34 cm^3^ K mol^−1^ for two uncoupled Dy^3+^ ions (^6^H_15/2_ ground term). A steady decrease in *χ*_M_ *T* then occurs as the temperature is lowered to around 20 K, and then a sharper decrease in the susceptibility to 18.87 cm^3^ K mol^−1^ for **1_Dy_** and 12.47 cm^3^ K mol^−1^ for [K(2.2.2‐crypt)]_2_[**3_Dy_**] occurs at 2 K. For [K(2.2.2‐crypt)][**2_Dy_**], the value of *χ*_M_ *T* at 300 K is 28.60 cm^3^ K mol^−1^, close to the expected value of 28.7 cm^3^ K mol^−1^ for two uncoupled Dy^3+^ ions and an unpaired electron.^[^ ^27^ ^]^

(1)

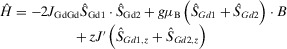



(2)

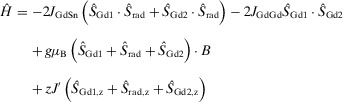




The value of *χ*_M_ *T* then steadily decreases as the temperature is lowered to 100 K, before increasing sharply to reach a maximum of 34.26 at cm^3^ K mol^−1^ 14 K, before decreasing to 28.48 cm^3^ K mol^−1^ at 2 K.

Simulations of the susceptibility data were achieved for **1_Dy_** and **3_Dy_** using the POLY_ANISO routine and the Ising‐type spin Hamiltonian stated in Equation 3. The resulting dipolar exchange coupling constants are *J*_dip_ = −0.006 cm^−1^ for **1_Dy_** and + 0.020 cm^−1^ for **3_Dy_**, with exchange coupling constants of *J*_exch_ = −0.028 and −0.32 cm^−1^, respectively. The total coupling in **1_Dy_** and **3_Dy_** is therefore *J*_tot_ = −0.034 and −0.30 cm^−1^, respectively. Although a simulation of the susceptibility for **2_Dy_** proved to be computationally intractable, the similarities with the data for **2_Gd_** suggest that the dysprosium‐radical exchange in **2_Dy_** should be considerably stronger than in **1_Dy_** and **3_Dy_**. The susceptibility properties of **1_Dy_** and **3_Dy_** are also remarkably like their germole‐ligated cousins,^[^ ^28^ ^]^ whereas those of **2_Dy_** show a more pronounced maximum in the *χ*_M_ *T*(*T*) data than the radical‐bridged germanium complex. This observation may reflect greater enhancement of the bonding across the {Dy_2_Sn_2_} unit upon one‐electron reduction, presumably due to the more diffused character of the tin 5p orbitals relative to the 4p orbitals of germanium.

(3) $$\;\hat{H}=-\left(J_{dip}+J_{ex}\right)\hat{{\tilde{S}}_{1,z}}\cdot \hat{{\tilde{S}}_{2,z}}$$

### Dynamic (AC) Magnetic Properties

Di‐ and tri‐metallic dysprosium complexes have proven to be invaluable for studying the impact of exchange coupling on SMM properties, particularly the effective energy barrier to reversal of the magnetization (*U*_eff_) and magnetic hysteresis.^[^ ^29^, ^30^, ^31^, ^32^, ^33^, ^34^, ^35^, ^36^, ^37^ ^]^ Whilst previous studies have focused on *N*‐ and *O*‐bridged multimetallic systems, several examples with μ‐bridging heavier p‐block elements (P, As, Sb, Bi, S, Se) have also been described.^[^ ^18^, ^38^, ^39^, ^40^, ^41^, ^42^ ^]^ Furthermore, the only tin‐ligated SMM is the monometallic erbium–stannole sandwich complex [(η^5^‐Cp^Sn^)Er(η^8^‐COT)]^−^ reported by us,^[^ ^22^ ^]^ with the properties of two lanthanide *bis*(stannole) complexes having been described in a recent preprint.^[^ ^43^ ^]^ Compounds **1_Dy_**, [K(2.2.2‐crypt)][**2_Dy_**] and [K(2.2.2‐crypt)]_2_[**3_Dy_**] therefore provide an opportunity to investigate how the unique {Dy_2_Sn_2_} delocalized bonding and the tin‐mediated exchange impact on the dynamic magnetic properties.

**Figure 8:**
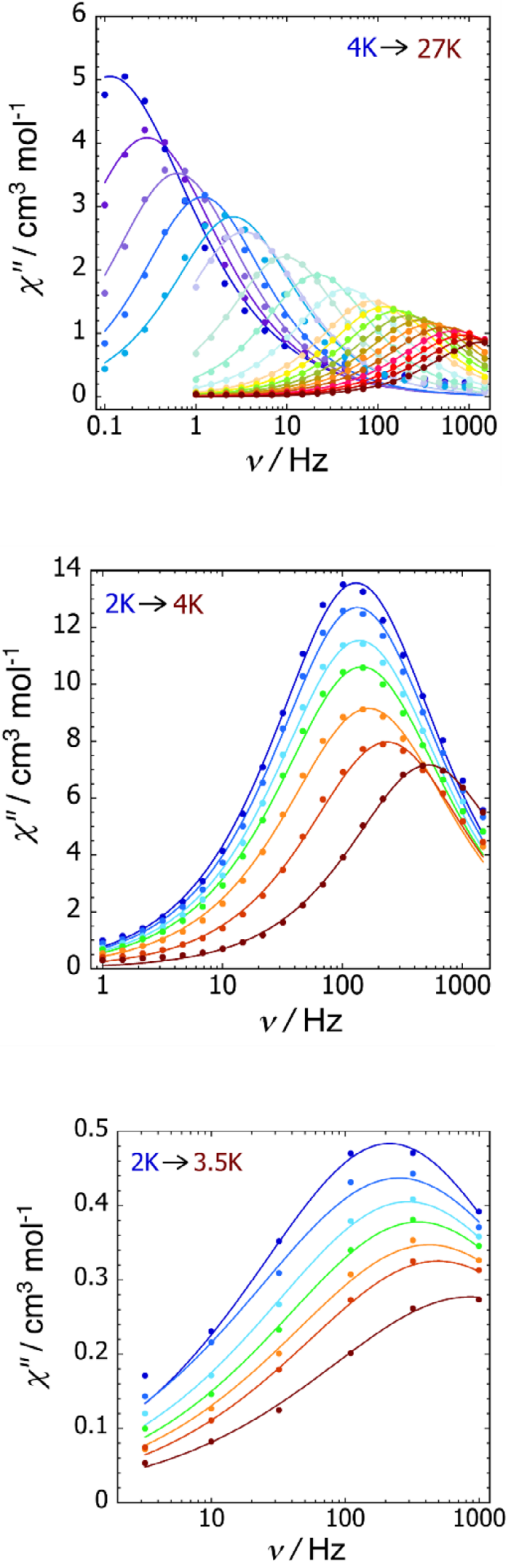
Plots of *χ*″ versus AC frequency (*ν*) in zero DC field for: **1_Dy_** (top), [K(2.2.2‐crypt)][**2_Dy_**] (centre) and [K(2.2.2‐crypt)]_2_[**3_Dy_**] (bottom) at the indicated temperatures. Solid lines indicate fits to a generalised Debye model.

In zero DC field, all three dysprosium–stannole dimetallic complexes give rise to maxima in the out‐of‐phase component of the AC susceptibility (*χ*″) as a function of frequency (*ν*) (Figures 8, S24–34). For **1_Dy_**, temperature‐dependent maxima were observed in the relatively wide temperature range 4–27 K, whereas for [K(2.2.2‐crypt)][**2_Dy_**] and [K(2.2.2‐crypt)]_2_[**3_Dy_**] the maxima were observed in much narrower ranges of 2–4 and 2–3.5 K, respectively, and the maxima show only a weak temperature dependence. Magnetic relaxation times (*τ*) were then extracted from Cole–Cole plots of *χ*″ versus the in‐phase component of the AC susceptibility *χ*′. The plot of ln *τ* versus *T* ^−1^ for **1_Dy_** is roughly linear at 22–27 K before showing curvature at lower temperatures (Figure S28, Table S10). These data suggest that at least Orbach and Raman relaxation processes occur, with some contribution from quantum tunnelling of the magnetization (QTM) at low temperatures. A fit of the relaxation time data for **1_Dy_** was obtained using Equation 4, in which *τ*_0_ is the attempt time, *C* is the Raman coefficient, *n* is the Raman exponent and $\tau_{\mathrm{QTM}}^{-1}$ is the rate of QTM. This analysis gave *U*_eff_ = 138 ± 7 cm^−1^, *τ*_0_ = 1.44 × 10^−7^ s, *C* = 6.2 × 10^−4^ ± 10^−4^ s^−1^ K^−^ *^n^*, *n* = 4.8 ± 0.1, and *τ*_QTM_ = 1.97 ± 0.34 s.

(4) $$\;\tau^{-1}=\tau_{0}^{-1}e^{-U_{\mathrm{eff}}/k_{B}T}+CT^{n}+\tau_{\mathrm{QTM}}^{-1}$$

The ln *τ* versus *T* ^−1^ plot for [K(2.2.2‐crypt)][**2_Dy_**] has more pronounced curvature (Figure S32, Table S11), with the fit using Equation 4 producing *U*_eff_ = 36 ± 1.4 cm^−1^, *τ*_0_ = 1.14 × 10^−9^ s, *C* = 5.4 ± 1.6 s^−1^ K^−^ *^n^*, *n* = 3.5 ± 0.25, and *τ*_QTM_ = 1.33 × 10^−3^ ± 1.41 × 10^−5^ s. In contrast, a fit of the relaxation time data for [K(2.2.2‐crypt)]_2_[**3_Dy_**] was possible using only the Raman and QTM terms (Figure S36, Table S12), resulting in *C* = 19.9 ± 7.0 s^−1^ K^−^ *^n^*, *n* = 4.3 ± 0.3, and *τ*_QTM_ = 1.035 × 10^−3^ ± 9.5 x 10^−4^ s.

The prominent QTM in all three dysprosium–stannole complexes indicates fast relaxation of the magnetization even at low temperatures and implies that none of these compounds should show magnetic hysteresis (memory) effects. This was confirmed by measuring the dynamic field‐dependence of the magnetization at 2 K in each case (Figures S37–S40). For **1_Dy_**, using field sweep rates in the range 0.23–11.6 mT s^−1^, the magnetization (*M*) versus field (*H*) loops shows a very slight opening at 2 K around zero field, closing at higher temperatures. The *M*(*H*) loops for [K(2.2.2‐crypt)][**2_Dy_**] are butterfly‐shaped and closed around zero field, whereas for [K(2.2.2‐crypt)]_2_[**3_Dy_**] no discernible opening of the hysteresis loops occurs.

The decrease in *U*_eff_ value from **1_Dy_** to [K(2.2.2‐crypt)][**2_Dy_**], and the absence of a measurable barrier in [K(2.2.2‐crypt)]_2_[**3_Dy_**], can be interpreted qualitatively in terms of the changes in molecular structure that occur across the dysprosium–stannole units with each reduction. With the contraction of the {Dy_2_Sn_2_} rings across the series and the build‐up of electron density on and between the tin atoms, the equatorial component of the crystal field is enhanced. Simultaneously, the Dy–(Cp^ttt^)_cent_ interactions lengthen, which weakens the axial crystal field from **1_Dy_** to **2_Dy_**, and again from **2_Dy_** to **3_Dy_**. For the oblate spheroidal ion Dy^3+^, the structural changes should be detrimental to the SMM properties, as found in other dysprosium metallocene SMMs.^[^ ^44^, ^45^, ^46^ ^]^

### Multireference Calculations

To provide a more detailed basis for the variation in the SMM properties of the dysprosium–stannole complexes, multireference calculations were performed on **1_Dy_** and **3_Dy_** using the atomic coordinates obtained from the crystallographic studies. All calculations were performed using the ORCA 6.0.0 software package.^[^ ^24^, ^25^ ^]^ Calculations on the radical‐bridged system **2_Dy_** were not carried out owing to the prohibitive computational cost.

**Figure 9:**
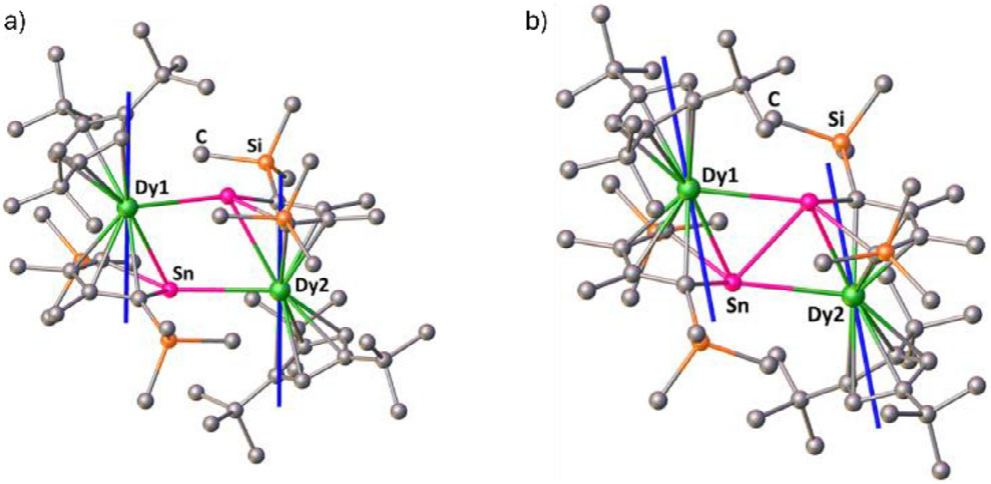
Easy axes of magnetization (blue lines) in the ground Kramers doublets of the Dy^3+^ ions in **1_Dy_** (left) and **3_Dy_** (right).

For **1_Dy_**, the easy axes of magnetization in the ground Kramers doublet (KD) for each Dy^3+^ ion are oriented towards the centre of the [η^5^‐Cp^Sn^]^2−^ ligand rather than the [η^5^‐Cp^ttt^]^−^ ligand, presumably because of the greater formal charge on the former (Figure 9). The *g*‐tensors associated with the ground KD of Dy1 in **1_Dy_** are *g_x_* = 0.0018, *g_y_* = 0.0026, and *g_z_* = 19.70, and for Dy2 they are *g_x_* = 0.0020, *g_y_* = 0.0029, and *g_z_* = 19.69 (Tables S16, S17). The strong axial character of the ground KDs in **1_Dy_** is emphasized by the wavefunction compositions of greater than 96% |*M_J_* | = 15/2 for both dysprosium centres. The first‐excited KDs in **1_Dy_** both lie at 200 cm^−1^ above the ground KD and feature larger contributions from the transverse components of the *g*‐tensors, along with significant mixing of wavefunctions. These properties indicate that Orbach relaxation should occur via the first‐excited KD, as reflected in the calculated relaxation barriers for this system (Figures S42, S43). Although the experimental barrier is somewhat lower than predicted by the calculations, it is possible that the discrepancy is related to electron correlation effects outside of the dysprosium 4f manifold.

In **3_Dy_**, the orientation of the easy axes of magnetization shifts towards the centre of the Cp^ttt^ ligands and, consequently, towards one of the Sn─C bonds of the stannole ligand (Figure 9), which could be due to delocalization of electron density from the stannole ring into the {Dy_2_Sn_2_} ring. Significantly reduced axial character of the ground KDs in **3_Dy_** is reflected in the associated *g*‐tensors of *g_x_* = 0.0365/0.0366, *g_y_* = 4.693/4.665 and *g_z_* = 14.51/14.54, with the associated wavefunctions being strong admixture of states (Tables S18, S19). As such, fast QTM within the ground KD is expected to be the dominant relaxation mechanism in **3_Dy_**, as shown in the calculated relaxation barriers (Figures S44, S45), consistent with experimental observations.

## Discussion

Compounds containing bonds between tin and rare‐earth elements form a relatively small family that can be divided into two types. The first consists of coordination and organometallic compounds of interest for covalency in the metal–metal bonds and their associated reactivity.^[^ ^47^, ^48^, ^49^, ^50^, ^51^, ^52^, ^53^, ^54^ ^]^ The second type is composed of structurally elaborate intermetallic clusters with unusual electronic properties,^[^ ^55^, ^56^, ^57^ ^]^ which can enable applications in lithography and as magnetic materials.^[^ ^58^, ^59^ ^]^ By comparison, f‐element metallole chemistry, especially with stannole ligands, is conspicuously under‐developed despite the availability of reliable synthetic methods for a variety of these heavy cyclopentadienyl analogues.^[^ ^28^, ^60^, ^61^, ^62^, ^63^, ^64^, ^65^, ^66^ ^]^ Furthermore, surprisingly few η^5^‐stannole complexes of transition metals have been reported,^[^ ^67^, ^68^, ^69^, ^70^, ^71^, ^72^, ^73^, ^74^ ^]^ emphasizing that the coordination chemistry of this ligand is still to be explored.

The one‐ and two‐electron reduction of **1** **_M_** to give the tin–tin bonded complexes **2** **_M_** and **3** **_M_**, respectively, are unusual examples of stannole coupling to give di‐stannole compounds. The reactivity of **1** **_M_** is reminiscent of the tin–tin coupling reactions of dilithio‐stannoles to give di‐stannole compounds, although these reactions typically occur under oxidative, rather than reductive, conditions.^[^ ^75^, ^76^, ^77^, ^78^, ^79^, ^80^, ^81^, ^82^, ^83^ ^]^ Whilst **1** **_M_**, **2** **_M_**, and **3** **_M_** are reminiscent of their germanium analogues^[^ ^21^ ^]^ and of a related titanium(III) germole complex with delocalized {Ti_2_Ge_2_} bonding,^[^ ^84^ ^]^ their similar chemistry is perhaps surprising given the expected weaker nature of tin‐tin bonds. The valence d‐orbitals of the rare‐earth metals evidently play a critical structural role in supporting the highly unusual delocalized {M_2_Sn_2_} bonding in **2** **_M_** and **3** **_M_**.

To the best of our knowledge, the tin‐based radicals in **2** **_M_** are not only unprecedented as ligands in f‐element chemistry, but they are also unknown in transition metal chemistry. The tin–tin bond in **2** **_M_** shares some characteristics with the unusual single‐electron bond formed upon one‐electron reduction of the di‐amido stannylene [Sn(NDipp)_2_C_6_H_4_], forming the di‐tin radical anion [C_6_H_4_(DippN)_2_Sn⋅⋅⋅Sn(NDipp)_2_C_6_H_4_]^−^ (Dipp = 2,6‐diisopropylphenyl).^[^ ^85^ ^]^ Whereas structurally authenticated monometallic tin(I) and tin(III) radicals are relatively numerous,^[^ ^86^, ^87^, ^88^, ^89^, ^90^ ^]^ complex **2** **_M_** is a rare example of a di‐tin radical.^[^ ^91^, ^92^, ^93^ ^]^

The gadolinium–tin exchange coupling constant of *J* = −112 cm^−1^ in **2_Gd_** is one of the largest reported for a lanthanide complex. Although larger coupling constants have been determined, including a record of 387(4) cm^−1^, these exceptionally strong interactions are based on direct lanthanide–lanthanide bonds rather than exchange between a lanthanide and a radical ligand.^[^ ^94^, ^95^, ^96^, ^97^, ^98^ ^]^ Indeed, strong lanthanide‐radical exchange couplings involving *S* = 1/2 ligands such as [N_2_]^3−^ or *N*‐heterocycles are typically in the range of 20–30 cm^−1^, making the exchange in **2_Gd_** unusually strong for this type of complex.^[^ ^99^ ^]^ It is also noteworthy that the only significant difference in the magnetic properties of **2_Gd_** and its isostructural germole analogue is the smaller exchange coupling of −95 cm^−1^,^[^ ^21^ ^]^ highlighting that lanthanide‐radical coupling can be tuned by varying the group 14 element. Furthermore, the ability of heavy p‐block radical ligands to enhance exchange interactions between lanthanide centres is also reflected in the large *J*_GdGd_ value of −5.13 cm^−1^ for **2_Gd_**, which is comparable in magnitude to the analogous parameter of approximately −1.92 cm^−1^ found in a bismuth‐based radical‐bridged di‐lanthanide complex.^[^ ^18^ ^]^

## Conclusion

The dimeric rare‐earth stannole complexes [(η^5^‐Cp^Sn^)M(η^5^‐Cp^ttt^)]_2_ (**1** **_M_**, M = Y, Gd, Dy) undergo one‐ and two‐electron reduction to give the corresponding complex anions [{(η^5^‐Cp^Sn^)M(η^5^‐Cp^ttt^)}_2_]^−^ (**2** **_M_**) and [{(η^5^‐Cp^Sn^)M(η^5^‐Cp^ttt^)}_2_]^2−^ (**3** **_M_**), respectively, as salts of [K(2.2.2‐crypt)]^+^ in good yields. Crystallographic studies reveal a contraction of the central {M_2_Sn_2_} rings with each reduction, consistent with the population of a bonding molecular orbital. A loss in the aromaticity of the stannole rings in **3** **_M_** is also suggested by the structural studies and is supported by the determination of a positive NICS(0) value for **3_Y_**. Bonding analysis of the yttrium‐ and gadolinium‐stannoles reveals the frontier MOs to have appreciable contributions from the valence d‐orbitals of the rare‐earth metals and the tin 5p orbitals. Reduction of **1** **_M_** to give the tin radical complexes **2_M_** results in population of a SOMO that spans the {M_2_Sn_2_} rings and the stannole ligand, with appreciable end‐on overlap between tin 5p orbitals to give a tin‐tin bond. Further reduction to give **3** **_M_** enhances the M─Sn and Sn─Sn bonding, reflected in increased WBI values. Magnetic measurements on **2_Gd_** reveal an extremely large coupling of −112 cm─^1^ between gadolinium and the tin radical, and EPR measurements of **2_Y_** reveal extensive hyperfine interactions spanning the yttrium and tin centres, consistent with extensive delocalization of spin density.

With the stannole ligand now established in rare‐earth chemistry, the next phase in our investigations will focus on the reactivity of these compounds as bimetallic reducing agents.

## Supporting Information

Synthesis, FTIR, NMR and UV/vis spectra, X‐ray crystallography details, magnetic measurements, EPR spectroscopy, DFT and multireference calculation details. Additional research data supporting this publication are available as supplementary information at DOI: 10.25377/sussex.29634422. The authors have cited additional references within the Supporting Information.^[^ ^100^, ^101^, ^102^, ^103^, ^104^, ^105^, ^106^, ^107^, ^108^, ^109^, ^110^, ^111^, ^112^, ^113^, ^114^, ^115^, ^116^, ^117^, ^118^, ^119^, ^120^ ^]^

## Conflict of Interests

The authors declare no conflict of interest.

## Supporting information



Supporting Information

Supporting Information

## Data Availability

The data that support the findings of this study are openly available in Figshare at https://doi.org/10.25377/sussex.29634422, reference number 1.
