# Supplementary material for: Reduction of Rare‐Earth Stannole Sandwich Complexes to Tin‐Based Radical Ligands and Tin–Tin Bonds
Source: Angew Chem Int Ed Engl. 2025 Sep 14;64(45):e202516323. doi: 10.1002/anie.202516323 (PMC12582010; doi:10.1002/anie.202516323)
Supplement: Supplementary file 1 — Supporting Information [file ANIE-64-e202516323-s002.docx]

*Supporting Information*

**Reduction of Rare-Earth Stannole Sandwich Complexes to**

**Tin-Based Radical Ligands and Tin-Tin Bonds**

Siddhartha De,^a^ Arpan Mondal,^a^ Jinkui Tang,^b^ Richard A. Layfield*^,a^

1. Department of Chemistry, School of Life Sciences, University of Sussex, Brighton, BN1 9QR, U.K.

[r.layfield@sussex.ac.uk](mailto:r.layfield@sussex.ac.uk)

1. Changchun Institute of Applied Chemistry, Chinese Academy of Sciences, Renmin Street 5626, 130022 Changchun, China.

**Contents**

General Considerations and Synthesis Protocols S1-S3

X-ray Crystallography S4-S10

FTIR Spectroscopy S11-S13

NMR Spectroscopy S14-S23

DFT Calculations S24-S30

UV/vis Spectra S31-S32

Magnetic Measurements S33-S45

Multireference Calculations S46-S56

References S57

**General considerations**

All experiments were performed under rigorous anhydrous and anaerobic conditions using standard Schlenk line techniques and argon-filled gloveboxes. Solvents were refluxed over a suitable drying agent for a minimum of three days (molten potassium for toluene and THF, Na/K alloy for hexane), and then distilled, degassed via several freeze-pump-thaw cycles, and stored in J. Young flasks over potassium mirrors (toluene and hexane) or activated 4 Å molecular sieves (THF). Literature procedure was employed to synthesise [(η^5^-Cp^ttt^)M(BH_4_)_2_(THF)] (M = Dy, Gd, and Y) and [K_2_Cp^Sn^].^[22,100]^ All other reagents were purchased from commercial sources and used without further purification. Glass-coated stirrer bars were used in all reactions.

NMR experiments were performed using J. Young valve NMR tubes and samples were prepared inside the glovebox. Both Toluene-D_8_ and THF-D_8_ were dried over potassium and degassed by three freeze-pump-thaw cycles. NMR spectra were recorded on a Varian VNMR S400 spectrometer (^1^H, ^29^Si, ^13^C, and ^119^Sn) operating at 25°C and frequencies of 400.13 MHz (^1^H), 100.62 MHz (^13^C), 79.45 MHz (^29^Si), and 149.21 MHz (^119^Sn). The ^1^H and ^13^C chemical shifts were calibrated to the residual signals from the deuterated solvent. The multiplicity of the signals is indicated as s = singlet, d = doublet, dd = doublet of doublets, t = triplet, q = quartet and m = multiplet. UV-vis spectra were recorded using a PerkinElmer LAMBDA 265 spectrophotometer using J. Young adapted quartz cuvettes and baselines were corrected by a blank sample of THF in a quartz cuvette. X-band EPR spectra were recorded at the PEPR Facility at Imperial College London in CW mode on a Bruker EMX spectrometer equipped with a Bruker ER049X SuperX microwave bridge, a Bruker ER4122SHQE resonator, and an Oxford Instruments ITC503 temperature controller.

Elemental analyses were carried out at Elemental Microanalysis Ltd, Devon, United Kingdom. ATR-FTIR spectra were recorded using a Bruker ALPHA spectrometer equipped with a Platinum ATR module in a glovebox.

**Synthesis of [(η^5^-Cp^ttt^)Y(η^5^-Cp^Sn^)]_2_**·**toluene (1_Y_**·**toluene)**

Solid [K_2_Cp^Sn^·THF] (74 mg, 0.15 mmol) and [(η^5^-Cp^ttt^)Y(BH_4_)_2_(THF)] (64 mg, 0.15 mmol) were suspended in a mixture of hexane-toluene (20 mL, 3:1) at room temperature. The suspension was allowed to stir at room temperature for a few minutes and then heated at 110°C for 48 hours. The resulting green suspension was cooled down to room temperature and the solvent was removed under reduced pressure. Then, the residue was extracted with hot hexane. The hexane-soluble fraction was evaporated to dryness and the solid was dissolved in the minimum volume of toluene and stored at –35 °C. Single crystals of **1_Y_**·toluene were isolated after four days (60 mg, 56%). **Elemental analysis** (%) calculated for C_65_H_114_Y_2_Sn_2_Si_4_: C, 54.86; H, 8.07. Found: C, 54.48; H, 8.29. **^1^H NMR (toluene-D_8_, *δ* /ppm):** 0.06 (36H, s, Si(C**H**_3_)_3_, Cp^Sn^), 1.28 (18H, s, ^t^Bu), 1.49 (36H, s, ^t^Bu), 2.70 (12H, s, C**H**_3_, Cp^Sn^), 6.42 (4H, s, C_5_**H**_2_^t^Bu_3_). **^13^C{^1^H} NMR (toluene-D_8_, *δ*/ppm):** 4.94 (Si(**C**H_3_)_3_, Cp^Sn^), 26.83(**C**H_3_, Cp^Sn^), 32.72 (Cp^ttt^ 4-C(**C**H_3_)_3_), 33.35(Cp^ttt^ 1,2- C(**C**H_3_)_3_), 34.66 (Cp^ttt^ 4-**C**Me_3_), 35.15(Cp^ttt^ 1,2-**C**Me_3_), 135.90 (Cp^ttt^ 1,2-C_5_ ring), 135.93 (Cp^ttt^ 4-C_5_ ring), 137.57 (**C**_β_, Cp^Sn^), 150.68 (**C**_α_, Cp^Sn^). **^29^Si{^1^H} HMBC NMR (toluene-D_8_, *δ*/ppm):** –8.42. **^119^Sn{^1^H} NMR (toluene-D_8_, *δ*/ppm):** 528.07, 531.02.

**Synthesis of [(η^5^-Cp^ttt^)Gd(η^5^-Cp^Sn^)]_2_**·**toluene (1_Gd_**·**toluene)**

Compound **1_Gd_**·toluene was prepared following the same procedure as for **1_Y_**·toluene, using [K_2_Cp^Sn^·THF] (74 mg, 0.15 mmol) and [(η^5^-Cp^ttt^)Gd(BH_4_)_2_(THF)] (74 mg, 0.15 mmol). Yield: 64 mg, 55%. **Elemental analysis** (%) calculated for C_65_H_114_Gd_2_Sn_2_Si_4_: C, 50.05; H, 7.37. Found: C, 50.37; H, 7.14.

**Synthesis of [(η^5^-Cp^ttt^)Dy(η^5^-Cp^Sn^)]_2_**·**toluene (1_Dy_**·**toluene)**

Compound **1_Dy_**·toluene was prepared following the same procedure as for **1_Y_**·toluene, using [K_2_Cp^Sn^·THF] (74 mg, 0.15 mmol) and [(η^5^-Cp^ttt^)Dy(BH_4_)_2_(THF)] (75 mg, 0.15 mmol). Yield: 60 mg, 51%. **Elemental analysis** (%) calculated for C_65_H_114_Dy_2_Sn_2_Si_4_: C, 49.71; H, 7.32. Found: C, 49.89; H, 7.32.

**Synthesis of [K(2.2.2-crypt)][(η^5^-Cp^ttt^)Y(η^5^-Cp^Sn^)]_2_**·**1.5(hexane) ([K(2.2.2-crypt)][2_Y_]·1.5(hexane))**

Compound **1_Y_**·toluene (14.3 mg, 0.01 mmol) was dissolved in THF (4 mL) at room temperature. KC_8_ (1.4 mg, 0.01 mmol) and 2.2.2-cryptand (3.8 mg, 0.01 mmol) were added to the green solution. The colour was changed immediately to dark red, and the solution was stirred at room temperature for 15 minutes. The red solution was filtered, layered with hexane and stored at –35 °C. Crystals of [K(2.2.2-crypt)][**2_Y_**]·1.5(hexane) were obtained after six days (11 mg, 58%). **Elemental analysis** (%) calculated for C_85_H_163_Y_2_Sn_2_Si_4_N_2_O_6_K: C, 54.42; H, 8.76; N, 1.49. Found: C, 54.35; H, 8.42; N, 1.53. **^1^H NMR (THF-D_8_, *δ* /ppm):** 2.58 (s, 2.2.2-cryptand), 3.55 (s, 2.2.2-cryptand), 3.60 (s, 2.2.2-cryptand).

**Synthesis of [K(2.2.2-crypt)][(η^5^-Cp^ttt^)Gd(η^5^-Cp^Sn^)]_2_** ·**1.5(hexane) ([K(2.2.2-crypt)][2_Gd_]·1.5(hexane))**

Compound [K(2.2.2-crypt)][**2_Gd_**]·1.5(hexane) was synthesised using the procedure described for [K(2.2.2-crypt)][**2_Y_**]·1.5(hexane), using **1_Gd_**·toluene (15.6 mg, 0.01 mmol), KC_8_ (1.4 mg, 0.01 mmol) and 2.2.2-cryptand (3.8 mg, 0.01 mmol). Yield: 13 mg, 65%. **Elemental analysis** (%) calculated for C_85_H_163_Gd_2_Sn_2_Si_4_N_2_O_6_K: C, 50.73; H, 8.16; N, 1.39. Found: C, 50.99; H, 8.20; N, 1.61.

**Synthesis of [K(2.2.2-crypt)][(η^5^-Cp^ttt^)Dy(η^5^-Cp^Sn^)]_2_** ·**1.75(hexane) ([K(2.2.2-crypt)][2_Dy_]·1.75(hexane))**

Compound [K(2.2.2-crypt)][**2_Dy_**]·1.75(hexane) was synthesised using the procedure described for [K(2.2.2-crypt)][**2_Y_**]·1.5(hexane), using **1_Dy_**·toluene (15.7 mg, 0.01 mmol), KC_8_ (1.4 mg, 0.01 mmol) and 2.2.2-cryptand (3.8 mg, 0.01 mmol). Yield: 13 mg, 63%. **Elemental analysis** (%) calculated for C_86.5_H_166.5_Dy_2_Sn_2_Si_4_N_2_O_6_K: C, 50.81; H, 8.21; N, 1.37. Found: C, 51.52; H, 8.10; N, 1.47.

**Synthesis of [K(2.2.2-crypt)]_2_[(η^5^-Cp^ttt^)Y(η^5^-Cp^Sn^)]_2_**·**3(THF) ([K(2.2.2-crypt)]_2_[3_Y_]·3(THF))**

Compound **1_Y_**·toluene (14.3 mg, 0.01 mmol) was dissolved in 4 mL THF at room temperature and KC_8_ (2.9 mg, 0.021 mmol) and 2.2.2-cryptand (7.7 mg, 0.02 mmol) were added to the green solution. The colour of the solution changed to brown-red, and the solution was stirred for 30 minutes at room temperature. The brown-red mixture was filtered, layered with hexane and stored at –35 °C. Dark crystals of [K(2.2.2-crypt)]_2_[**3_Y_**]·3(THF) were obtained after seven days (15 mg, 63%). **Elemental analysis** (%) calculated for C_106_H_202_K_2_N_4_O_15_Si_4_Sn_2_Y_2_: C, 53.53; H, 8.56; N, 2.36. Found: C, 53.80; H, 8.57; N, 2.44. **^1^H NMR (THF-D_8_, *δ* /ppm):** –0.25 (36H, s, Si(C**H**_3_)_3_, Cp^Sn^), 1.02 (18H, s, ^t^Bu), 1.54 (36H, s, tBu), 2.58 (s, 2.2.2-cryptand), 3.54 (s, 2.2.2-cryptand), 3.61 (s, 2.2.2-cryptand), 5.70 (4H, s, C_5_**H**_2_^t^Bu_3_). **^29^Si{^1^H} HMBC THF-D_8_, *δ* /ppm):** –11.41.

**Synthesis of [K(2.2.2-crypt)]_2_[(η^5^-Cp^ttt^)Gd(η^5^-Cp^Sn^)]_2_**·**2(hexane) ([K(2.2.2-crypt)]_2_[3_Gd_]·2(hexane))**

Compound [K(2.2.2-crypt)]_2_[**3_Gd_**]·2(hexane) was synthesised using the procedure described for [K(2.2.2-crypt)]_2_[**3_Y_**]·3(THF), using **1_Gd_**·toluene (15.6 mg, 0.01 mmol), KC_8_ (2.9 mg, 0.02 mmol) and 2.2.2-cryptand (7.7 mg, 0.02 mmol). Yield: 13 mg, 52%. **Elemental analysis** (%) calculated for C_106_H_206_Gd_2_Sn_2_Si_4_N_4_O_12_K_2_: C, 51.12; H, 8.4; N, 2.27. Found: C, 51.69; H, 8.56; N, 2.32.

**Synthesis of [K(2.2.2-crypt)]_2_[(η^5^-Cp^ttt^)Dy(η^5^-Cp^Sn^)]_2_**·**2hexane ([K(2.2.2-crypt)]_2_[3_Dy_]·2(hexane))**

Compound [K(2.2.2-crypt)]_2_[**3_Dy_**]·2(hexane) was synthesised using the procedure described for [K(2.2.2-crypt)]_2_[**3_Y_**]·3(THF), using **1_Dy_**·toluene (15.7 mg, 0.01 mmol), KC_8_ (2.9 mg, 0.02 mmol) and 2.2.2-cryptand (7.7 mg, 0.02 mmol). Yield: 15 mg, 60%. **Elemental analysis** (%) calculated for C_106_H_206_Dy_2_Sn_2_Si_4_N_4_O_12_K_2_: C, 51.3; H, 8.37; N, 2.26. Found: C, 51.43; H, 8.52; N, 2.41.

**Oxidation of [K(2.2.2-crypt)][2_Y_]·1.5(hexane) using AgPF_6_**

A dark red solution of [K(2.2.2-crypt)][**2_Y_**]·1.5(hexane) (1.12 mg, 0.0006 mmol) in THF-D_8_ (0.6 ml) was added to a 6 mM THF solution (0.1 ml) of AgPF_6_ and the mixture was stirred for 5 minutes at room temperature. The colour of the solution immediately changed to green and the resulting the green solution was analysed via ^1^H NMR spectroscopy. Analysis of the reaction mixture revealed the formation of **1_Y_**.

**Reduction of [K(2.2.2-crypt)][2_Y_]·1.5(hexane) using KC_8_ and 2.2.2-cryptand:**

A dark red solution of [K(2.2.2-crypt)][**2_Y_**]·1.5(hexane) (13.9 mg, 0.0074 mmol) in THF-*d*_8_ (1.2 ml) was added to a mixture of KC_8_ (1mg, 0.0074 mmol) and 2.2.2-cryptand (2.8 mg, 0.0074 mmol). The resulting reddish-brown mixture was stirred for five minutes at room temperature. The solution was analysed via ^1^H NMR spectroscopy, which revealed the formation of [K(2.2.2-crypt)]_2_[**3_Y_**].

**X-Ray Crystallography**

Single crystals of all complexes were immersed in NVH oil (degassed and dried) and mounted on a Bruker D8 Venture Metaljet diffractometer and measured using Ga-K_α_ radiation (*λ* = 1.34139 Å). Crystals were kept at 100.0 K during data collection. Using Olex2,^[101]^ structures were solved with the olex2.solve^[102]^ structure solution program using charge flipping and refined with the SHELXL^[103]^ refinement package using least squares minimization.

**Table S1.** Crystal data for **1_Dy_**·toluene, **1_Gd_**·toluene, and **1_Y_**·toluene.

|  | **1_Y_·toluene** | **1_Gd_·toluene** | **1_Dy_·toluene** |
| --- | --- | --- | --- |
| CCDC Number | 2388027 | 2388026 | 2388025 |
| Empirical formula | C_65_H_114_Si_4_Sn_2_Y_2_ | C_65_H_114_Gd_2_Si_4_Sn_2_ | C_65_H_114_Dy_2_Si_4_Sn_2_ |
| Formula weight/g mol^–1^ | 1423.12 | 1559.80 | 1570.30 |
| Crystal system | triclinic | triclinic | triclinic |
| Space group | *P*$\bar{1}$ | *P*$\bar{1}$ | *P*$\bar{1}$ |
| *a*/Å | 11.9834(4) | 11.9707(6) | 12.0370(7) |
| *b*/Å | 12.2122(5) | 12.2044(6) | 12.2190(8) |
| *c*/Å | 24.6054(9) | 24.5523(12) | 24.4088(15) |
| *α*/° | 97.080(2) | 97.051(2) | 96.564(3) |
| *β*/° | 97.076(2) | 97.145(2) | 96.770(2) |
| *γ*/° | 99.770(2) | 99.707(2) | 99.414(3) |
| *V*/Å^3^ | 3483.1(2) | 3470.1(3) | 3484.1(4) |
| *Z* | 2 | 2 | 2 |
| *ρ*_calc_/g cm^-3^ | 1.357 | 1.493 | 1.497 |
| *μ*/mm^‑1^ | 5.905 | 14.321 | 15.421 |
| *F*(000) | 1476.0 | 1576.0 | 1584.0 |
| Crystal size/mm^3^ | 0.04 × 0.03 × 0.02 | 0.12 × 0.08 × 0.04 | 0.05 × 0.04 × 0.03 |
| 2*θ* range for data collection/° | 6.584 to 110.056 | 3.19 to 110.428 | 6.404 to 110.308 |
| Index ranges | -14 ≤ h ≤ 14, -14 ≤ k ≤ 14, -30 ≤ l ≤ 30 | -14 ≤ h ≤ 14, -14 ≤ k ≤ 14, -29 ≤ l ≤ 29 | -14 ≤ h ≤ 14, -14 ≤ k ≤ 14, -29 ≤ l ≤ 29 |
| Reflections collected | 74490 | 146756 | 68614 |
| Independent reflections | 13195 [R_int_ = 0.0842, R_sigma_ = 0.0533] | 13087 [R_int_ = 0.0413, R_sigma_ = 0.0250] | 13243[R_int_ = 0.0711, R_sigma_ = 0.0497] |
| Data/restraints/parameters | 13195/0/693 | 13087/0/693 | 13243/36/629 |
| Goodness-of-fit on *F*^2^ | 1.031 | 1.076 | 1.122 |
| Final *R* indexes [*I>=2σ (I)*] | R_1_ = 0.0326  wR_2_ = 0.0681 | R_1_ = 0.0269  wR_2_ = 0.0712 | R_1_ = 0.0484  wR_2_ = 0.1272 |
| Final *R* indexes [all data] | R_1_ = 0.0501  wR_2_ = 0.0735 | R_1_ = 0.0272  wR_2_ = 0.0714 | R_1_ = 0.0561  wR_2_ = 0.1330 |
| Largest diff. peak/hole / eÅ^-3^ | 1.01/-0.72 | 0.76/-1.53 | 1.01/-1.77 |

**Table S2.** Crystal data for [K(2.2.2-crypt)][**2_Dy_**]·1.75(hexane), [K(2.2.2-crypt)][**2_Gd_**]·1.5(hexane), and [K(2.2.2-crypt)][**2_Y_**]·1.5(hexane).

|  | **[K(2.2.2-crypt)][2_Y_]·**  **1.5(hexane)** | **[K(2.2.2-crypt)][2_Gd_]·**  **1.5(hexane)** | **[K(2.2.2-crypt)][2_Dy_]·**  **1.75(hexane)** |
| --- | --- | --- | --- |
| CCDC Number | 2388031 | 2388029 | 2388028 |
| Empirical formula | C_85_H_160_KN_2_O_6_Si_4_Sn_2_Y_2_ | C_85_H_163_Gd_2_KN_2_O_6_Si_4_Sn_2_ | C_86.5_H_166.5_Dy_2_KN_2_O_6_Si_4_Sn_2_ |
| Formula weight/g mol^–1^ | 1872.80 | 2012.50 | 2044.55 |
| Crystal system | monoclinic | monoclinic | monoclinic |
| Space group | *P*2_1_/*n* | *P*2_1_/*n* | *P*2_1_/*n* |
| *a*/Å | 13.6092(4) | 13.6621(6) | 13.6359(10) |
| *b*/Å | 34.3383(10) | 34.4140(14) | 34.391(3) |
| *c*/Å | 20.6055(7) | 20.4638(9) | 20.6138(15) |
| *α*/° | 90 | 90 | 90 |
| *β*/° | 95.008(2) | 94.968(2) | 94.950(3) |
| *γ*/° | 90 | 90 | 90 |
| *V*/Å^3^ | 9592.5(5) | 9585.3(7) | 9630.8(12) |
| *Z* | 4 | 4 | 4 |
| *ρ*_calc_/g cm^-3^ | 1.297 | 1.395 | 1.410 |
| *μ*/mm^‑1^ | 4.660 | 10.741 | 11.531 |
| *F*(000) | 3940.0 | 4152.0 | 4218.0 |
| Crystal size/mm^3^ | 0.08 × 0.06 × 0.04 | 0.15 × 0.1 × 0.05 | 0.07 × 0.05 × 0.03 |
| 2*θ* range for data collection/° | 4.364 to 109.91 | 4.382 to 114.154 | 4.36 to 109.856 |
| Index ranges | -16 ≤ h ≤ 16, -34 ≤ k ≤ 41, -25 ≤ l ≤ 24 | -17 ≤ h ≤ 17, -39 ≤ k ≤ 43, -25 ≤ l ≤ 25 | -16 ≤ h ≤ 16, -41 ≤ k ≤ 39, -25 ≤ l ≤ 25 |
| Reflections collected | 128557 | 146686 | 143447 |
| Independent reflections | 18231 [R_int_ = 0.1059, R_sigma_ = 0.0579] | 19573 [R_int_ = 0.0556, R_sigma_ = 0.0329] | 18270 [R_int_ = 0.0521, R_sigma_ = 0.0313] |
| Data/restraints/parameters | 18231/0/872 | 19573/114/872 | 18270/18/872 |
| Goodness-of-fit on *F*^2^ | 1.029 | 1.085 | 1.062 |
| Final *R* indexes [*I>=2σ(I)*] | R_1_ = 0.0495  wR_2_ = 0.1235 | R_1_ = 0.0552  wR_2_ = 0.1192 | R_1_ = 0.0404  wR_2_ = 0.0967 |
| Final *R* indexes [all data] | R_1_ = 0.0635  wR_2_ = 0.1311 | R_1_ = 0.0587  wR_2_ = 0.1207 | R_1_ = 0.0428  wR_2_ = 0.0978 |
| Largest diff. peak/hole / e Å^-3^ | 1.39/-1.37 | 2.10/-1.33 | 1.44/-1.43 |

**Table S3.** Crystal data for [K(2.2.2-crypt)]_2_[**3_Dy_**]·2(hexane), [K(2.2.2-crypt)]_2_[**3_Gd_**]·2(hexane), and [K(2.2.2-crypt)]_2_[**3_Y_**]·3(THF).

|  | **[K(2.2.2-crypt)]_2_[3_Dy_]·**  **2(hexane)** | **[K(2.2.2-crypt)]_2_[3_Gd_]·**  **2(hexane)** | **[K(2.2.2-crypt)]_2_[3_Y_]·**  **3(THF)** |
| --- | --- | --- | --- |
| CCDC Number | 2388033 | 2388034 | 2388036 |
| Empirical formula | C_53_H_103_DyKN_2_O_6_Si_2_Sn | C_53.03_H_100.03_GdKN_2_O_6_Si_2_Sn | C_59_H_113_KN_2_O_9_Si_2_SnY |
| Formula weight/g mol^–1^ | 1240.84 | 1233.83 | 1297.39 |
| Crystal system | triclinic | triclinic | triclinic |
| Space group | *P*$\bar{1}$ | *P*$\bar{1}$ | *P*$\bar{1}$ |
| *a*/Å | 13.3637(7) | 13.3951(6) | 13.4847(11) |
| *b*/Å | 14.2057(9) | 14.2387(7) | 16.3601(14) |
| *c*/Å | 17.5887(10) | 17.6344(8) | 16.7402(14) |
| *α*/° | 70.860(4) | 71.004(3) | 113.002(4) |
| *β*/° | 74.423(3) | 74.362(2) | 100.633(4) |
| *γ*/° | 89.384(4) | 89.229(3) | 92.040(4) |
| *V*/Å^3^ | 3027.6(3) | 3052.3(3) | 3317.3(5) |
| *Z* | 2 | 2 | 2 |
| *ρ*_calc_/g cm^-3^ | 1.361 | 1.342 | 1.299 |
| *μ*/mm^‑1^ | 9.463 | 8.750 | 3.695 |
| *F*(000) | 1292.0 | 1283.0 | 1378.0 |
| Crystal size/mm^3^ | 0.05 × 0.04 × 0.03 | 0.04 × 0.03 × 0.02 | 0.05 × 0.04 × 0.03 |
| 2*θ* range for data collection/° | 4.82 to 110.474 | 4.804 to 108.428 | 5.11 to 110.242 |
| Index ranges | -16 ≤ h ≤ 15, -17 ≤ k ≤ 17, -21 ≤ l ≤ 21 | -16 ≤ h ≤ 16, -17 ≤ k ≤ 17, -21 ≤ l ≤ 21 | -16 ≤ h ≤ 16, -19 ≤ k ≤ 19, -20 ≤ l ≤ 20 |
| Reflections collected | 53874 | 31568 | 59160 |
| Independent reflections | 11530 [R_int_ = 0.0807, R_sigma_ = 0.0668] | 11241 [R_int_ = 0.1133, R_sigma_ = 0.1318] | 12593 [Rint = 0.0665, Rsigma = 0.0495] |
| Data/restraints/parameters | 11530/0/558 | 11241/0/558 | 12593/0/559 |
| Goodness-of-fit on *F*^2^ | 1.016 | 1.071 | 1.127 |
| Final *R* indexes [*I>=2σ(I)*] | R_1_ = 0.0617  wR_2_ = 0.1414 | R_1_ = 0.0668  wR_2_ = 0.1534 | R1 = 0.0592  wR2 = 0.1705 |
| Final *R* indexes [all data] | R_1_ = 0.0839  wR_2_ = 0.1583 | R_1_ = 0.1143  wR_2_ = 0.1791 | R1 = 0.0676  wR2 = 0.1778 |
| Largest diff. peak/hole / e Å^-3^ | 1.54/-1.32 | 1.92/-1.18 | 1.78/-1.93 |

**Table S4.** Selected distances (Å) and angles (°) for **1_Y_**, **2_Y_,** and **3_Y_**.

|  | **1_Y_** | **2_Y_** | **3_Y_** |
| --- | --- | --- | --- |
| Y1–C (Cp^Sn^) | 2.629(4)-2.659(4)  av. 2.642 | 2.564(4)-2.624(4)  av. 2.586 | 2.435(4)-2.606(5)  av. 2.539 |
| Y1-Cp^Sn^(cent) | 2.3363(15) | 2.2666(18) | 2.202(2) |
| Y2–C (Cp^Sn^) | 2.630(4)-2.677(4)  av. 2.652 | 2.536(6)-2.647(6)  av. 2.586 |  |
| Y2-Cp^Sn^(cent) | 2.3467(16) | 2.262(2) |  |
| Y1-Sn1(η^5^) | 3.1541(6) | 3.1143(7) | 3.0618(7) |
| Y1-Sn2(η^1^) | 3.1760(6) | 3.1572(7) | 3.1345(5) |
| Y2-Sn1(η^1^) | 3.1588(6) | 3.1618(8) |  |
| Y2-Sn2(η^5^) | 3.1619(6) | 3.0935(8) |  |
| Y⋅⋅⋅Y | 5.3464(7) | 5.3542(8) | 5.3800(9) |
| Sn⋅⋅⋅Sn | 3.2080(4) | 3.1499(6) | 3.0749(7) |
| Sn1-C1 | 2.165(4) | 2.198(4) | 2.260(5) |
| C1-C2 | 1.422(4) | 1.452(6) | 1.457(6) |
| C2-C3 | 1.427(5) | 1.410(6) | 1.401(6) |
| C3-C4 | 1.426(5) | 1.449(6) | 1.453(7) |
| Sn1-C4 | 2.168(3) | 2.192(4) | 2.243(4) |
| Sn2-C13 | 2.162(4) | 2.219(5) |  |
| C13-C14 | 1.425(5) | 1.439(7) |  |
| C14-C15 | 1.429(5) | 1.400(7) |  |
| C15-C16 | 1.427(5) | 1.449(7) |  |
| Sn2-C16 | 2.172(4) | 2.183(5) |  |
| Y1–C (Cp^ttt^) | 2.628(4)-2.737(4)  av. 2.676 | 2.678(4)-2.765(4)  av. 2.717 | 2.670(5)-2.826(5)  av. 2.749 |
| Y1-Cp^ttt^(cent) | 2.3876(17) | 2.434(2) | 2.468(3) |
| Y2–C (Cp^ttt^) | 2.629(4)-2.719(4)  av. 2.673 | 2.654(4)-2.771(4)  av. 2.708 |  |
| Y2-Cp^ttt^(cent) | 2.3850(18) | 2.424(2) |  |
| Cp^ttt^-Y1-Cp^Sn^ | 146.39(5) | 147.13(7) | 146.79(7) |
| Cp^ttt^-Y2-Cp^Sn^ | 146.13(5) | 146.27(7) |  |

**Table S5.** Selected distances (Å) and angles (°) for **1_Gd_, 2_Gd_**, and **3_Gd_**.

|  | **1_Gd_** | **2_Gd_** | **3_Gd_** |
| --- | --- | --- | --- |
| Gd1–C (Cp^Sn^) | 2.666(2)-2.714(3)  av. 2.682 | 2.617(6)-2.654(6)  av. 2.631 | 2.470(11)-2.648(11)  av. 2.577 |
| Gd1-Cp^Sn^(cent) | 2.3821 (11) | 2.320(3) | 2.253 (4) |
| Gd2–C (Cp^Sn^) | 2.670(3)-2.731(3)  av. 2.692 | 2.580(7)-2.666(8)  av. 2.618 |  |
| Gd2-Cp^Sn^(cent) | 2.3911(11) | 2.300(3) |  |
| Gd1-Sn1(η^5^) | 3.1826(6) | 3.1480(7) | 3.1028 (7) |
| Gd1-Sn2(η^1^) | 3.1849(6) | 3.1649(6) | 3.1799(7) |
| Gd2-Sn1(η^1^) | 3.1676(5) | 3.1760(8) |  |
| Gd2-Sn2(η^5^) | 3.1882(5) | 3.1232(8) |  |
| Gd⋅⋅⋅Gd | 5.3506(7) | 5.3672(7) | 5.4658(9) |
| Sn⋅⋅⋅Sn | 3.2154(4) | 3.1780(6) | 3.0989(13) |
| Sn1-C1 | 2.157(2) | 2.195(6) | 2.263(8) |
| C1-C2 | 1.428(4) | 1.437(8) | 1.461(13) |
| C2-C3 | 1.416(4) | 1.409(8) | 1.399(12) |
| C3-C4 | 1.428(3) | 1.438(8) | 1.408(11) |
| Sn1-C4 | 2.160(3) | 2.185(5) | 2.228 (9) |
| Sn2-C13 | 2.159(3) | 2.186(7) |  |
| C13-C14 | 1.428(3) | 1.446(10) |  |
| C14-C15 | 1.425(4) | 1.399(10) |  |
| C15-C16 | 1.423(4) | 1.454(10) |  |
| Sn2-C16 | 2.162(2) | 2.197(7) |  |
| Gd1–C (Cp^ttt^) | 2.660(3)-2.766(3)  av. 2.711 | 2.712(6)-2.814(8)  av. 2.765 | 2.721(7)-2.834(10)  av. 2.788 |
| Gd1-Cp^ttt^(cent) | 2.4273(13) | 2.485(3) | 2.514(4) |
| Gd2–C (Cp^ttt^) | 2.666(3)-2.756(3)  av. 2.709 | 2.695(5)-2.813(7)  av. 2.746 |  |
| Gd2-Cp^ttt^(cent) | 2.4247(15) | 2.465(3) |  |
| Cp^ttt^-Gd1-Cp^Sn^ | 145.44(4) | 144.65(10) | 146.44(13) |
| Cp^ttt^-Gd2-Cp^Sn^ | 145.35(4) | 145.72(10) |  |

**Table S6.** Selected distances (Å) and angles (°) for **1_Dy_**, **2_Dy_**, and **3_Dy_**.

|  | **1_Dy_** | **2_Dy_** | **3_Dy_** |
| --- | --- | --- | --- |
| Dy1–C (Cp^Sn^) | 2.643(6)-2.679(6)  av. 2.656 | 2.569(4)-2.636(4)  av. 2.593 | 2.410(9)-2.619(8)  av. 2.529 |
| Dy1-Cp^Sn^(cent) | 2.354(3) | 2.2694(18) | 2.186(4) |
| Dy2–C (Cp^Sn^) | 2.642(6)-2.706(7)  av. 2.661 | 2.542(6)-2.659(6)  av. 2.589 |  |
| Dy2-Cp^Sn^(cent) | 2.361(3) | 2.262 (2) |  |
| Dy1-Sn1(η^5^) | 3.1659(6) | 3.1098(6) | 3.0590(6) |
| Dy1-Sn2(η^1^) | 3.1569(6) | 3.1571(7) | 3.1582(6) |
| Dy2-Sn1(η^1^) | 3.1489(6) | 3.1524(8) |  |
| Dy2-Sn2(η^5^) | 3.1691(6) | 3.0930(8) |  |
| Dy⋅⋅⋅Dy | 5.3459(7) | 5.3446(8) | 5.4151 (7) |
| Sn⋅⋅⋅Sn | 3.1881(5) | 3.1422(6) | 3.0560(12) |
| Sn1-C1 | 2.169(5) | 2.197(4) | 2.256(7) |
| C1-C2 | 1.431(7) | 1.461(6) | 1.459(9) |
| C2-C3 | 1.405(9) | 1.406(6) | 1.372(10) |
| C3-C4 | 1.415(7) | 1.454(6) | 1.505(10) |
| Sn1-C4 | 2.168(5) | 2.202(4) | 2.281(6) |
| Sn2-C13 | 2.155(6) | 2.218(5) |  |
| C13-C14 | 1.424(7) | 1.452(8) |  |
| C14-C15 | 1.405(9) | 1.412(8) |  |
| C15-C16 | 1.429(8) | 1.451(7) |  |
| Sn2-C16 | 2.160(5) | 2.187(5) |  |
| Dy1–C (Cp^ttt^) | 2.637(5)-2.732(6)  av. 2.682 | 2.691(4)-2.776(6)  av. 2.732 | 2.714(7)-2.825(10)  av. 2.774 |
| Dy1-Cp^ttt^(cent) | 2.395(3) | 2.450(2) | 2.496(3) |
| Dy2–C (Cp^ttt^) | 2.626(7)-2.729(7)  av. 2.677 | 2.669(4)-2.780(4)  av. 2.717 |  |
| Dy2-Cp^ttt^(cent) | 2.391(3) | 2.433(2) |  |
| Cp^ttt^-Dy1-Cp^Sn^ | 146.65(8) | 146.57(8) | 145.96(11) |
| Cp^ttt^-Dy2-Cp^Sn^ | 146.55(9) | 146.13(7) |  |

^^

**Figure S1.** Molecular structures of **1_Y_** and **1_Dy_** with thermal ellipsoid representation (50% probability). For clarity, hydrogen atoms are not shown.

^^

**Figure S2.** Molecular structures of [K(2.2.2-crypt)][**2_Y_**], [K(2.2.2-crypt)][**2_Gd_**] and [K(2.2.2-crypt)][**2_Dy_**] with thermal ellipsoid representation (50% probability). Hydrogen atoms are omitted for clarity.

^^

**Figure S3.** Molecular structures of [K(2.2.2-crypt)]_2_[**3_Y_**], [K(2.2.2-crypt)]_2_[**3_Gd_**] [K(2.2.2-crypt)]_2_[**3_Dy_**] with thermal ellipsoid representation (50% probability). Hydrogen atoms are omitted for clarity.

**FTIR Spectroscopy**

**
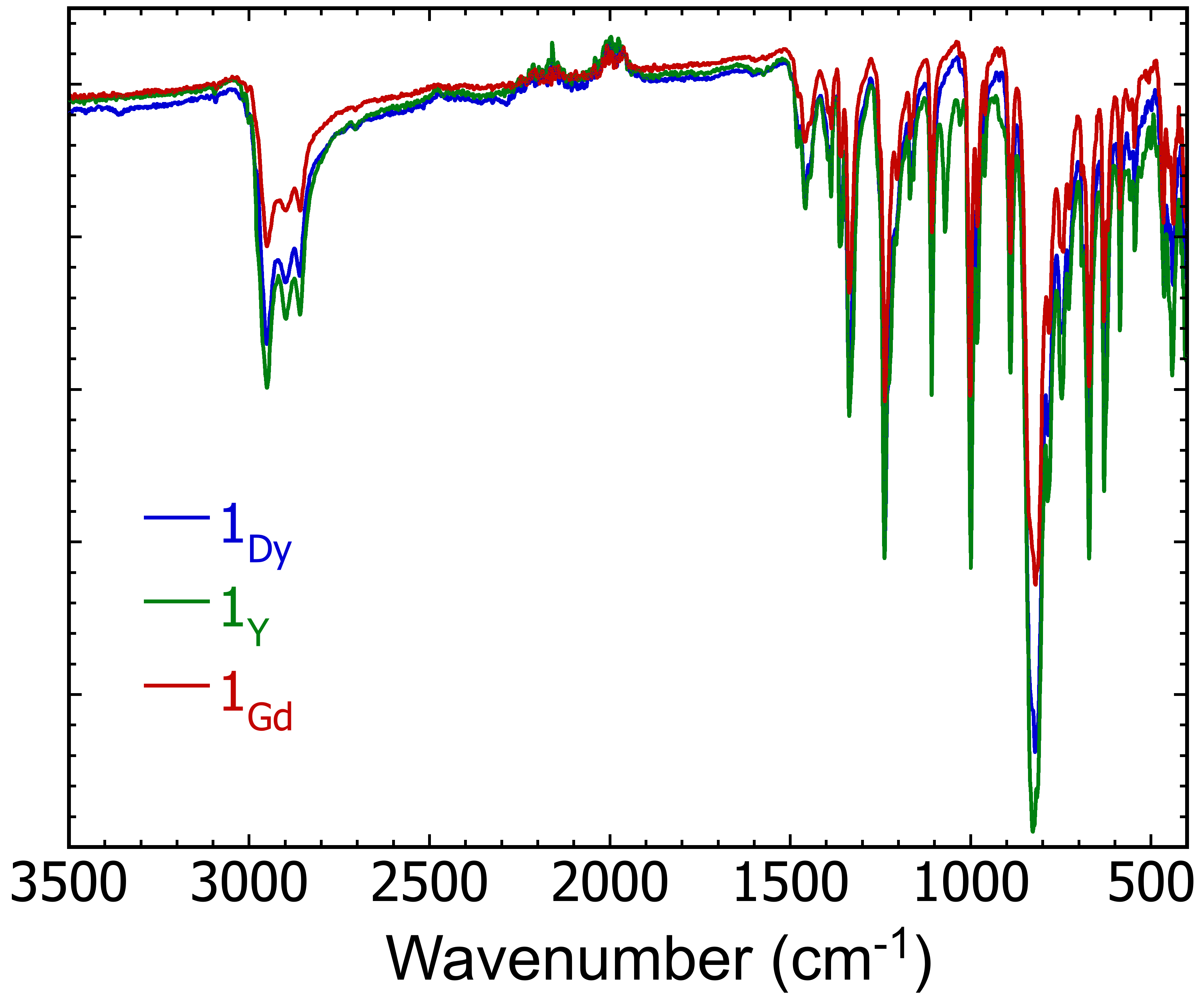
**

**Figure S4.** FTIR spectra of **1_Y_**·toluene (green), **1_Gd_**·toluene (red) and **1_Dy_**·toluene (blue).

**
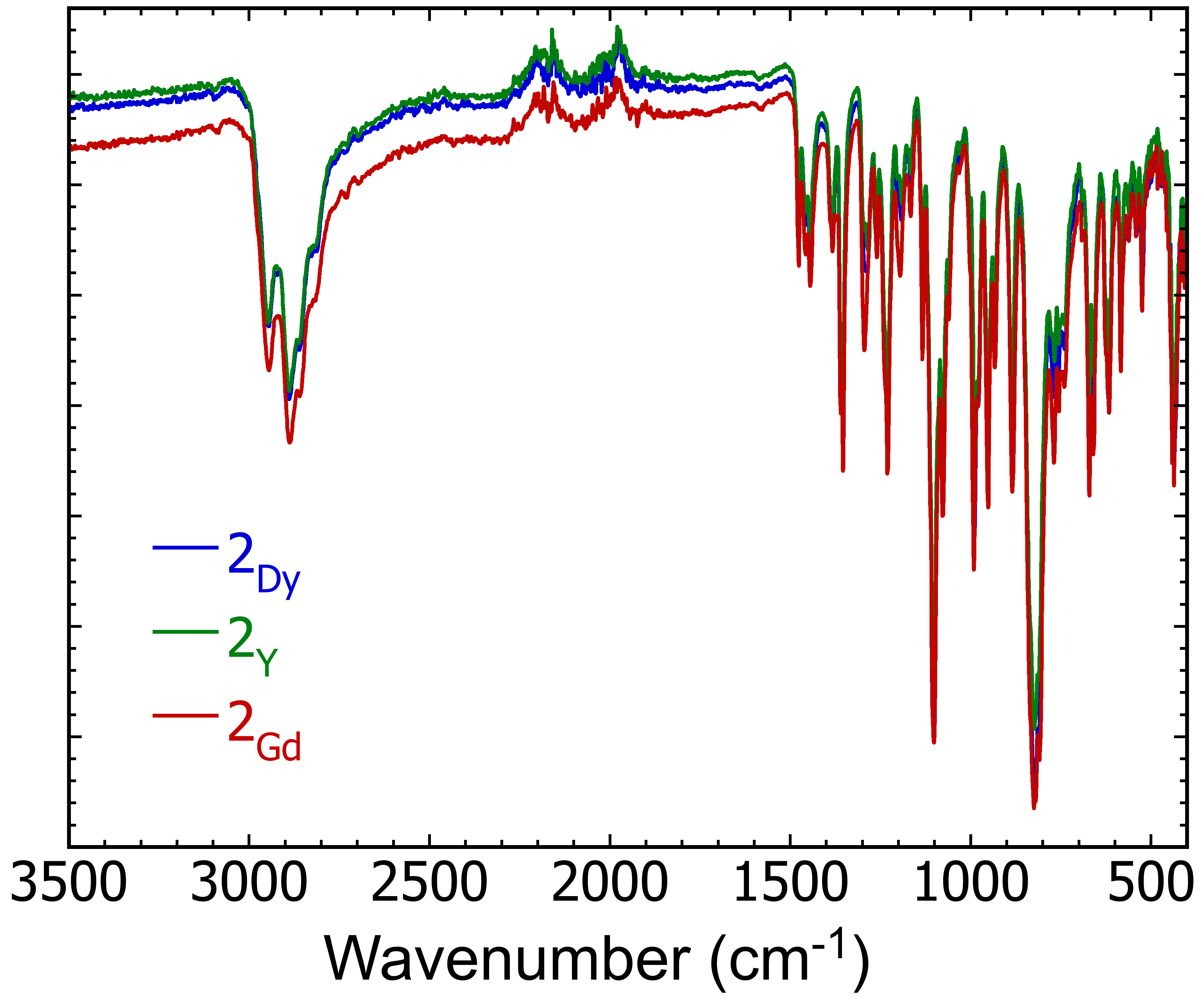
**

**Figure S5.** FTIR spectra of [K(2.2.2-crypt)][**2_Y_**]·1.5(hexane) (green), [K(2.2.2-crypt)][**2_Gd_**]·1.5(hexane) (red) and [K(2.2.2-crypt)][**2_Dy_**]·1.75(hexane) (blue).

**
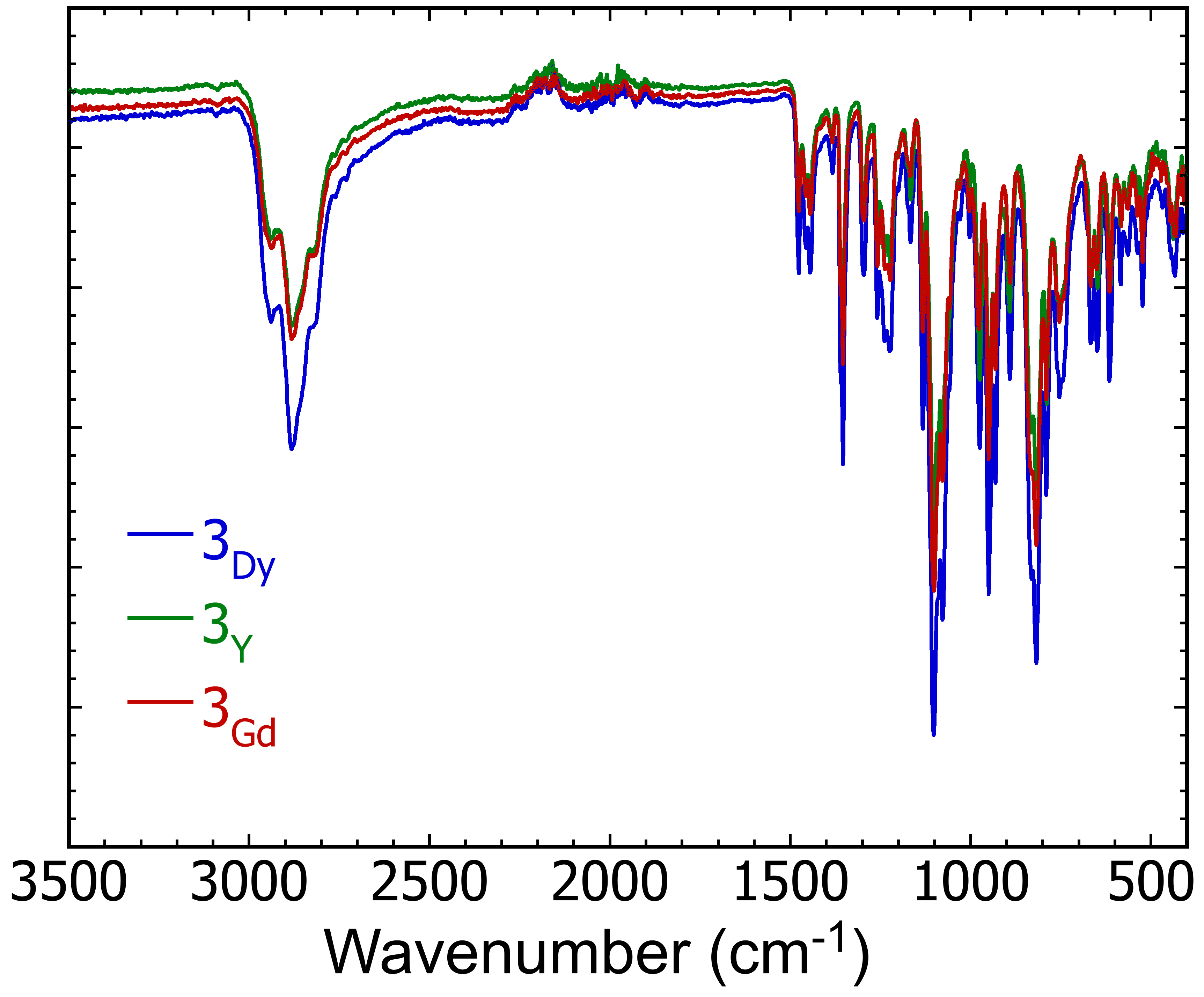
**

**Figure S6.** FTIR spectra of [K(2.2.2-crypt)]_2_[**3_Y_**]·3(THF) (green), [K(2.2.2-crypt)]_2_[**3_Gd_**]·2(hexane) (red), and [K(2.2.2-crypt)]_2_[**3_Dy_**]·2(hexane) (blue).

**NMR Spectroscopy**


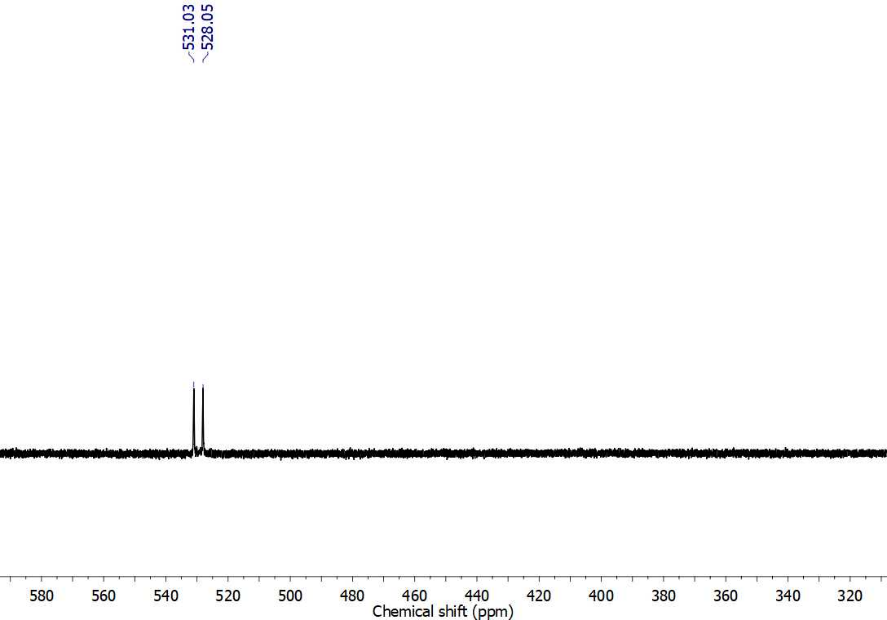


**Figure S7.** ^119^Sn{^1^H} NMR spectrum of **1_Y_**·toluene in toluene-D_8_ at 25°C.

**Figure S8.** ^1^H NMR spectrum of **1_Y_**·toluene in toluene-D_8_ at 25°C (* is residual protio solvent).


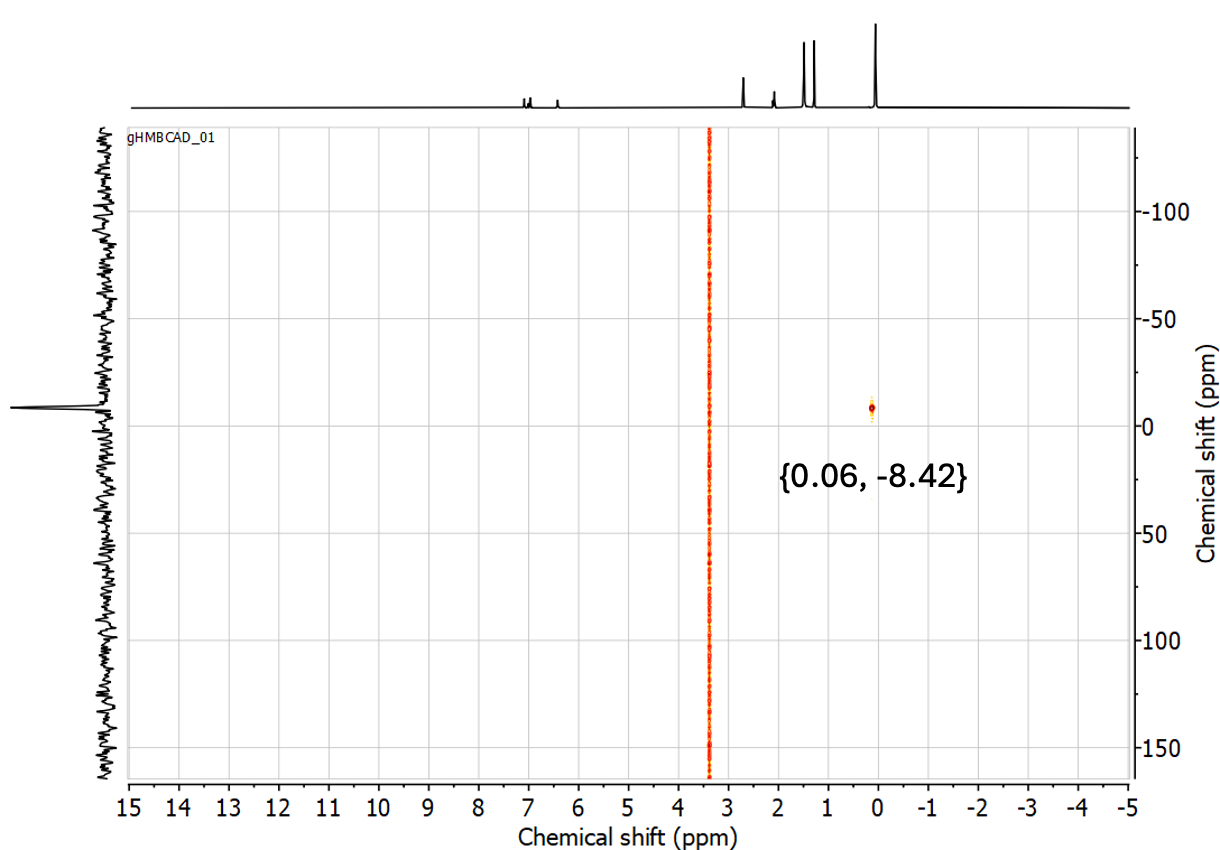


**Figure S9.** ^1^H/^29^Si HMBC NMR spectrum of **1_Y_**·toluene in toluene-D_8_ at 25°C.

**
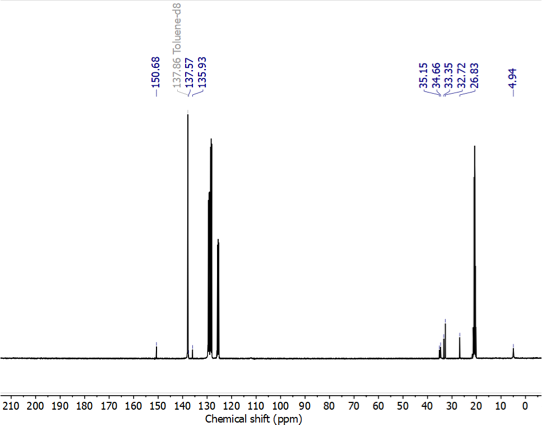
**

**Figure S10.** ^13^C NMR spectrum of **1_Y_** ·toluene in toluene-D_8_ at 25°C.

**Figure S11.** ^1^H NMR spectrum of [K(2.2.2-crypt)][**2_Y_**]·1.5(hexane) in THF-D_8_ at 25 °C (* is residual protio solvent).


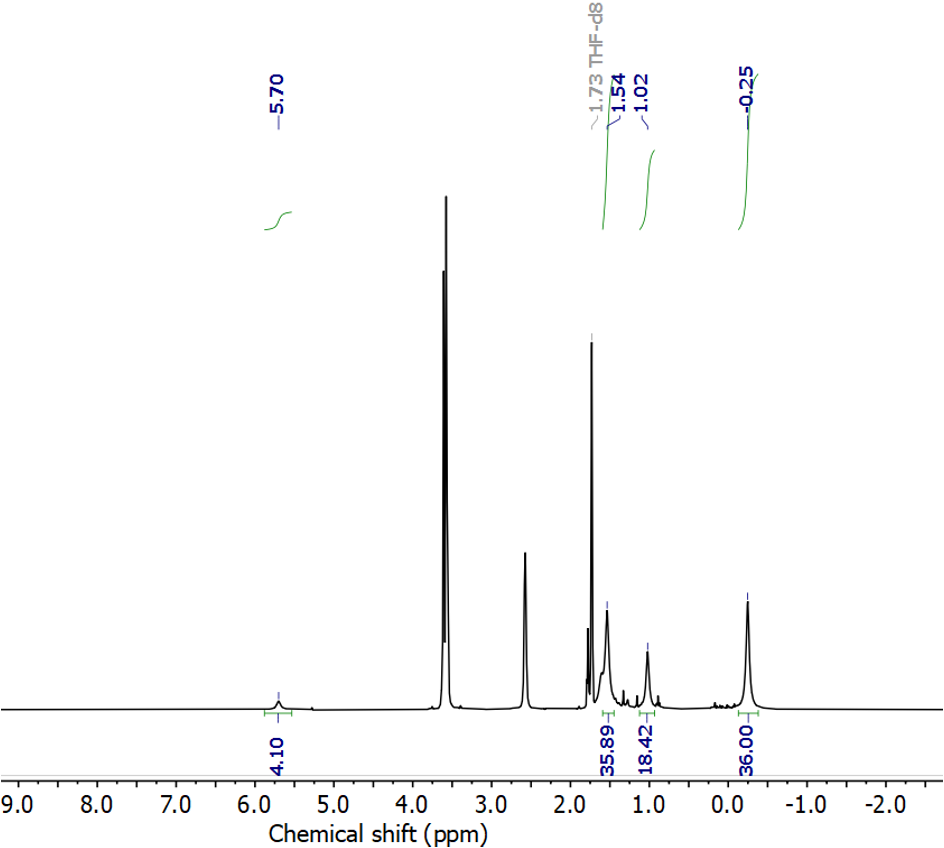


**Figure S12.** ^1^H NMR spectrum of [K(2.2.2-crypt)]_2_[**3_Y_**]·3(THF) in THF-D_8_ at 25°C (* is residual protio solvent).


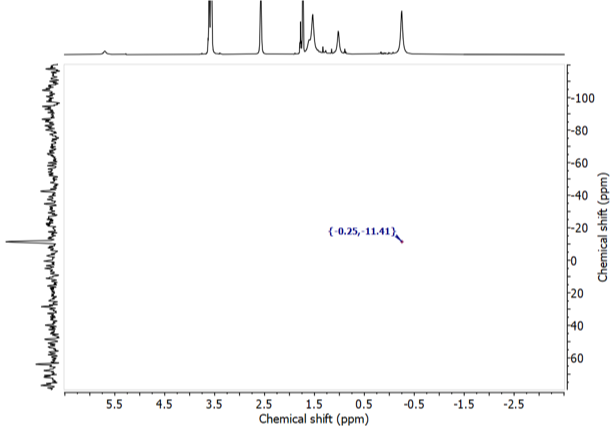


**Figure S13.** ^1^H/^29^Si HMBC-NMR spectrum of compound [K(2.2.2-crypt)]_2_[**3_Y_**]·3(THF) in THF-D_8_ at 25 °C.


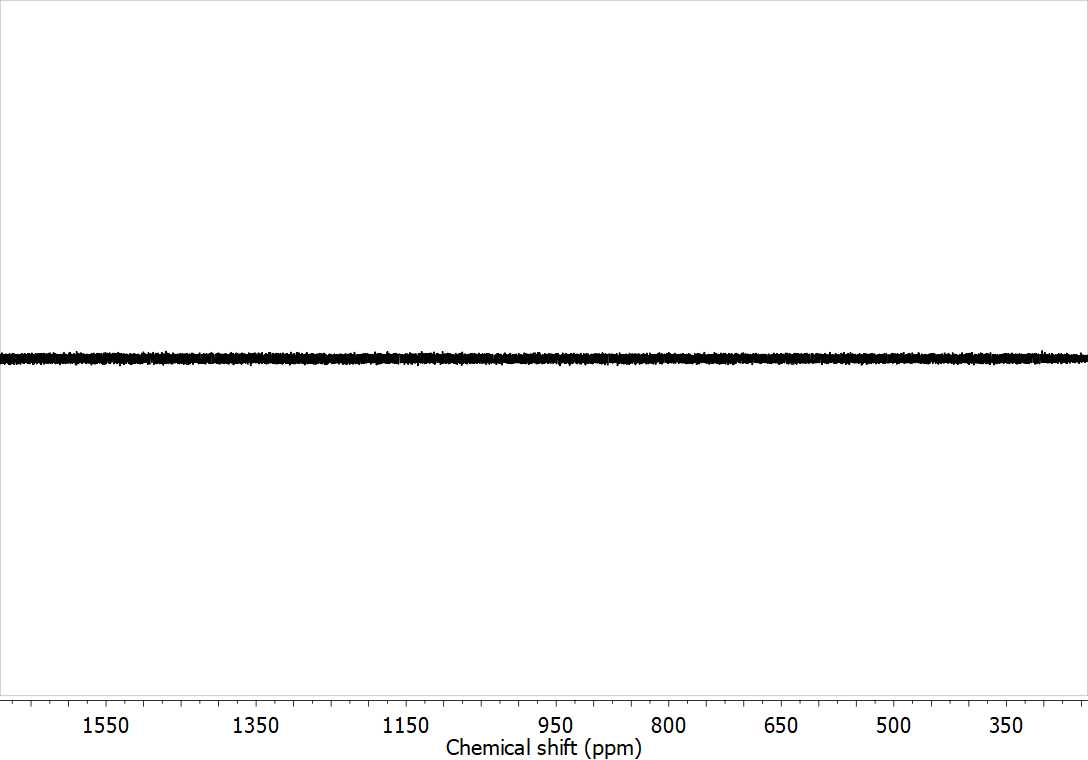


**Figure S14.** ^119^Sn{^1^H} NMR spectrum of compound [K(2.2.2-crypt)]_2_[**3_Y_**]·3(THF) in THF-D_8_ at 25 °C.

**Figure S15.** One-electron oxidation of [K(2.2.2-crypt)][**2_Y_**]·1.5(hexane) by AgPF_6_ to reform **1_Y_**. Lower: ^1^H NMR spectrum of [K(2.2.2-crypt)][**2_Y_**]·1.5(hexane). Middle: ^1^H NMR spectrum after adding one equivalent of AgPF_6_ to [K(2.2.2-crypt)][**2_Y_**]·1.5(hexane). Upper: ^1^H NMR spectrum of isolated **1_Y_**·toluene for comparison. Spectra were recorded in THF-D_8_ at 25°C (* is residual protio solvent).

**Figure S16.** One-electron reduction of [K(2.2.2-crypt)][**2_Y_**]·1.5(hexane) by KC_8_/2.2.2-crypt to form [K(2.2.2-crypt)]_2_[**3_Y_**]. Lower: ^1^H NMR spectrum of [K(2.2.2-crypt)][**2_Y_**]·1.5(hexane). Middle: ^1^H NMR spectrum after adding one equivalent of KC_8_/crypt to [K(2.2.2-crypt)][**2_Y_**]·1.5(hexane). Upper: ^1^H NMR spectrum of isolated [K(2.2.2-crypt)]_2_[**3_Y_**]·3(THF) for comparison. Spectra were recorded in THF-D_8_ at 25°C (* residual protio solvent).

**DFT Calculations**

Density functional theory (DFT) calculations were performed on the coordinates obtained from the X-ray structure using the ORCA 6.0.0 software package.^[104]^ Hydrogen atom positions were optimized at the DFT level using a pure GGA PBE exchange-correlation functional keeping the positions of the other atoms constant.^[105,106]^ The TPSSh functional was used^[107]^ and relativistic effects were included with the Douglas-Kroll-Hess Hamiltonian (DKH), together with the scalar relativistic contracted version of the basis functions def2-QZVP for Gd, def2-TZVP for Y, Sn, and def2-SVP for C and H atoms.^[108,109]^ The SARC/J auxiliary basis set^[110]^ and RIJCOSX approximation^[116,112]^ with TightSCF convergence threshold were also used throughout the calculations. Time-dependent DFT (TD-DFT) calculations were performed with 30 excited states and a CPCM implicit solvent model for THF.^[113,114]^ The Nucleus Independent Chemical Shift (NICS) calculations were performed with the whole molecule at the indicated position using b3lyp/def-TZVP/6-31g* level of theory in Gaussian16.^[115]^


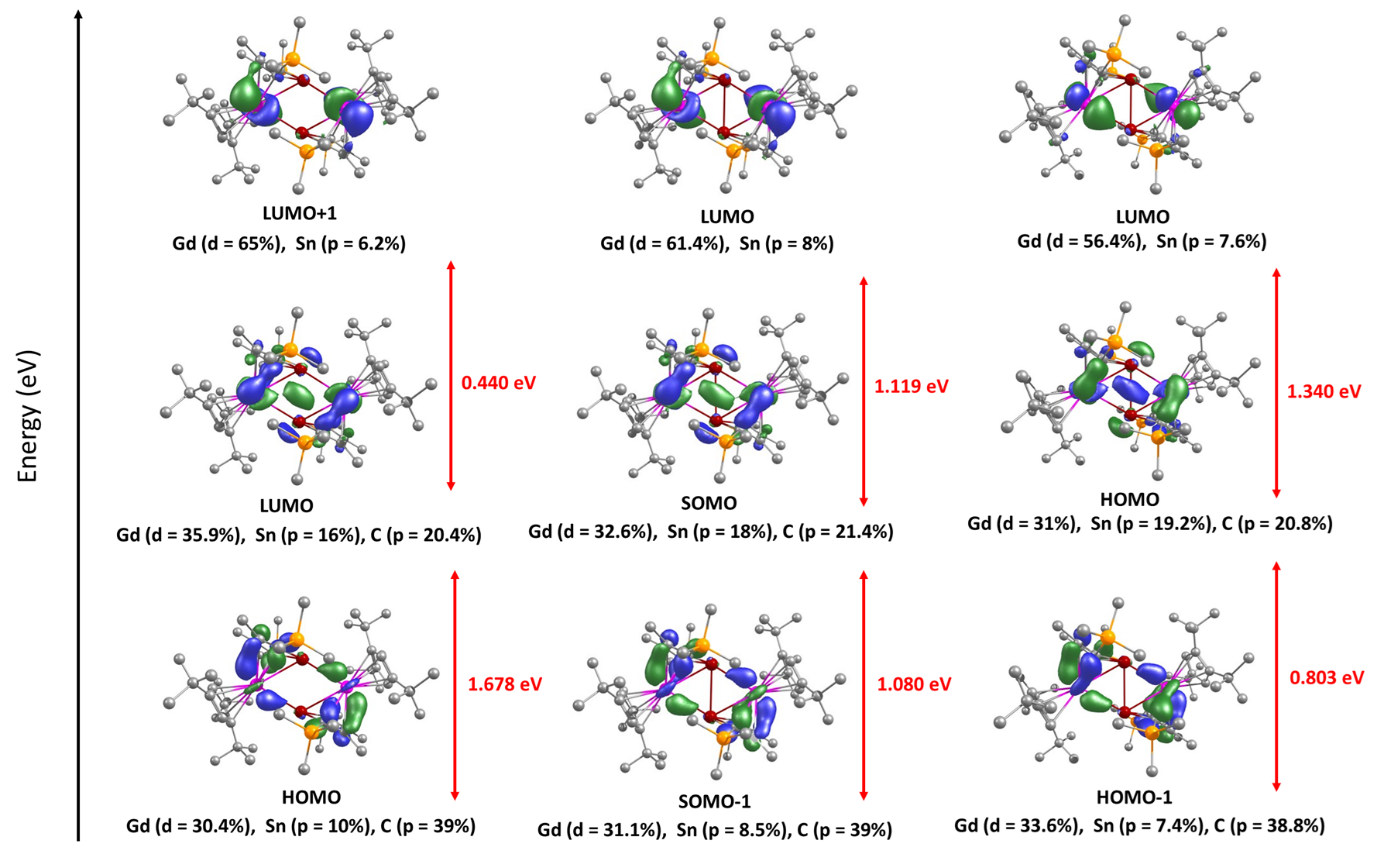


**Figure S17.** Frontier molecular orbitals for **1_Gd_** (left), the mono-anion **2_Gd_** (centre), and the di-anion **3_Gd_** (right). Isosurface value = 0.04 a.u.


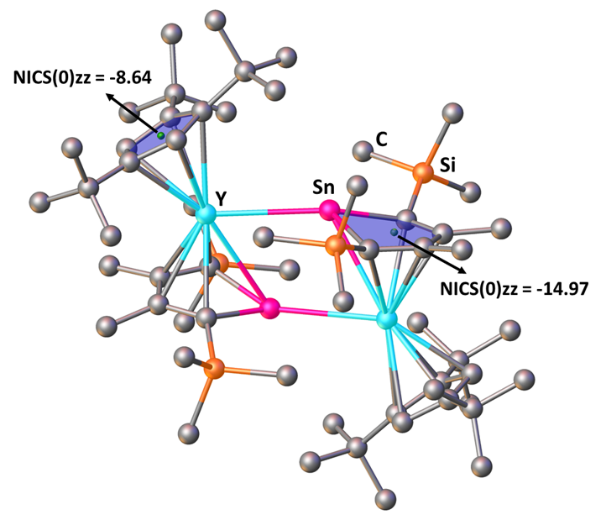

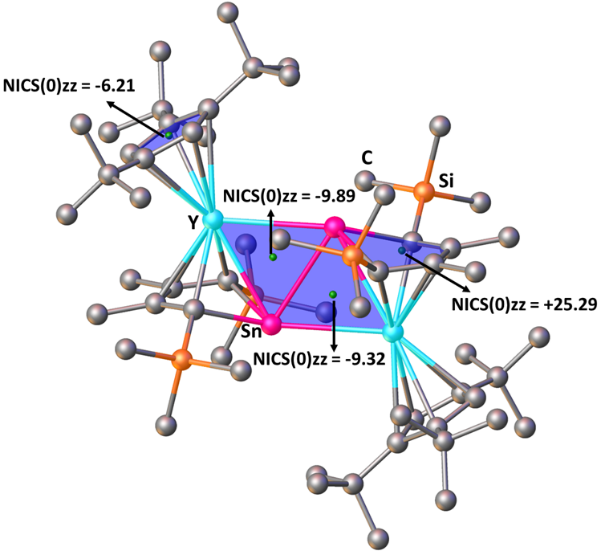


**Figure S18.** NICS calculations for **1_Y_** (left) and **3_Y_** (right).

**UV/vis Spectroscopy**


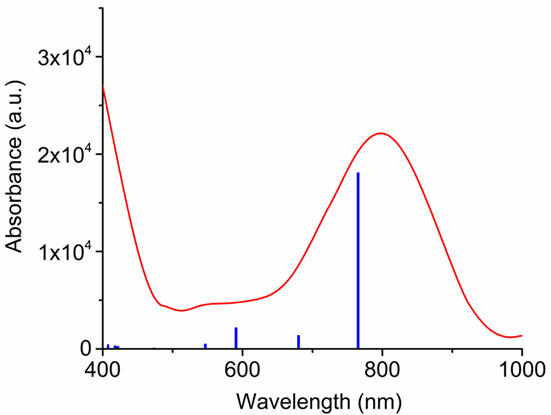

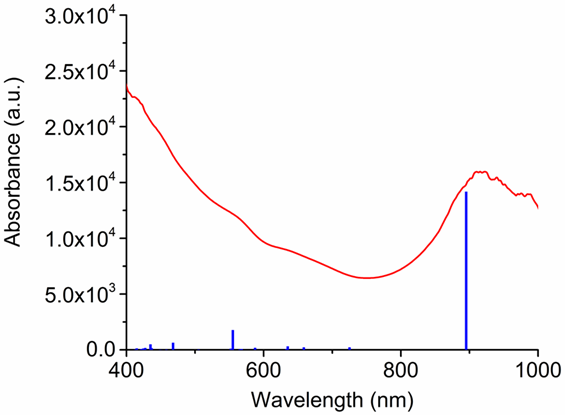

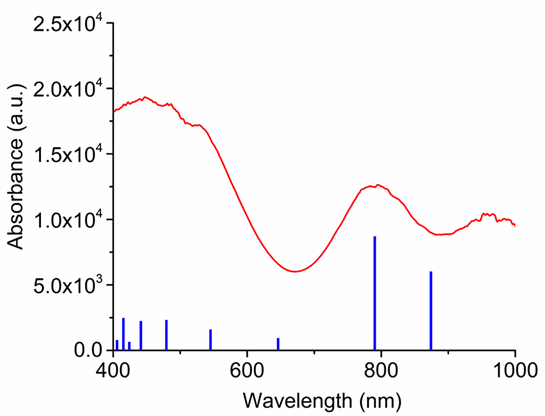


**Figure S19.** TD-DFT calculated UV/vis spectrum of **1_Y_** (left), **2_Y_** (centre) and **3_Y_** (right).

**Table S7.** Computed excitation wavelengths (*λ*) and oscillator strengths (*f*) in length representation for **1_Y_** (isosurface value = 0.04).

| **Excitation** | ***λ* / nm** | ***f*** | **Assignment (major contribution)** |
| --- | --- | --- | --- |
| 343a → 344a | 760 | 0.154 | 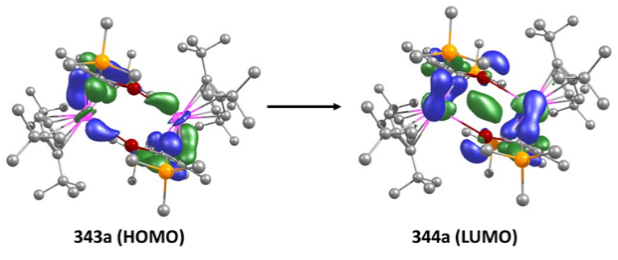 |
| 343a → 345a | 674 | 0.0113 | 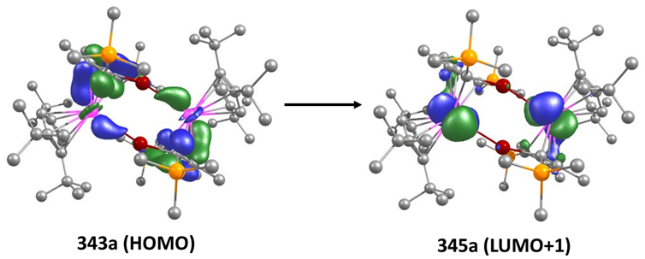 |
| 343a → 346a | 585 | 0.0182 | 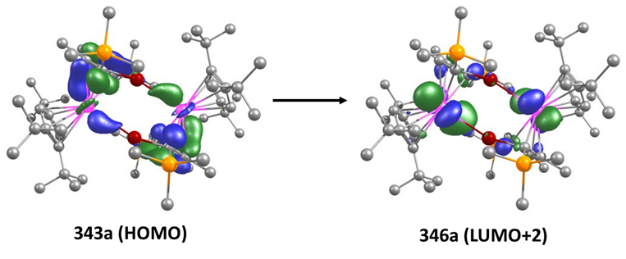 |
| 343a → 347a | 541 | 0.00377 | 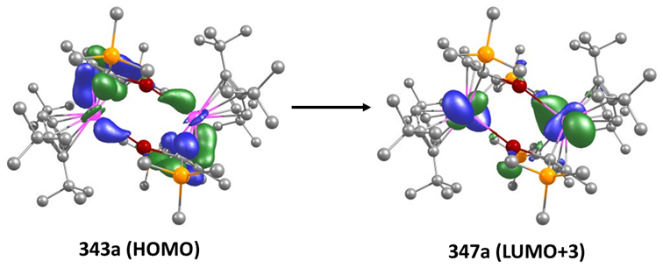 |
| 343a → 349a | 416 | 0.001722 | 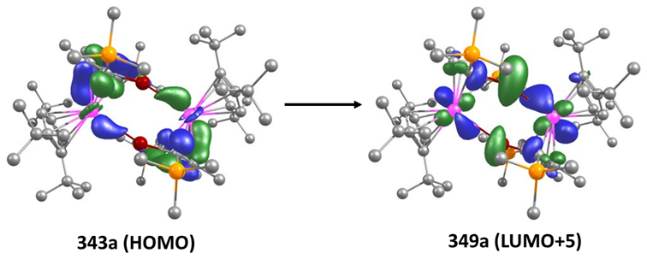 |
| 342a → 345a | 412 | 0.00220 | 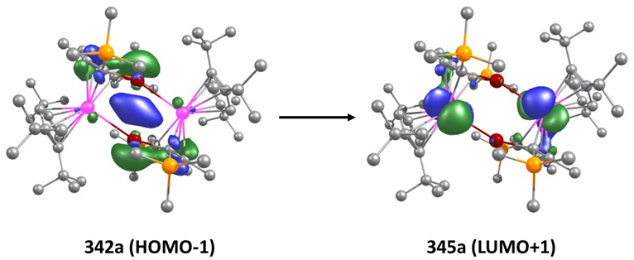 |
| 341a → 344a | 401 | 0.00317 | 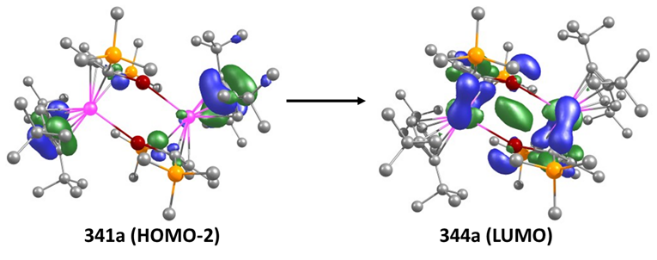 |

**Table S8.** Computed excitation wavelengths (*λ*) and oscillator strengths (*f*) in length representation for **2_Y_** (isosurface value = 0.04).

| **Excitation** | ***λ* / nm** | ***f*** | **Assignment (major contribution)** |
| --- | --- | --- | --- |
| 344a → 345a | 1436 | 0.00289 | 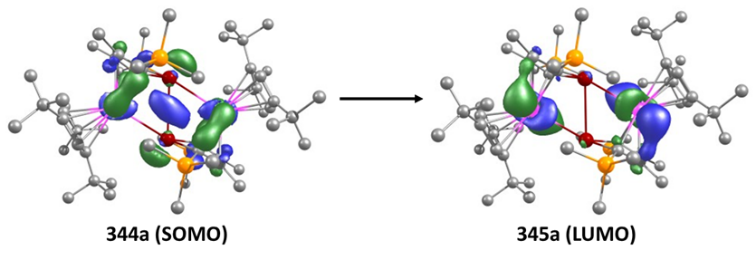 |
| 344a → 346a | 1168 | 0.01241 | 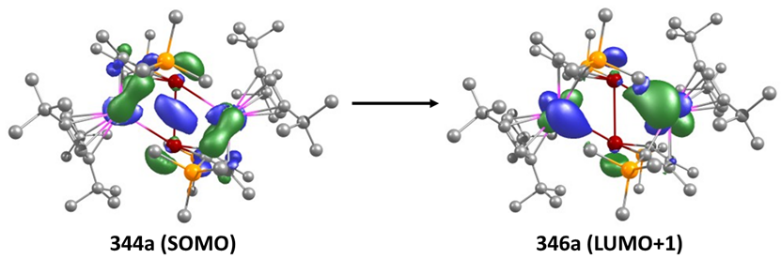 |
| 344a → 347a | 1098 | 0.02769 | 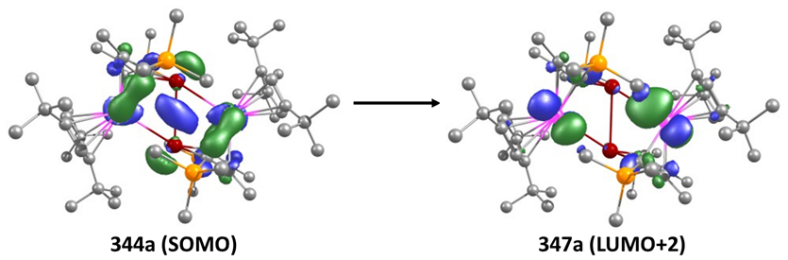 |
| 343b → 344b | 886 | 0.14188 | 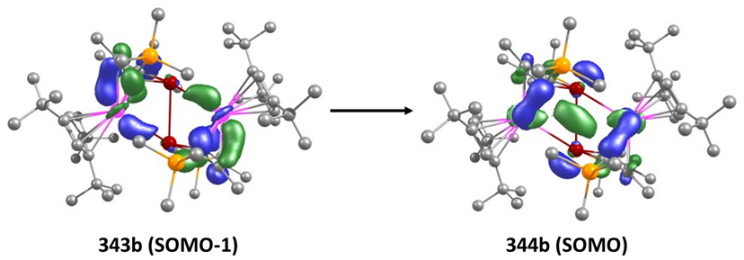 |
| 343b → 345b | 716 | 0.00242 | 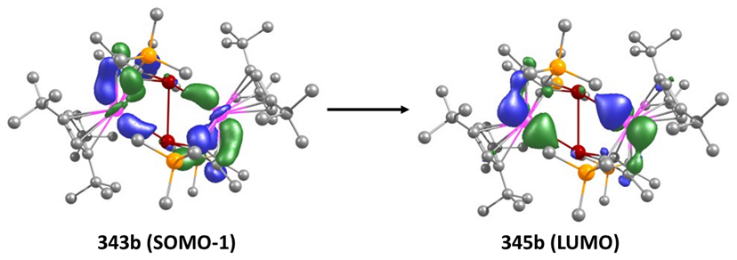 |
| 343a → 345a | 650 | 0.00244 | 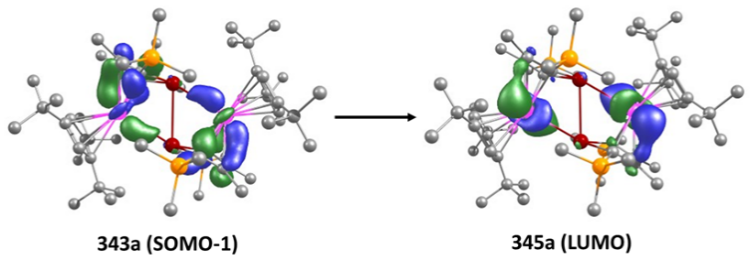 |
| 344a → 349a | 578 | 0.00194 | 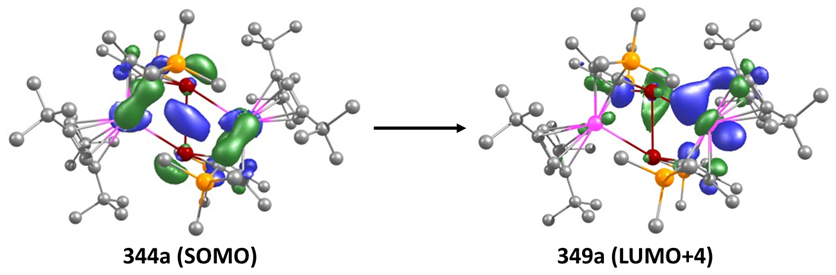 |
| 344a → 350a | 546 | 0.01795 | 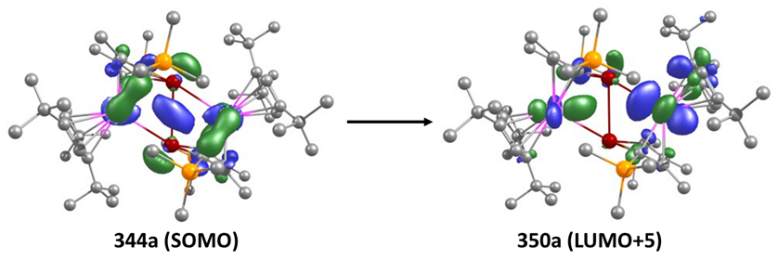 |
| 344a → 352a | 450 | 0.00656 | 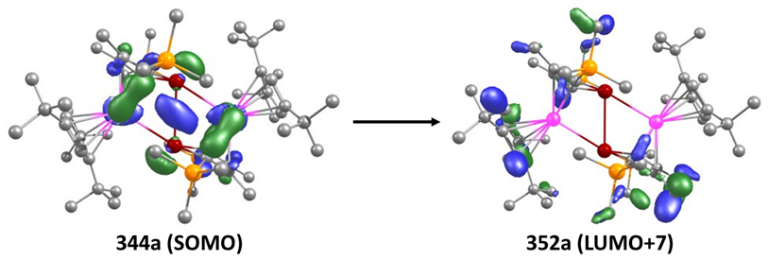 |

**Table S9.** Computed excitation wavelengths (*λ*) and oscillator strengths (*f*) in length representation for **3_Y_** (isosurface value = 0.04).

| **Excitation** | ***λ* / nm** | ***f*** | **Assignment (major contribution)** |
| --- | --- | --- | --- |
| 344a → 345a | 1066.3 | 0.00001 | **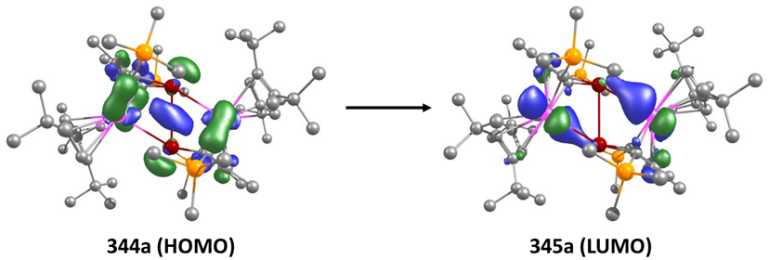** |
| 344a → 346a  344a → 347a  343a → 345a | 871 | 0.04818 | 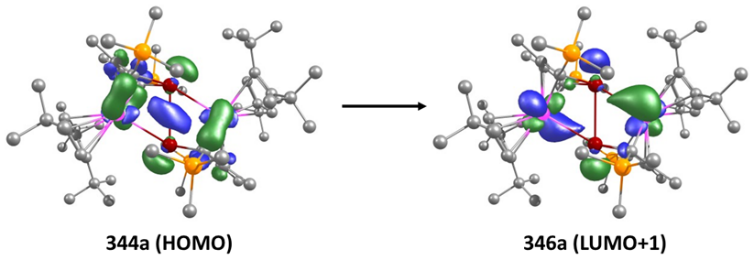  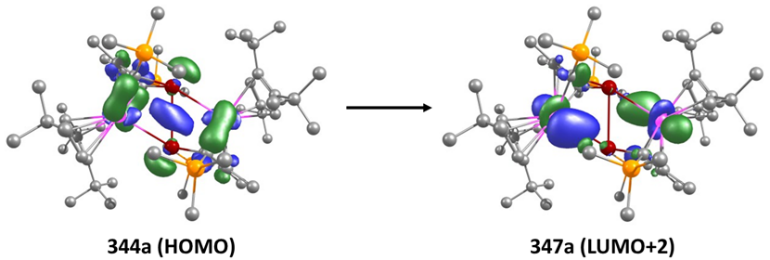  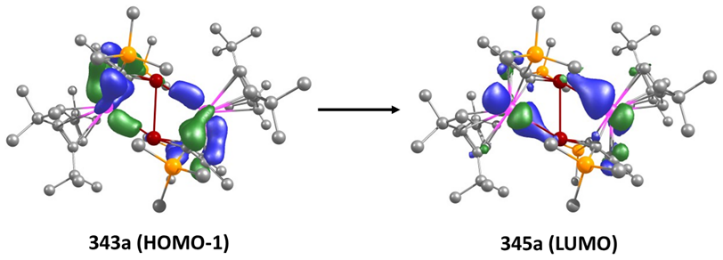 |
| 344a → 346a  344a → 347a | 788 | 0.06959 | 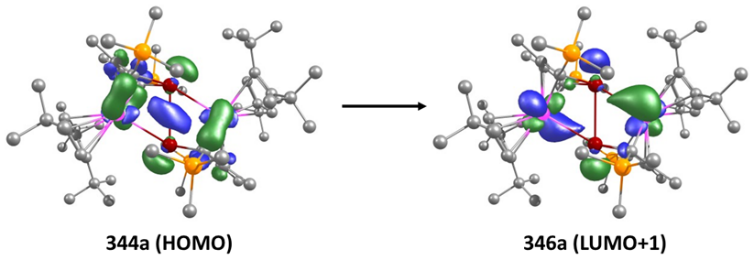  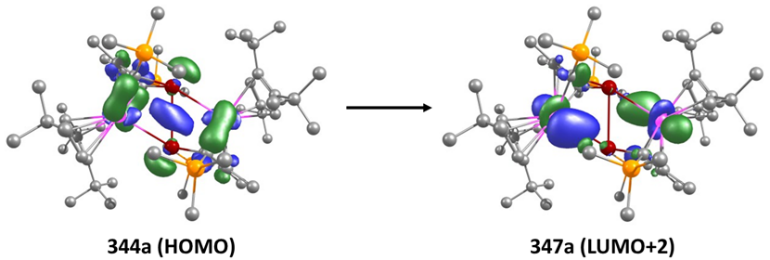 |
| 343a → 345a | 643 | 0.00749 | 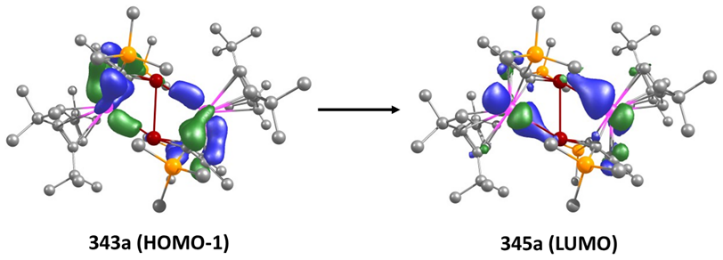 |
| 344a → 348a | 545 | 0.0127 | 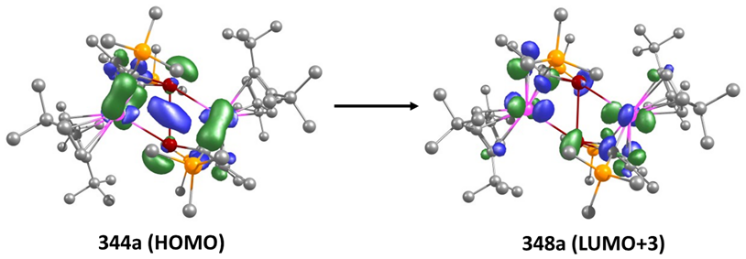 |
| 344a → 350a | 476 | 0.01855 | 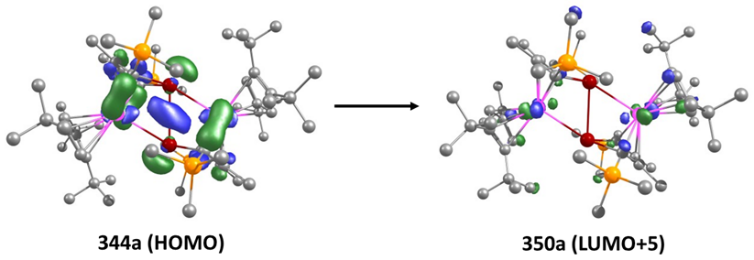 |
| 344a → 353a | 438 | 0.01793 | 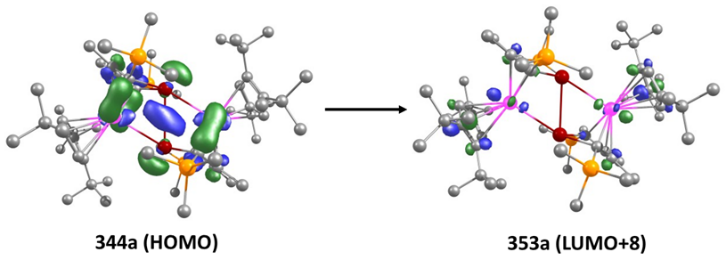 |
| 344a → 354a | 421 | 0.005168 | 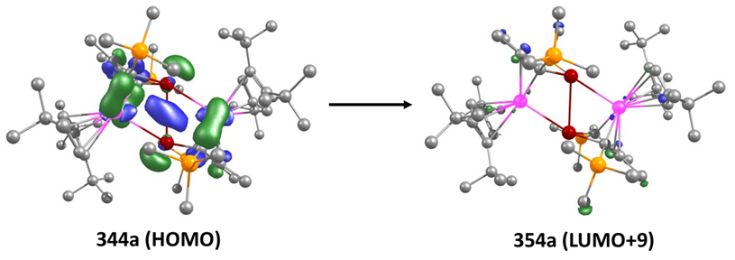 |
| 344a → 356a | 413 | 0.019741 | 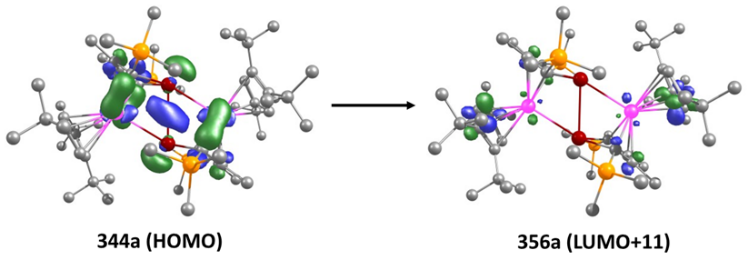 |
| 342a → 346a  344a → 356a | 405 | 0.00633 | 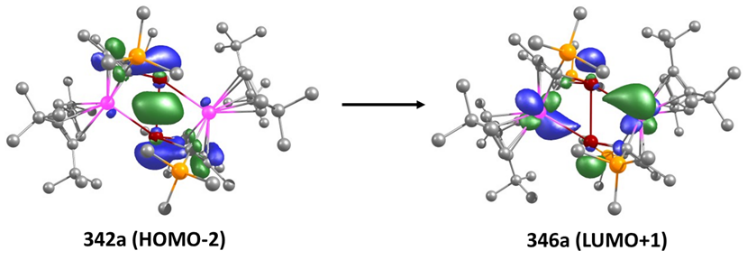  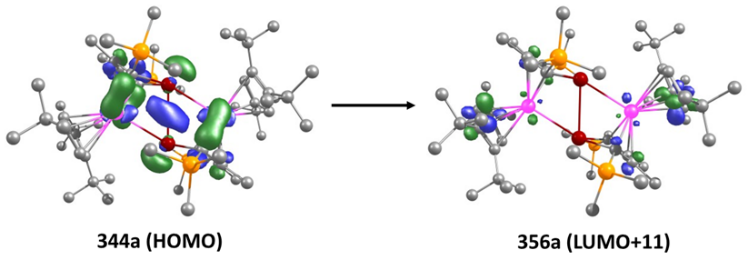 |

**
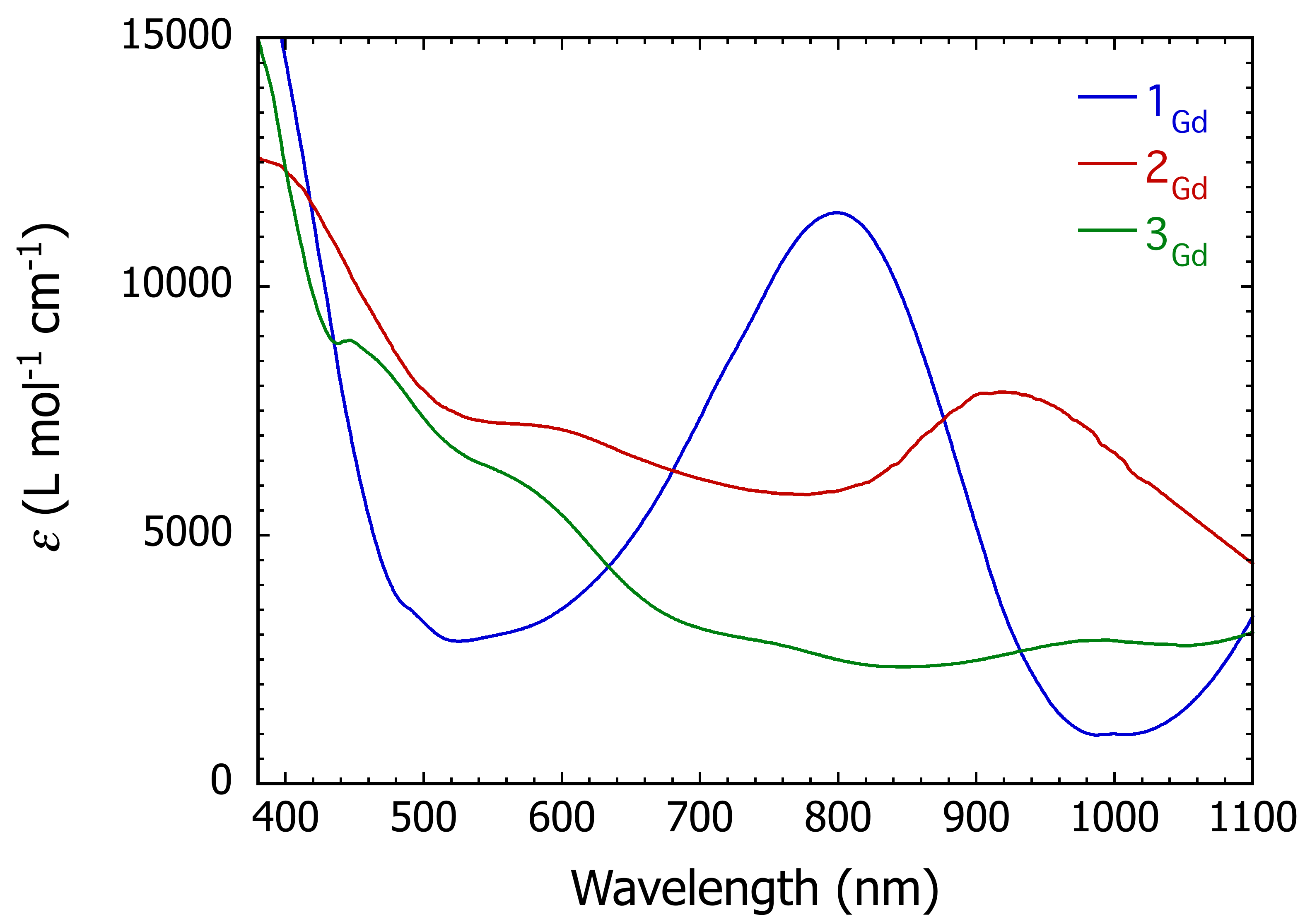
**

**Figure S20.** UV/vis spectra of **1_Gd_**·toluene (blue), [K(2.2.2-crypt)][**2_Gd_**]·1.5(hexane) (red), and [K(2.2.2-crypt)]_2_[**3_Gd_**]·2(hexane) (green) in THF.

**
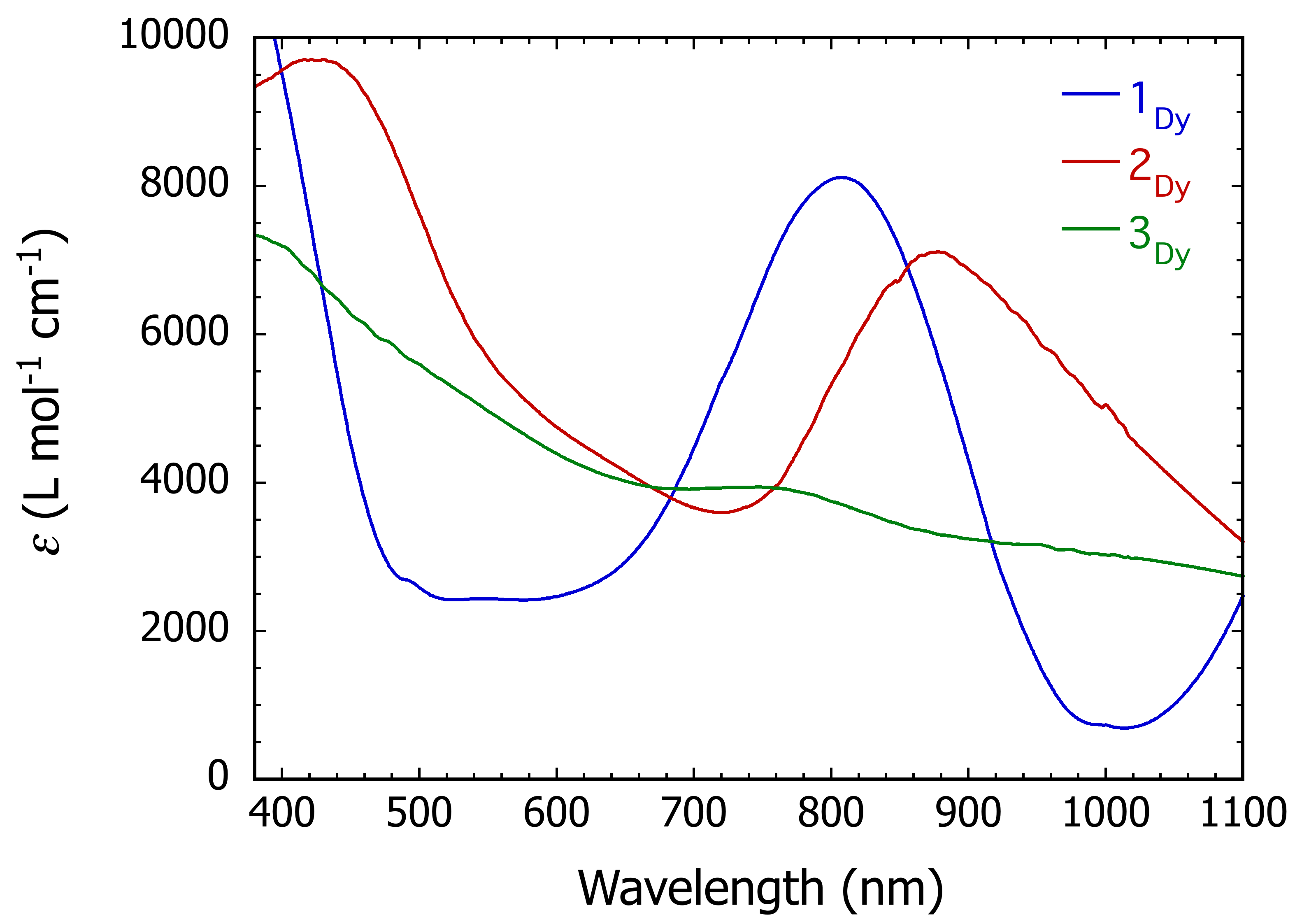
**

**Figure S21.** UV/vis absorbance spectra of **1_Dy_**·toluene (blue), [K(2.2.2-crypt)][**2_Dy_**]·1.75(hexane) (red), and [K(2.2.2-crypt)]_2_[**3_Dy_**]·2(hexane) (green) in THF.

**Magnetic Measurements**

Magnetic measurements were carried out on a Quantum Design MPMS-XL7 SQUID magnetometer equipped with a 7 T magnet. Samples were prepared by gently grinding the crystalline materials before transferring them to a 7 mm NMR tube and restraining them in eicosane. The tubes were flame-sealed under vacuum. Direct current (DC) magnetic susceptibility measurements were performed on the polycrystalline sample in the temperature range 1.9-300 K in an applied field of 1000 Oe. Alternating current (AC) susceptibility measurements were performed using an AC field of 3 Oe in zero DC field. Diamagnetic corrections were performed using Pascal’s constants.^[116]^


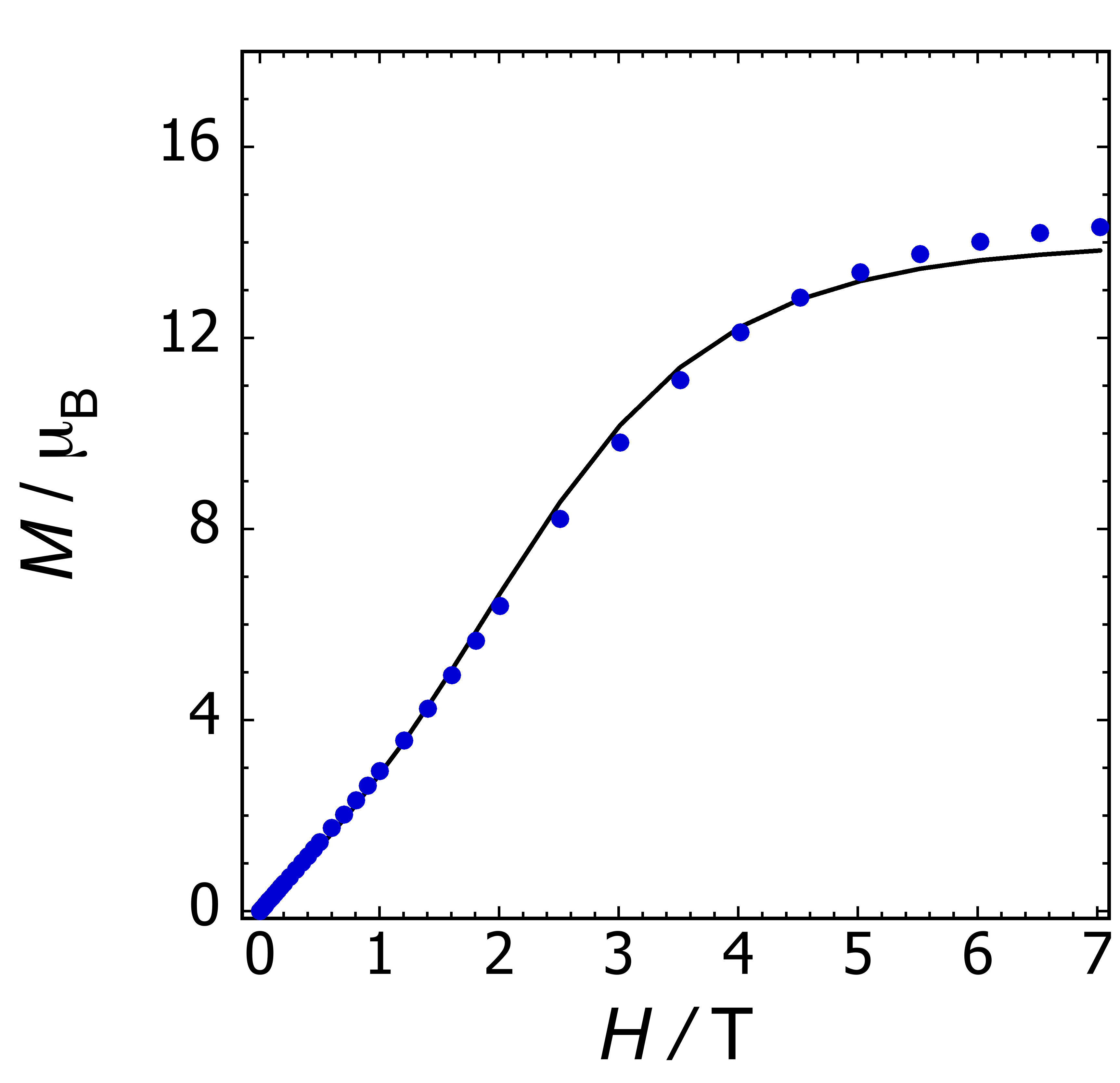

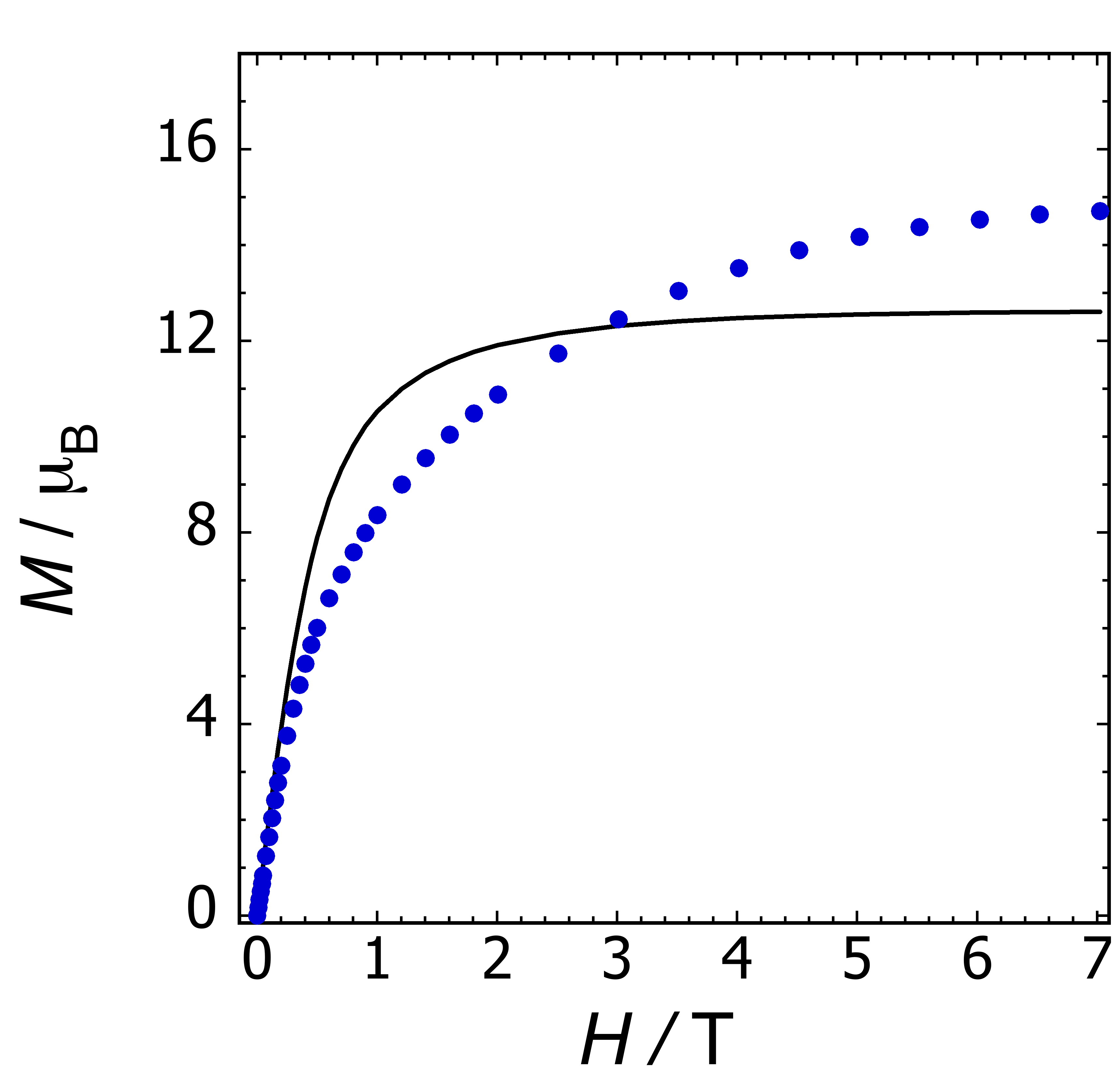

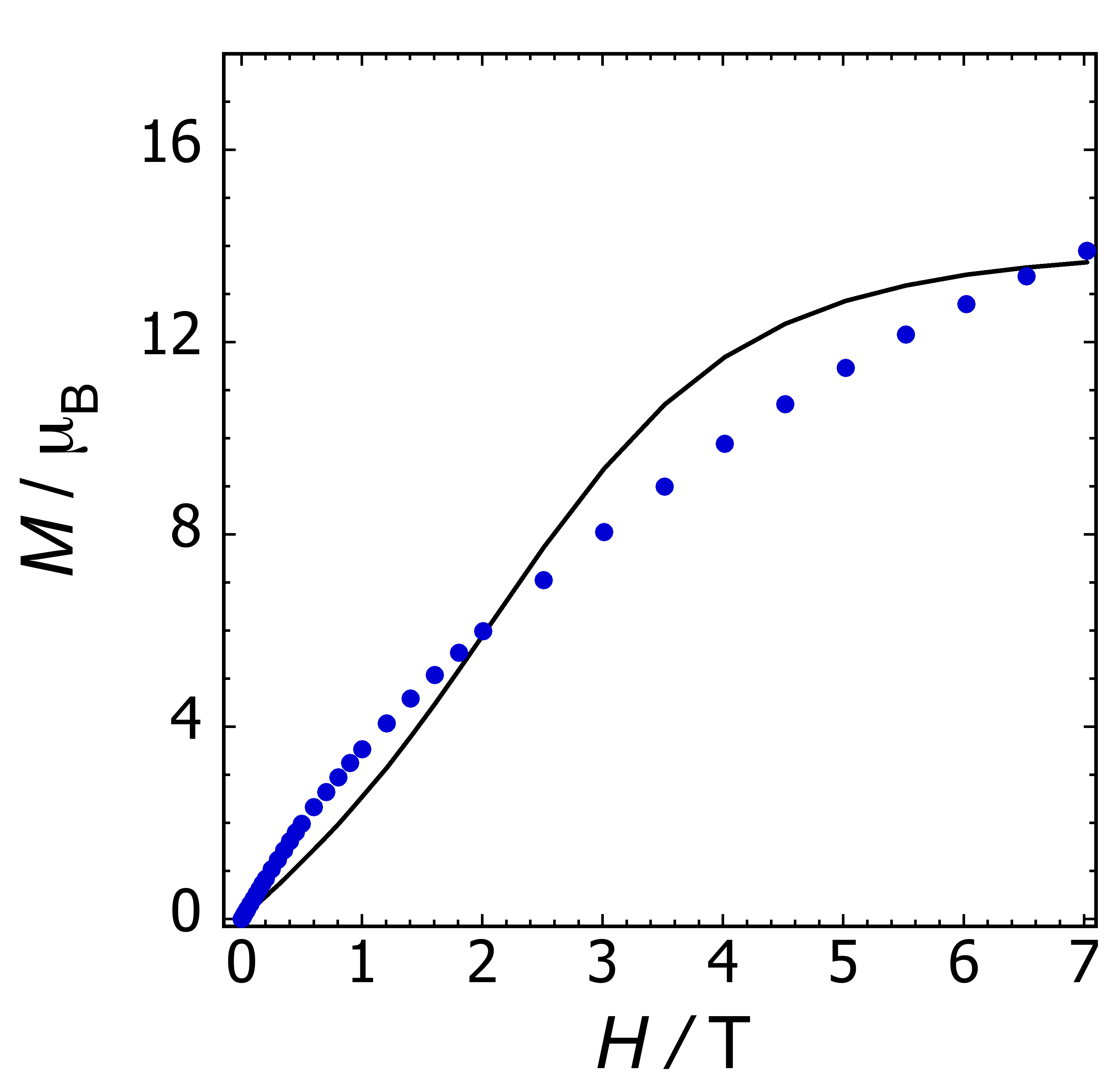


**Figure S22.** Isothermal field dependence of magnetization for **1_Gd_**·toluene (left), [K(2.2.2-crypt)][**2_Gd_**]·1.5(hexane) (middle), and [K(2.2.2-crypt)]_2_[**3_Gd_**]·2(hexane) (right) at 2 K. The magnetization values at 7 T are 14.31, 14.70, and 13.89 μ_B_, respectively. Solid black lines are simulations using the parameters stated in the main text.

**
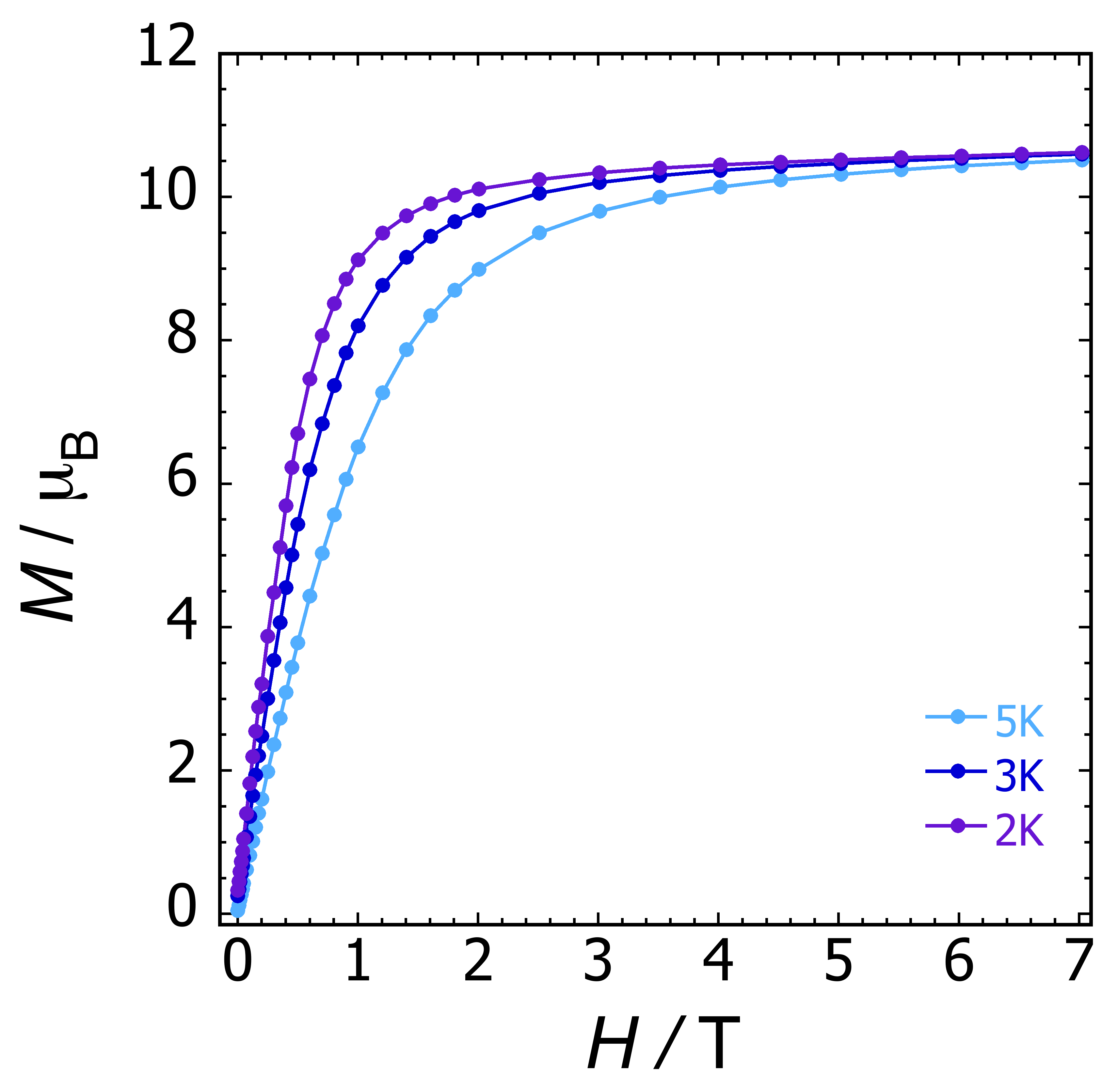

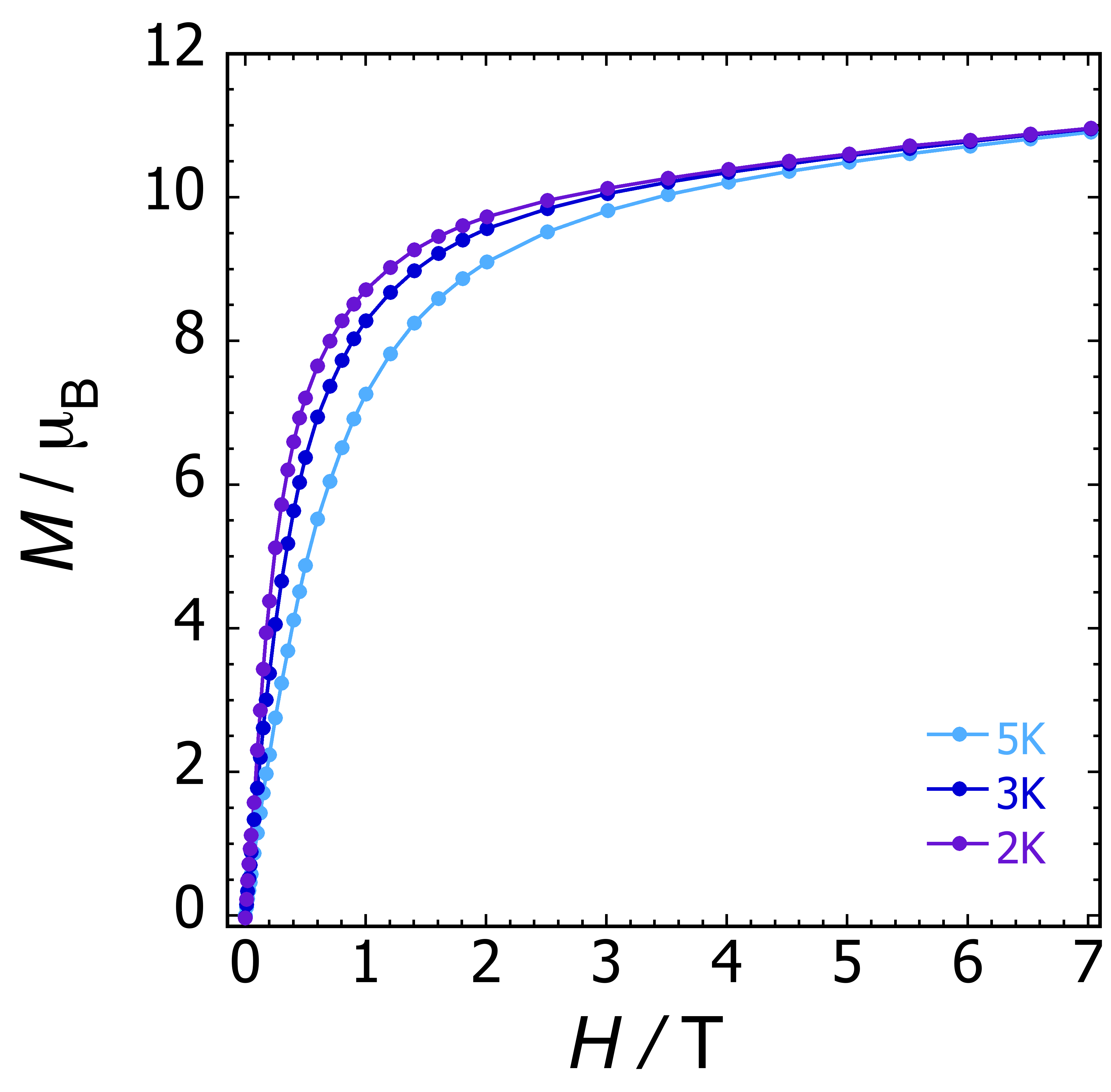

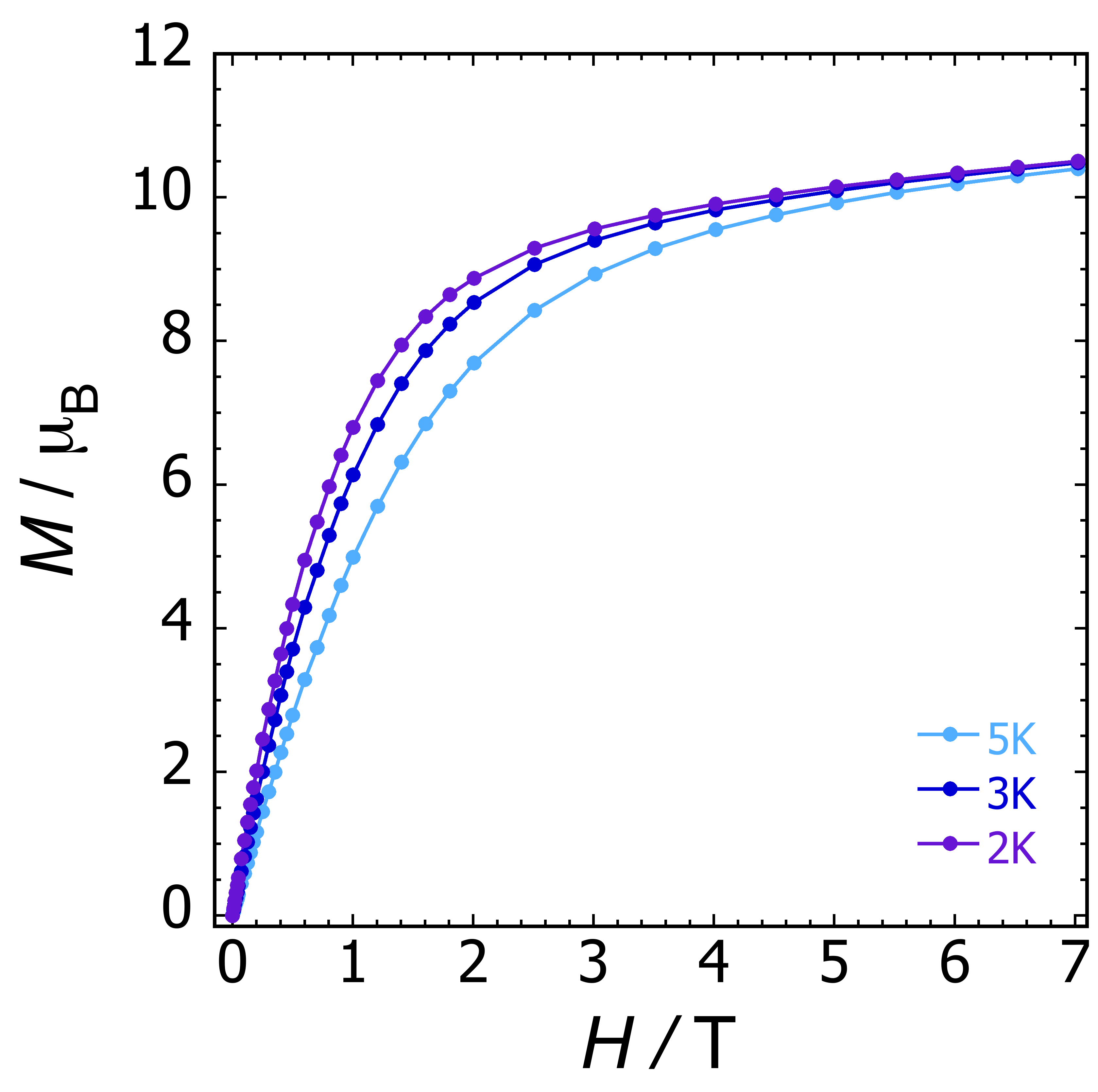
**

**Figure S23.** Isothermal field dependence of magnetization for **1_Dy_**·toluene (left), [K(2.2.2-crypt)][**2_Dy_**]·1.75(hexane) (middle), and [K(2.2.2-crypt)]_2_[**3_Dy_**]·2(hexane) (right) at 2 K, 3 K and 5 K. The magnetization values at 7 T are 10.6 μ_B_, 10.96 μ_B_, and 10.5 μ_B_, respectively. Solid lines are a guide for the eye.

**
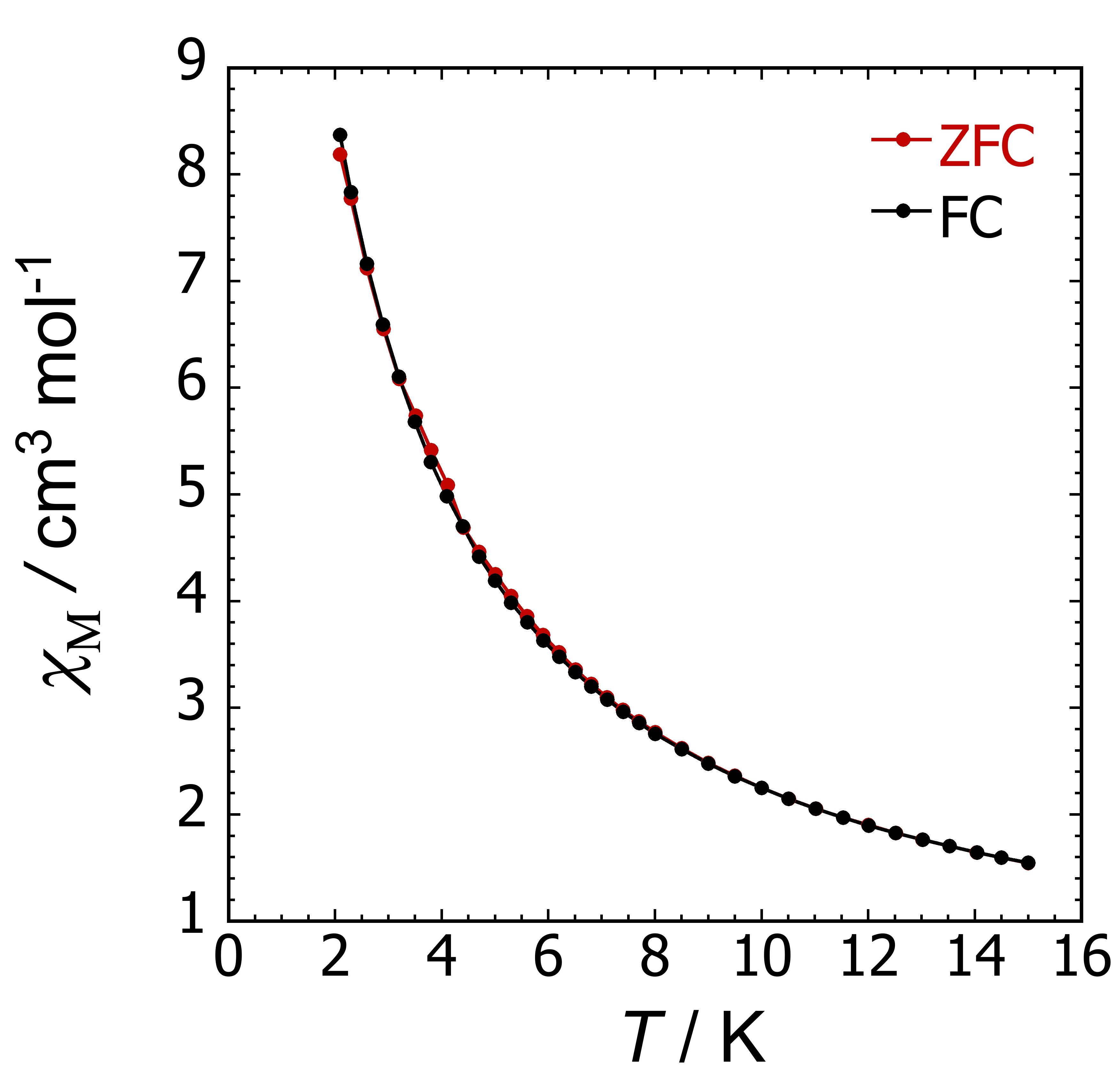

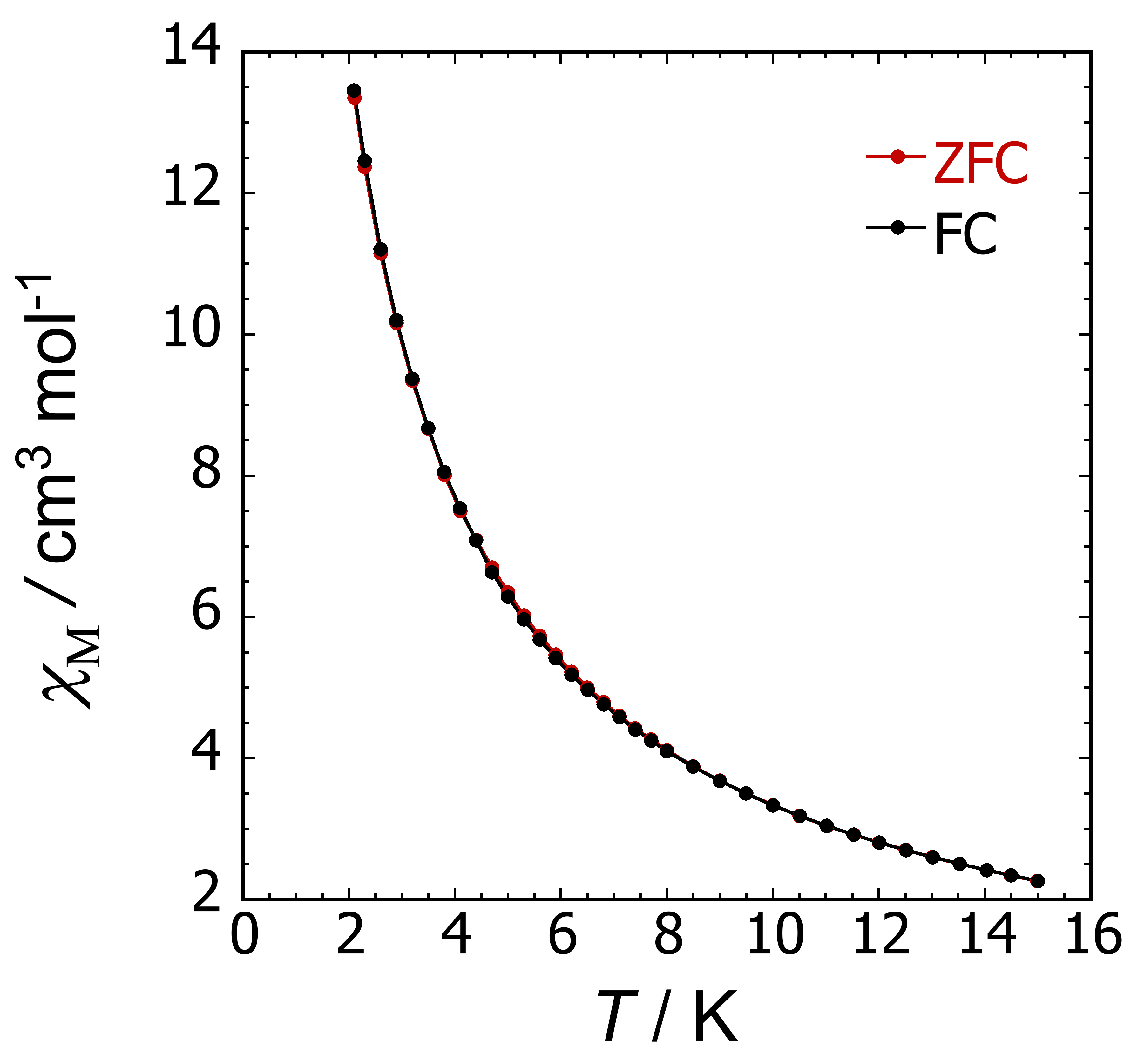
**

**Figure S24.** Zero-field-cooled (ZFC) and field-cooled (FC) (*H*_DC_ = 1000 Oe) magnetic susceptibility versus temperature for **1_Dy_**·toluene (left), and [K(2.2.2-crypt)][**2_Dy_**]·1.75(hexane) (right). Average scan rate = 0.22 K min^–1^.


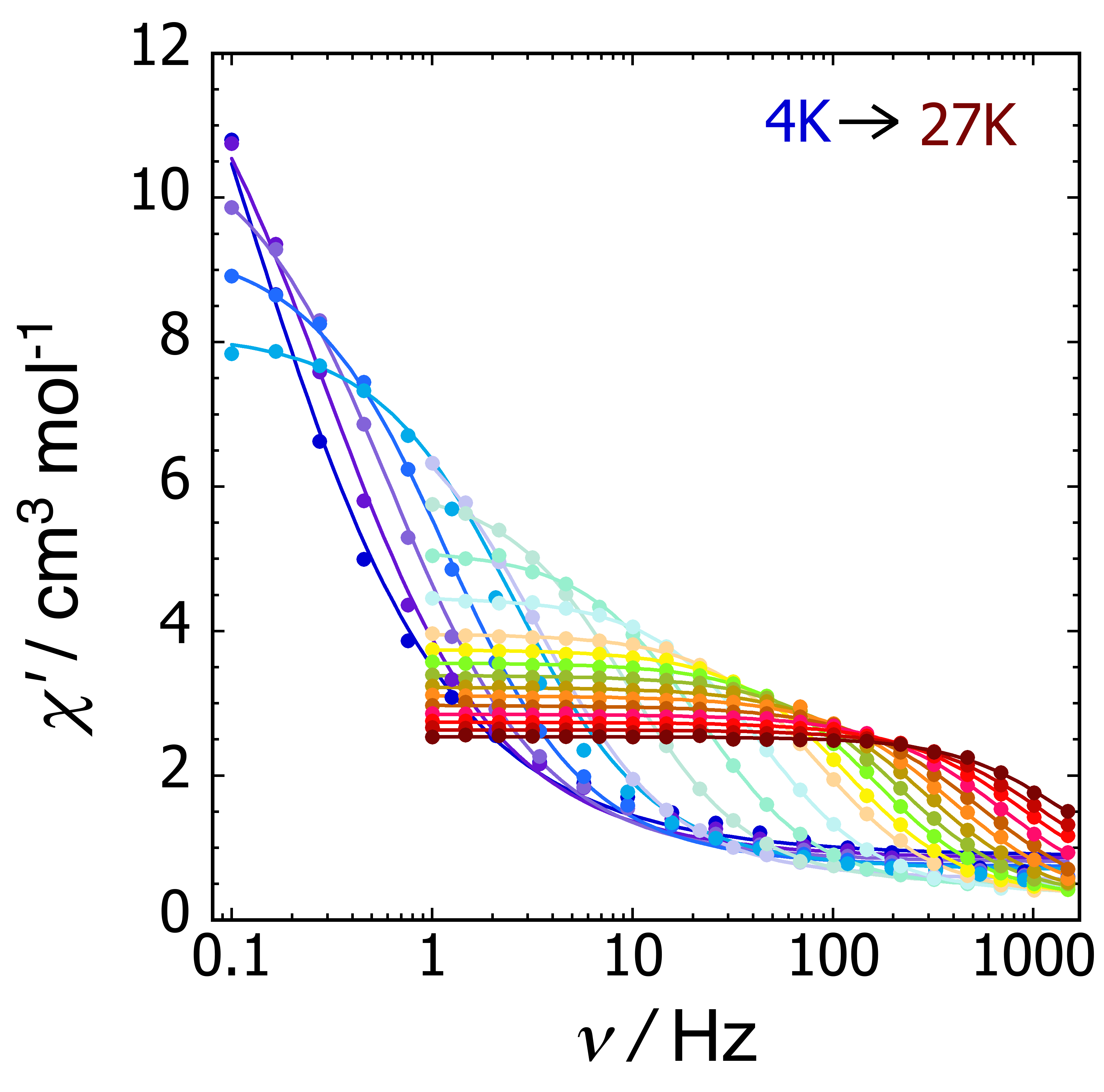


**Figure S25.** Frequency dependence of the in-phase susceptibility (*χ*’) for **1_Dy_**·toluene in zero DC field at *ν* = 1-1000 Hz and temperatures of 4-27 K.


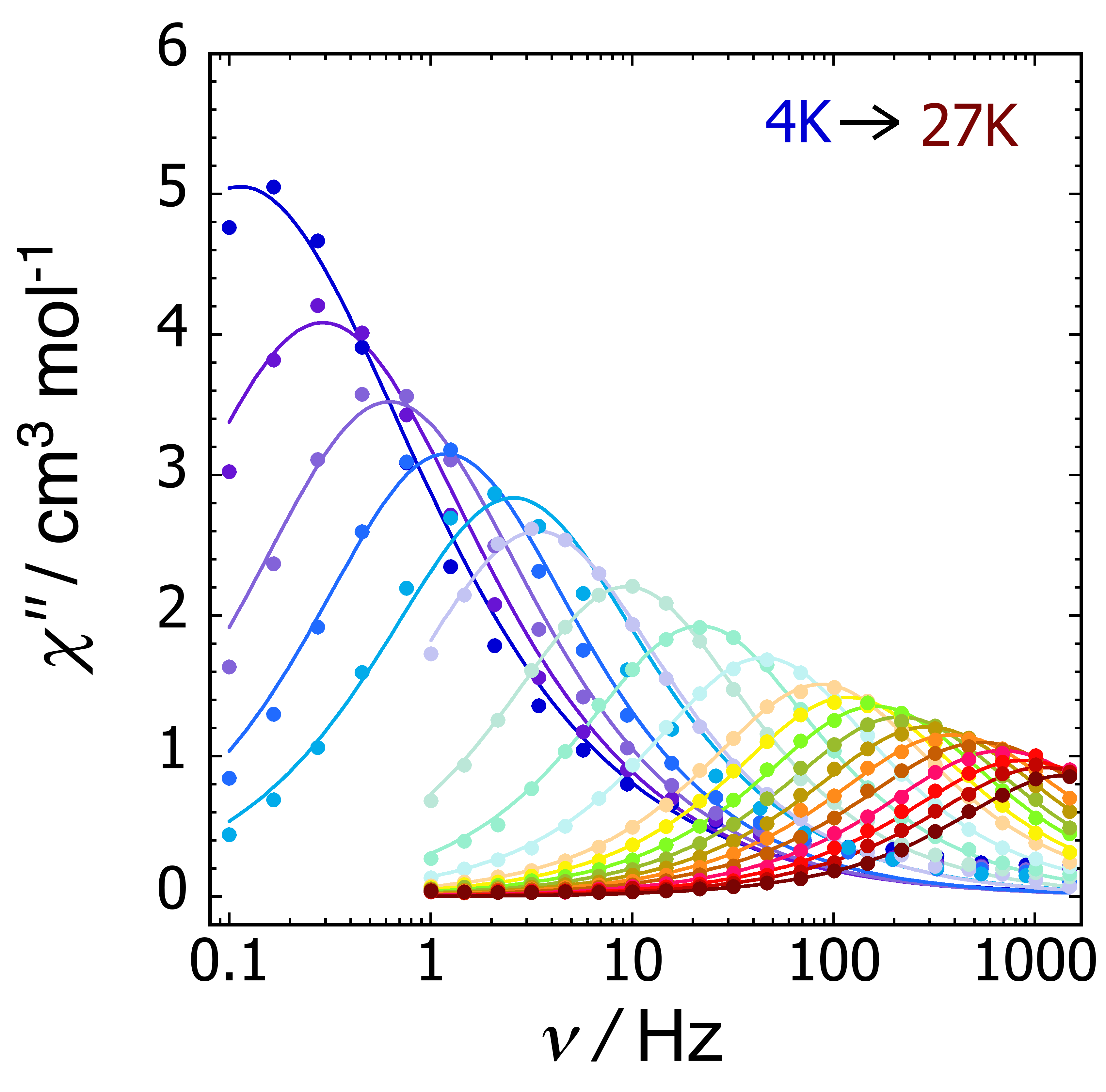


**Figure S26.** Frequency dependence of the out-of-phase susceptibility (*χ*”) for **1_Dy_**·toluene in zero DC field at *ν* = 1-1000 Hz and temperatures of 4-27 K.


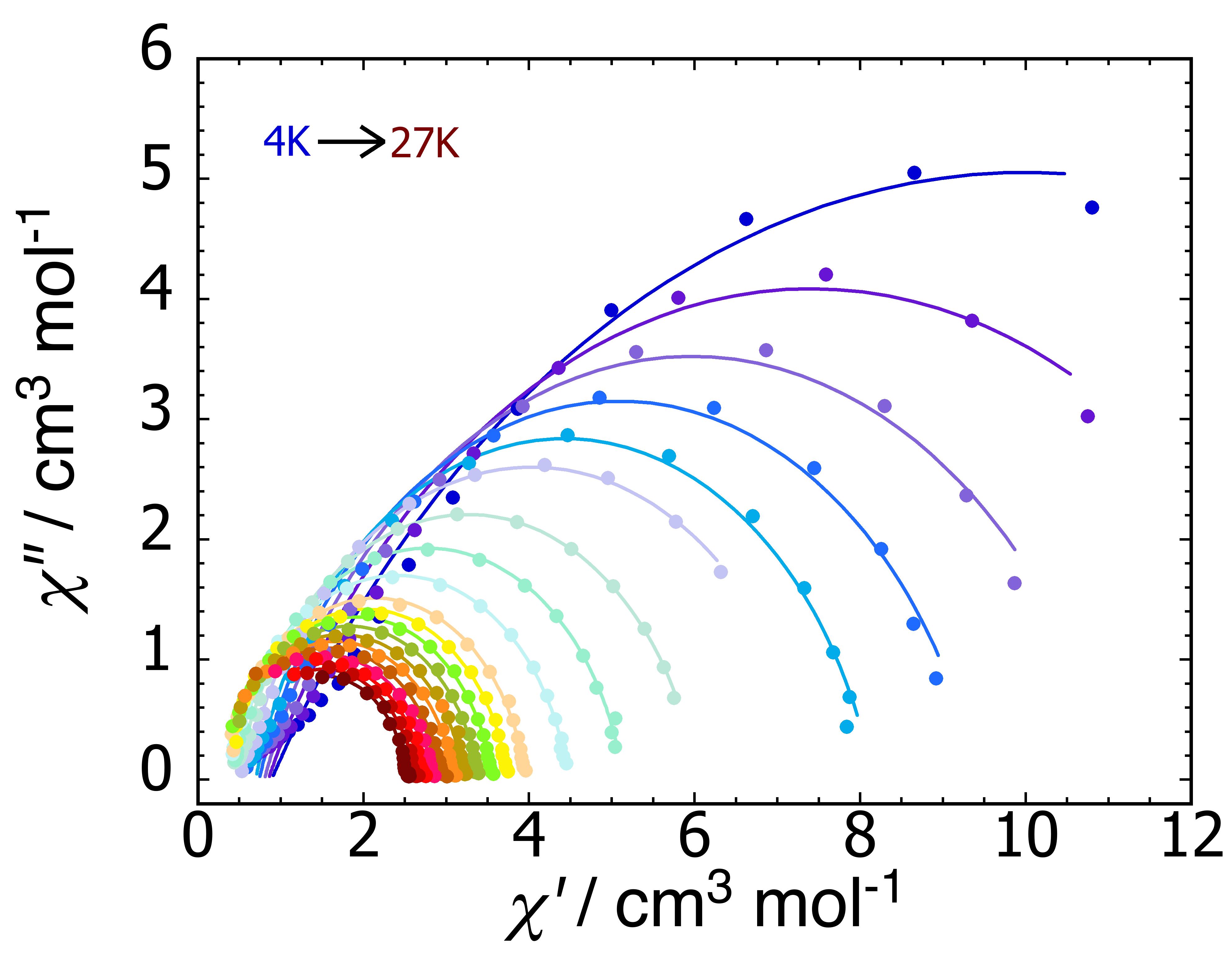


**Figure S27.** Cole-Cole plots for the AC susceptibilities in zero DC field for **1_Dy_**·toluene from 4-27 K. Solid line represents fits to the data using equations S1 and S2.

$\chi^{'}\left( \nu_{ac} \right)$= $\chi_{S}$+ $\frac{(\chi_{T}-\chi_{S})[1+\left( 2\pi\nu_{ac}\tau\right)^{\left( 1-\alpha\right)}Sin(\frac{\alpha\pi}{2})]}{1+{2\left( 2\pi\nu_{ac}\tau\right)}^{\left( 1-\alpha\right)}Sin\left( \frac{\alpha\pi}{2} \right)+\left( 2\pi\nu_{ac}\tau\right)^{2\left( 1-\alpha\right)}}$ (eqn. S1)

$\chi^{''}\left( \nu_{ac} \right)$= $\frac{(\chi_{T}-\chi_{S})\left( 2\pi\nu_{ac}\tau\right)^{\left( 1-\alpha\right)}Cos(\frac{\alpha\pi}{2})}{1+{2\left( 2\pi\nu_{ac}\tau\right)}^{\left( 1-\alpha\right)}Sin\left( \frac{\alpha\pi}{2} \right)+\left( 2\pi\nu_{ac}\tau\right)^{2\left( 1-\alpha\right)}}$ (eqn. S2)

**Table S10.** Relaxation fitting parameters for **1_Dy_**·toluene corresponding to Figure S27.

| ***T*/K** | ***τ*/s** | ***χ*_S_/cm^3^mol^–1^** | ***χ*_T_/cm^3^mol^–1^** | ***α*** |
| --- | --- | --- | --- | --- |
| 4.9997 | 0.54590 | 0.28344 | 4.6518 | 0.29021 |
| 6.0001 | 0.25459 | 0.26751 | 3.7093 | 0.23762 |
| 6.9990 | 0.13380 | 0.24677 | 3.1453 | 0.20169 |
| 7.9994 | 0.073369 | 0.23213 | 2.7190 | 0.17183 |
| 8.9993 | 0.046732 | 0.18694 | 2.5022 | 0.18145 |
| 11.019 | 0.016860 | 0.16068 | 2.0129 | 0.14527 |
| 13.027 | 0.0073401 | 0.13933 | 1.7136 | 0.12861 |
| 14.997 | 0.0035452 | 0.11875 | 1.4928 | 0.12172 |
| 16.999 | 0.0018404 | 0.10616 | 1.3227 | 0.11959 |
| 17.999 | 0.0013555 | 0.10289 | 1.2505 | 0.12224 |
| 19.000 | 0.00098422 | 0.089684 | 1.1888 | 0.12435 |
| 19.998 | 0.00074392 | 0.092362 | 1.1299 | 0.12457 |
| 21.000 | 0.00054267 | 0.078280 | 1.0756 | 0.13479 |
| 21.998 | 0.00041288 | 0.074944 | 1.0360 | 0.14234 |
| 22.999 | 0.00029436 | 0.051569 | 0.99161 | 0.15814 |
| 24.000 | 0.00023235 | 0.084400 | 0.95049 | 0.14145 |
| 24.998 | 0.00018129 | 0.11238 | 0.91393 | 0.13620 |
| 25.999 | 0.00014362 | 0.13276 | 0.87913 | 0.12433 |
| 27.000 | 0.00011214 | 0.17013 | 0.84669 | 0.10402 |


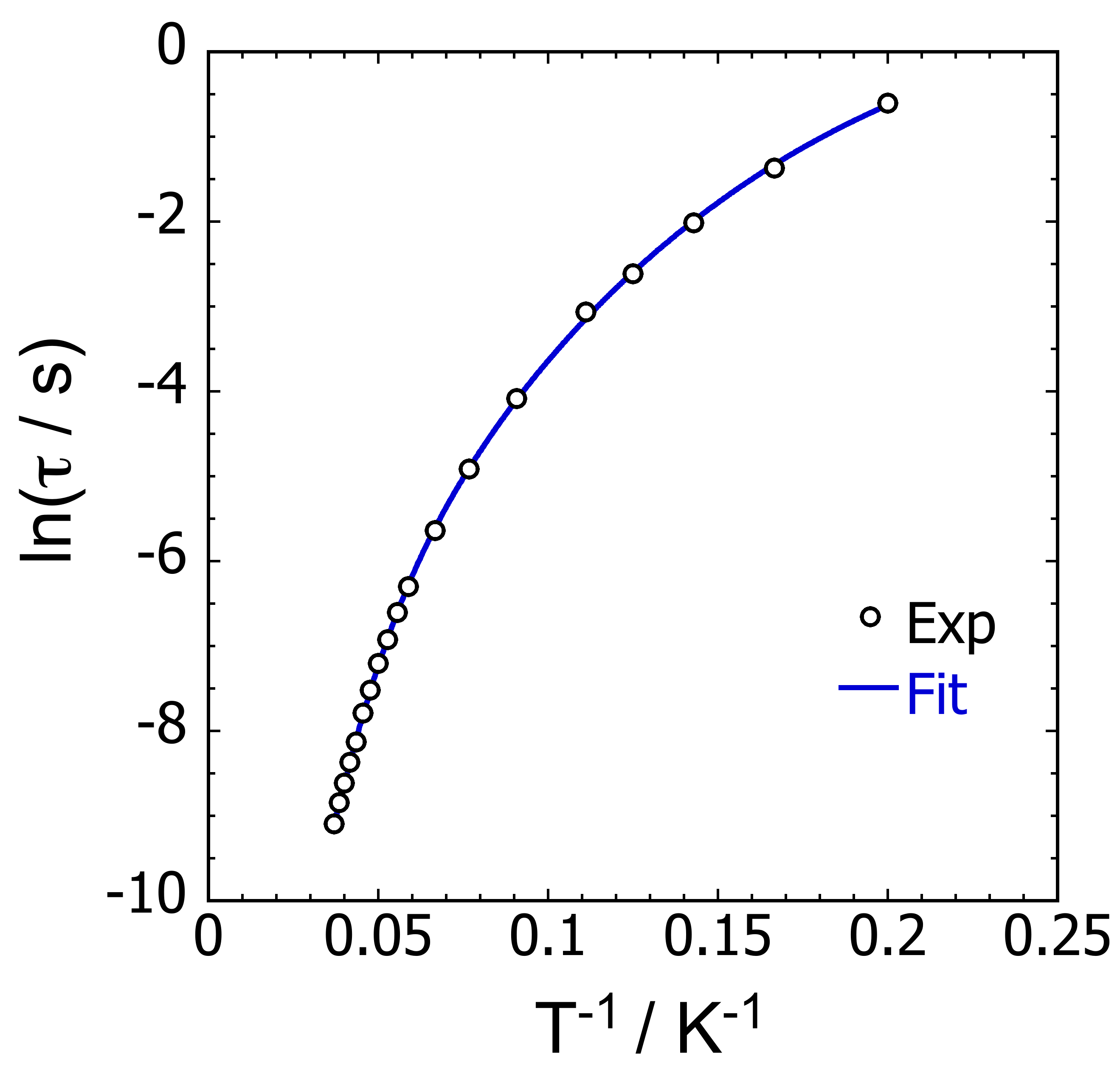


**Figure S28.** Plot of $\ln(\tau/s)$ vs. $T^{-1}$ for **1_Dy_**·toluene. Circles are experimental data points and the blue line is the best fit considering Orbach, Raman, and QTM processes using $\tau^{-1}= \tau_{0}^{-1}e^{-U_{\mathrm{eff}}/k_{B}T}+CT^{n}+ {\tau_{QTM}}^{-1}$, giving: *U*_eff_ = 138 ± 6.9 cm^-1^, *τ*_0_ = 1.44 × 10^-7^ s, *C* = 6.23 x 10^-4^ ± 9.95 x 10^-5^ s^-1^ K^-n^, *n* = 4.77± 0.06, and $\tau_{QTM}$= 1.971 ± 0.335 s.


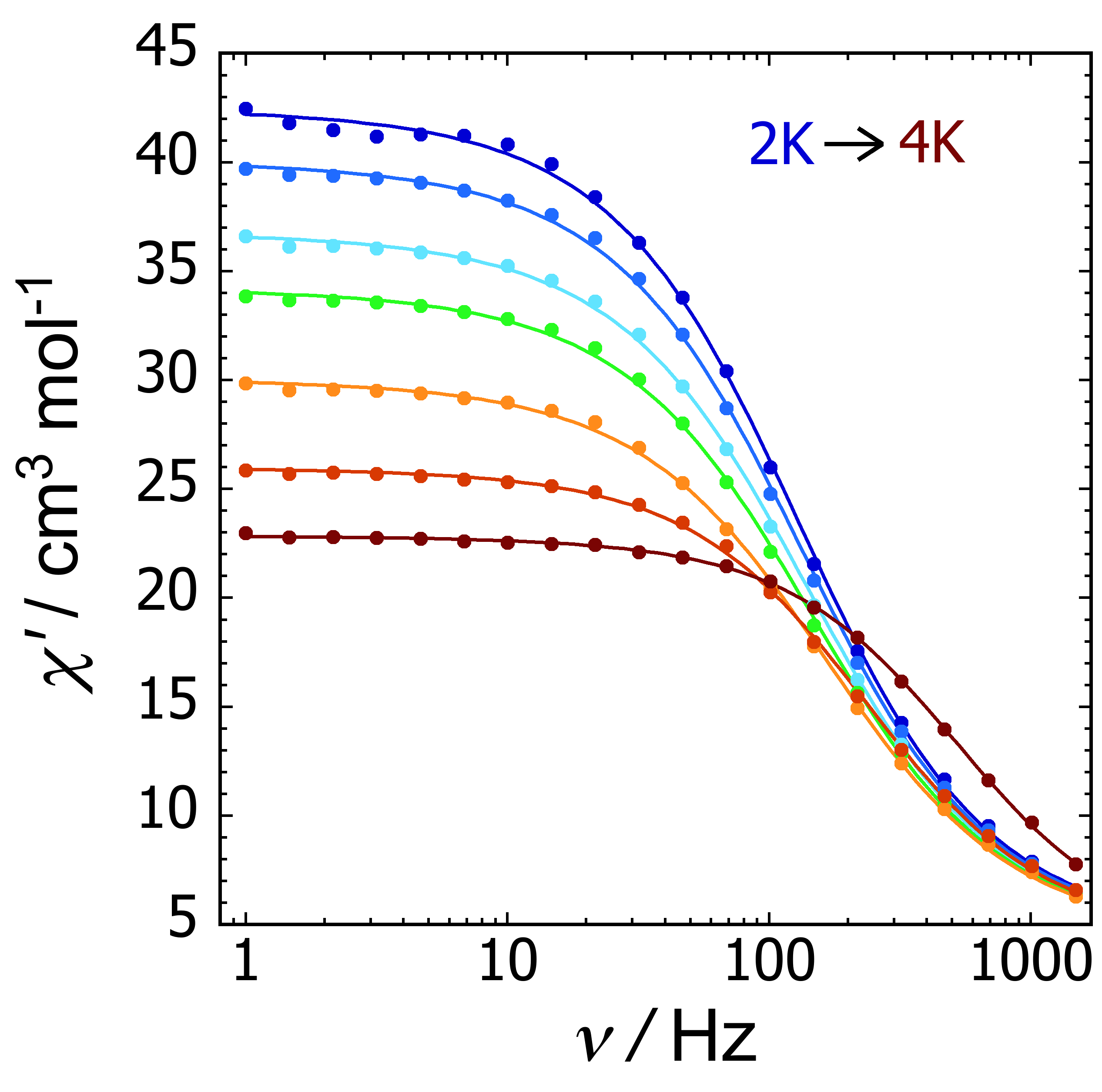


**Figure S29.** Frequency dependence of the real component of the AC susceptibility *χ*' for [K(2.2.2-crypt)][**2_Dy_**]·1.75(hexane) in zero DC field at *ν* = 1-1000 Hz and temperatures of 2-4 K.


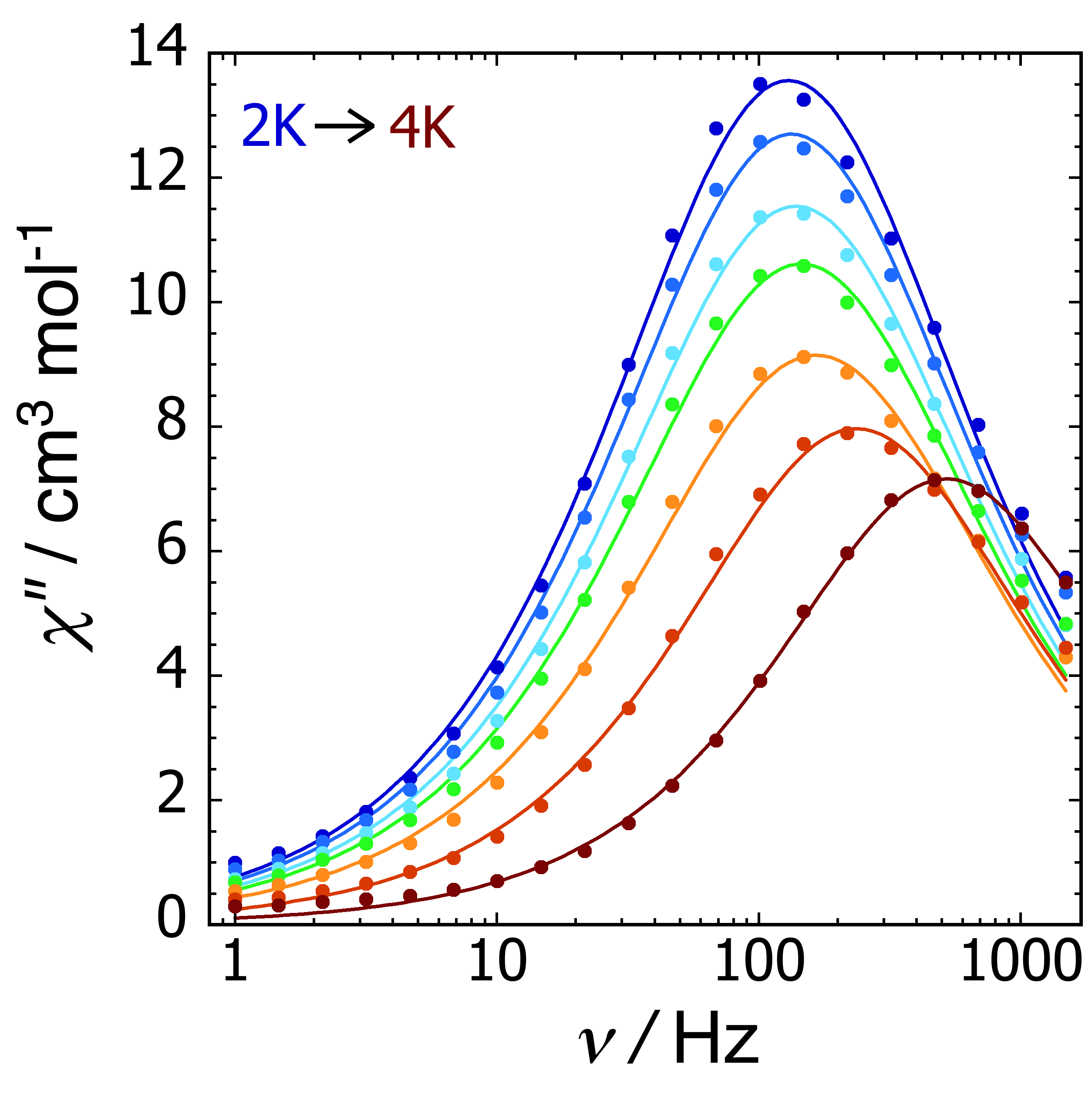


**Figure S30.** Frequency dependence of the out-of-phase susceptibility (*χ*”) for [K(2.2.2-crypt)][**2_Dy_**]·1.75(hexane) in zero DC field at *ν* = 1-1000 Hz and temperatures of 2-4 K.


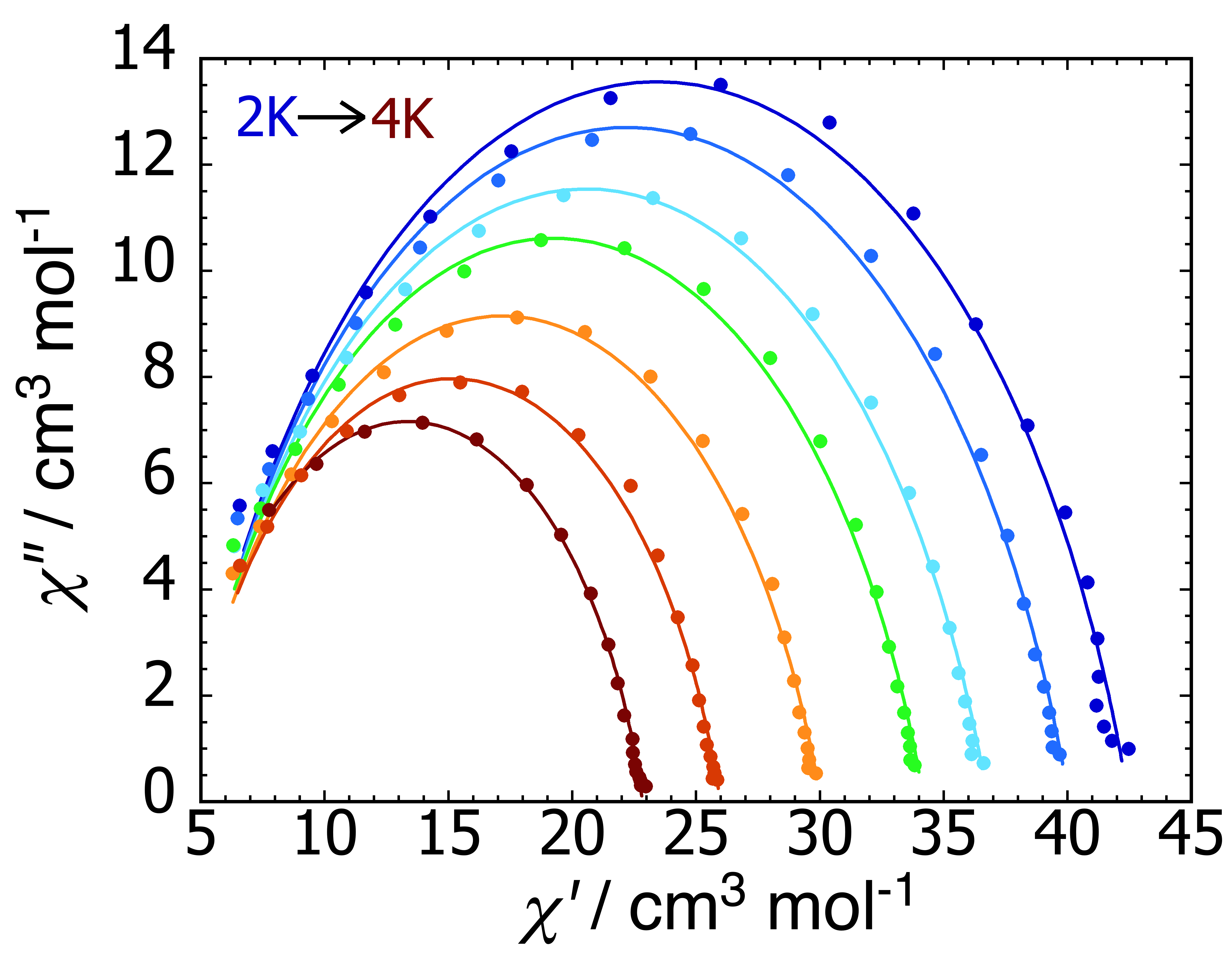


**Figure S31.** Cole-Cole plots for the AC susceptibilities in zero DC field for [K(2.2.2-crypt)][**2_Dy_**]·1.75(hexane) from 2-4 K. Solid line represents fits to the data using equations S1 and S2.

**Table S11.** Relaxation fitting parameters for [K(2.2.2-crypt)][**2_Dy_**]·1.75(hex) corresponding to Figure S31.

| ***T*/K** | ***τ*/s** | ***χ*_S_/cm^3^mol^–1^** | ***χ*_T_/cm^3^mol^–1^** | ***α*** |
| --- | --- | --- | --- | --- |
| 2.027305 | 0.0012226 | 1.4543 | 14.161 | 0.21252 |
| 2.197824 | 0.0011937 | 1.4529 | 13.355 | 0.21262 |
| 2.400056 | 0.0011477 | 1.4459 | 12.261 | 0.21258 |
| 2.599982 | 0.0010955 | 1.4278 | 11.403 | 0.21425 |
| 2.999878 | 0.00096394 | 1.4269 | 10.013 | 0.21352 |
| 3.499596 | 0.00067528 | 1.4475 | 8.6569 | 0.19138 |
| 3.998460 | 0.00030712 | 1.2934 | 7.6129 | 0.17616 |


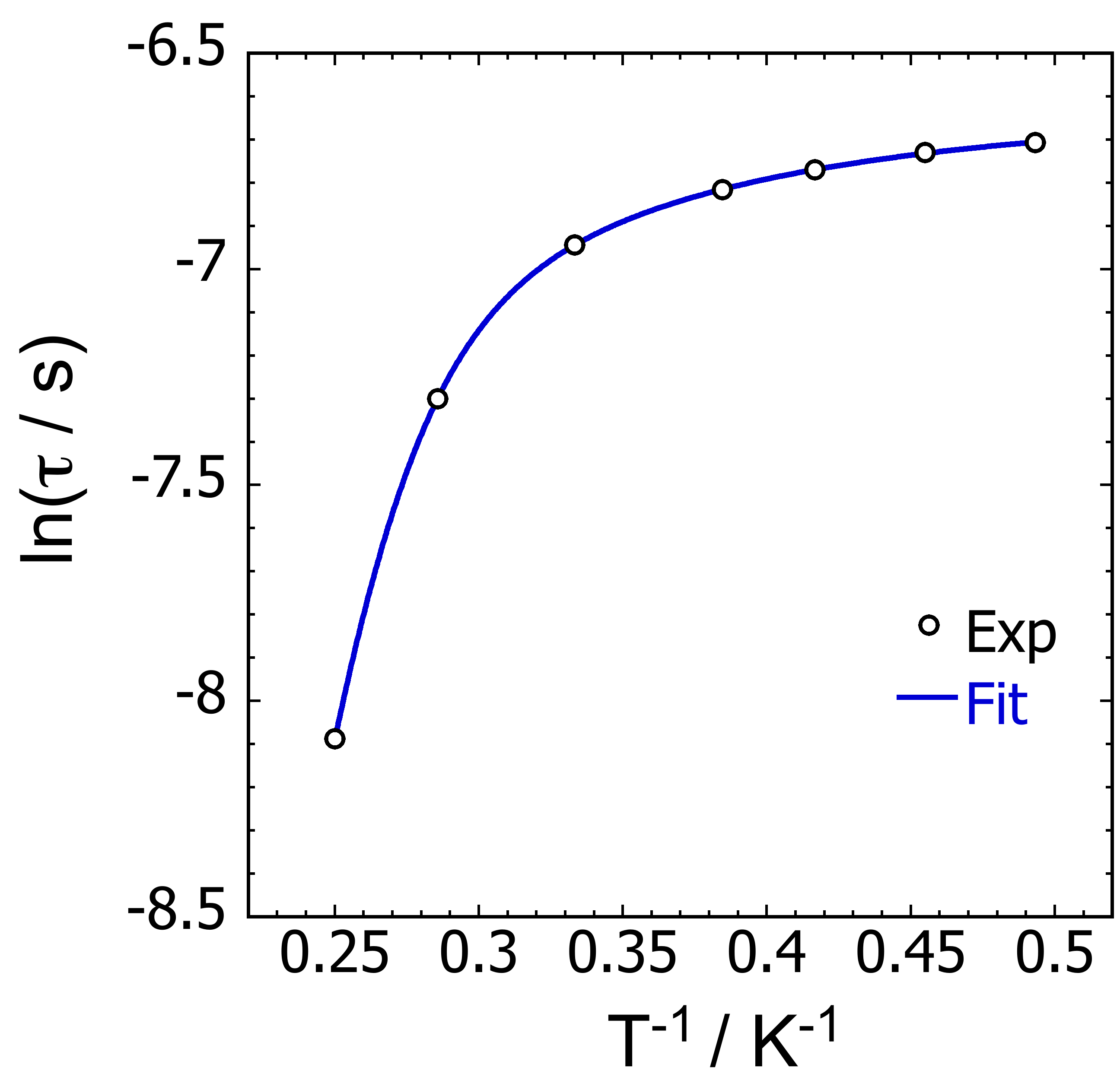


**Figure S32.** Plot of $\ln(\tau/s)$ vs. $T^{-1}$ for [K(2.2.2-crypt)][**2_Dy_**]·1.75(hexane). Circles are experimental data points and the blue line is the best fit considering Orbach, Raman, and QTM processes using $\tau^{-1}= \tau_{0}^{-1}e^{-U_{\mathrm{eff}}/k_{B}T}+CT^{n}+ {\tau_{QTM}}^{-1}$, giving: *U*_eff_ = 36 ± 1.4 cm^-1^, *τ*_0_ = 1.14 × 10^-9^ s, *C* = 5.38 ± 1.60 s^-1^ K^-n^, *n* = 3.54 ± 0.25, and $\tau_{QTM}$= 1.33 x 10^-3^ ± 1.41 x 10^-5^ s.


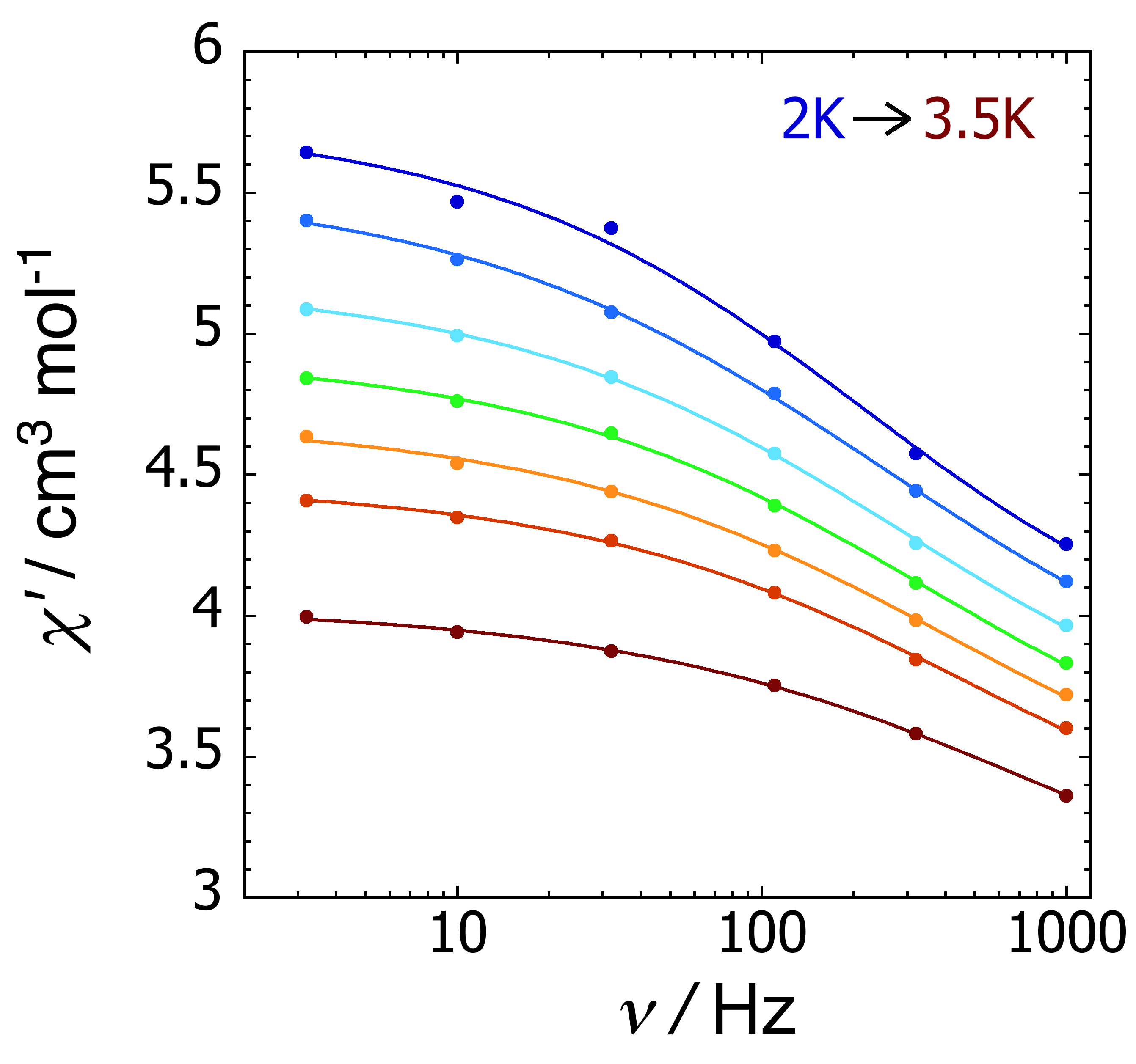


**Figure S33.** Frequency dependence of the real component of the AC susceptibility (*χ*') for [K(2.2.2-crypt)]_2_[**3_Dy_**]·2(hexane) in zero DC field at *ν* = 1-1000 Hz and temperatures of 2-3.5 K.


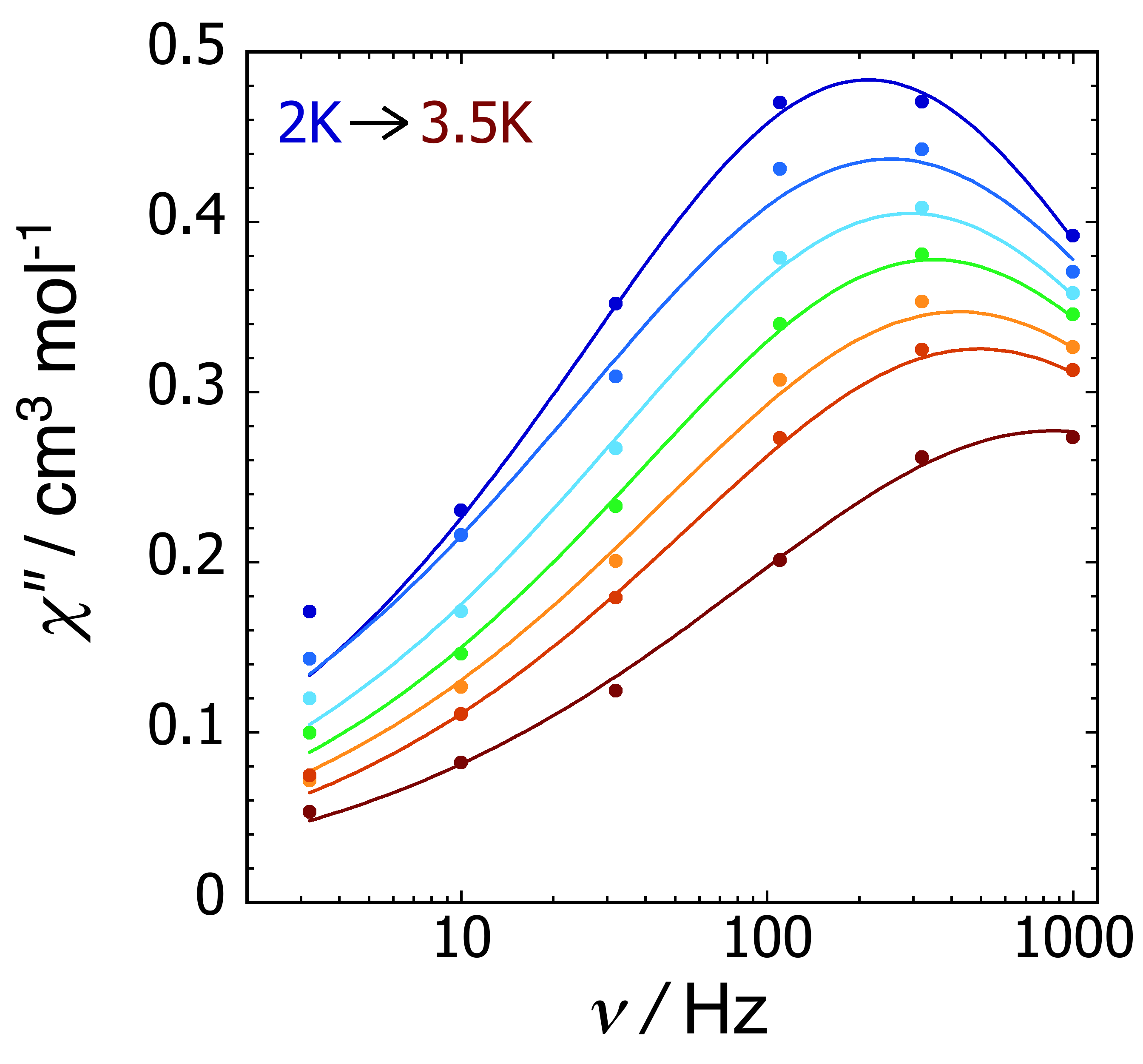


**Figure S34.** Frequency dependence of the out-of-phase susceptibility (*χ*”) for [K(2.2.2-crypt)]_2_[**3_Dy_**]·2(hexane) in zero DC field at *ν* = 1-1000 Hz and temperatures of 2-4 K.


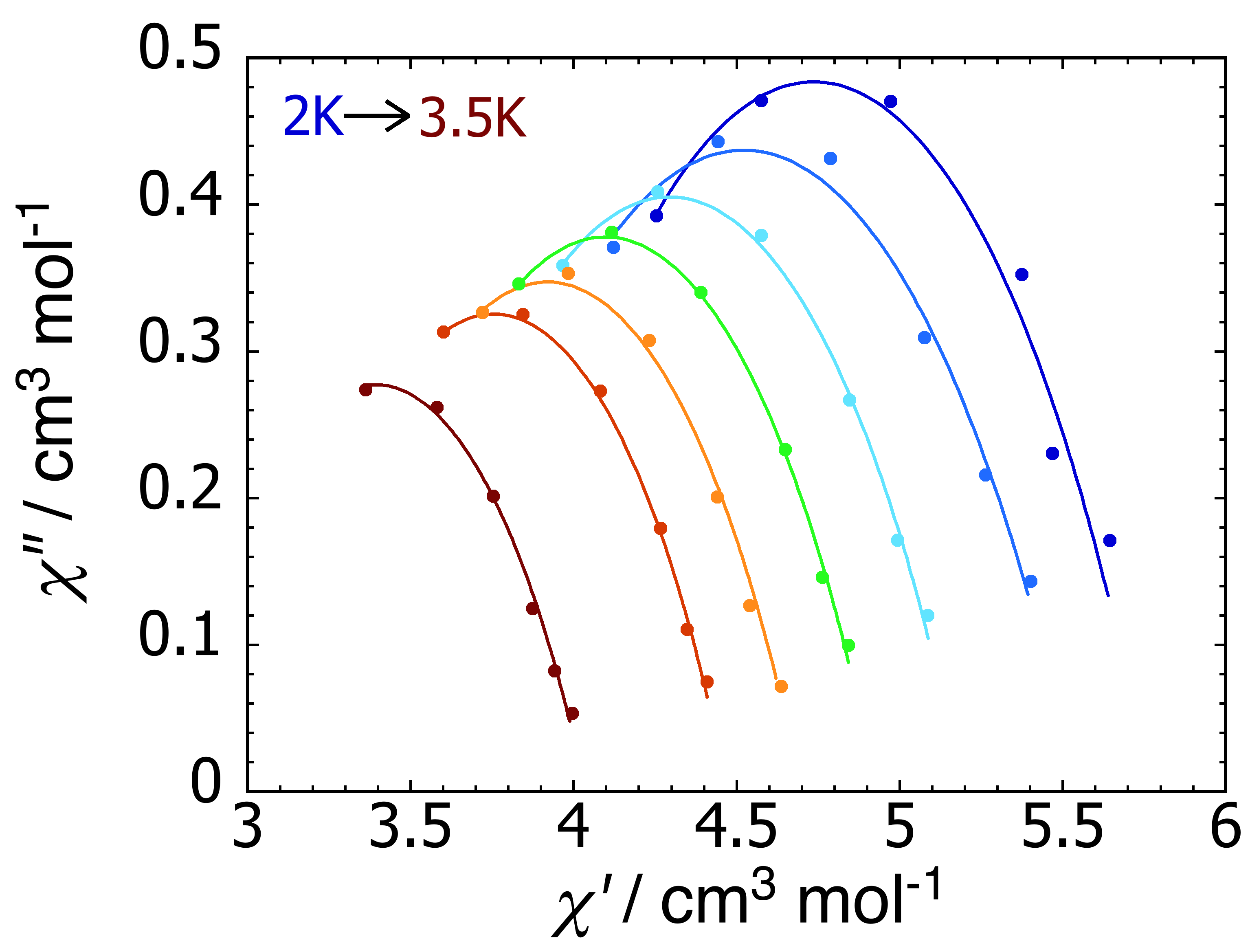


**Figure S35.** Cole-Cole plots for the AC susceptibilities in zero DC field for [K(2.2.2-crypt)]_2_[**3_Dy_**]·2(hexane) from 2-3.5 K. Solid line represents fits to the data using equations S1 and S2.

**Table S12.** Relaxation fitting parameters for [K(2.2.2-crypt)]_2_[**3_Dy_**]·2(hexane) corresponding to Figure S35.

| ***T*/K** | ***τ*/s** | ***χ*_S_/cm^3^mol^–1^** | ***χ*_T_/cm^3^mol^–1^** | ***α*** |
| --- | --- | --- | --- | --- |
| 2.0121 | 0.00074363 | 3.7078 | 5.7656 | 0.44054 |
| 2.2002 | 0.00063324 | 3.5030 | 5.5405 | 0.48397 |
| 2.4003 | 0.00054084 | 3.3961 | 5.1924 | 0.46043 |
| 2.6003 | 0.00045323 | 3.2653 | 4.9294 | 0.45720 |
| 2.7999 | 0.00037789 | 3.1433 | 4.6976 | 0.46497 |
| 2.9999 | 0.00032337 | 3.0382 | 4.4717 | 0.45734 |
| 3.4998 | 0.00018501 | 2.7484 | 4.0369 | 0.48258 |


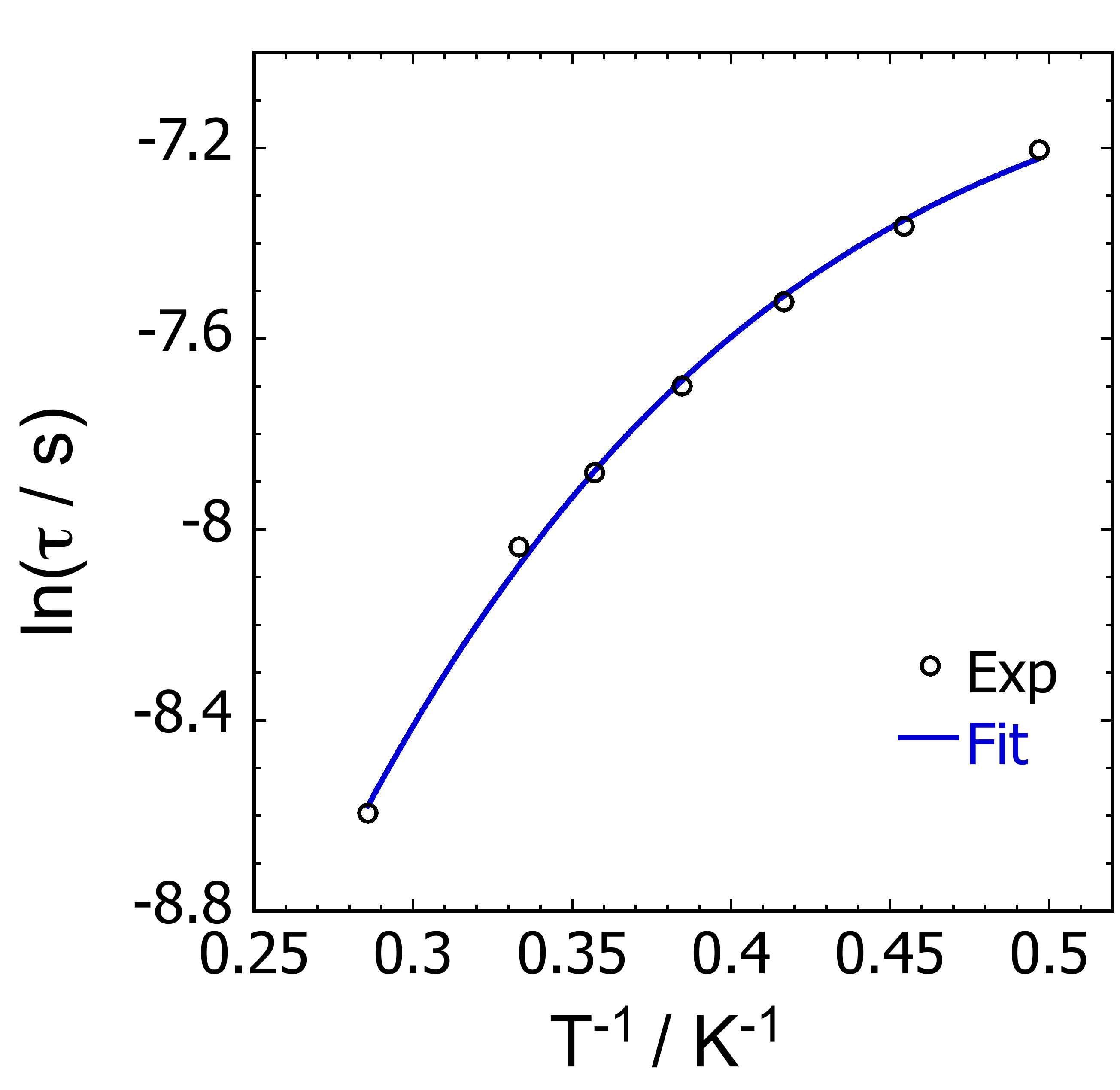


**Figure S36.** Plot of $\ln(\tau/s)$ vs. $T^{-1}$ for [K(crypt)]_2_[**3_Dy_**]·2(hexane). Circles are experimental data points and the blue line is the best fit considering Raman and QTM processes using $\tau^{-1}= CT^{n}+ {\tau_{QTM}}^{-1}$, giving: *C* = 19.93 ± 6.974 s^-1^ K^-n^, *n* = 4.30 ± 0.27, and $\tau_{QTM}$= 1.035 x 10^-3^ ± 9.52 x 10^-4^ s.


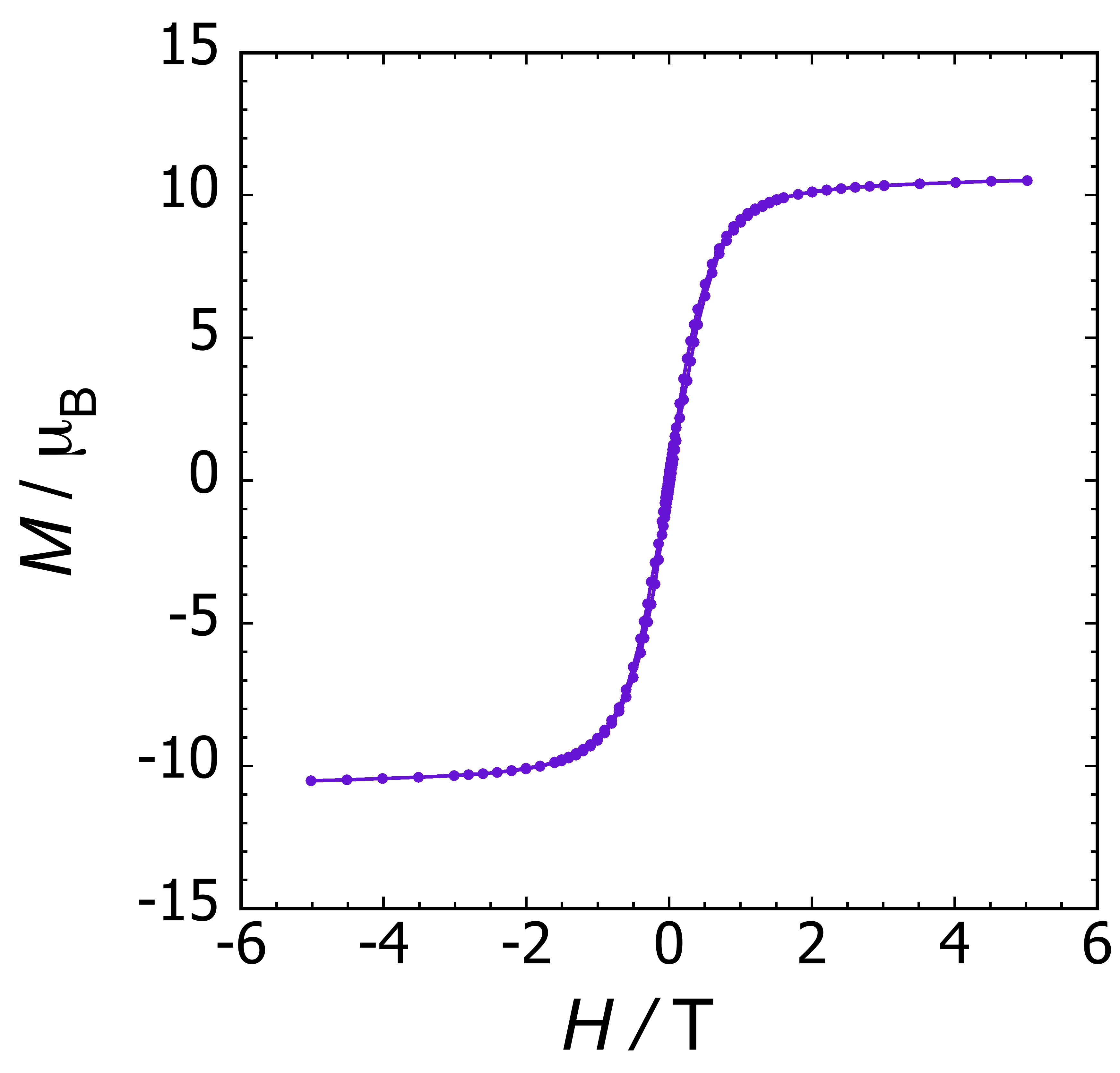

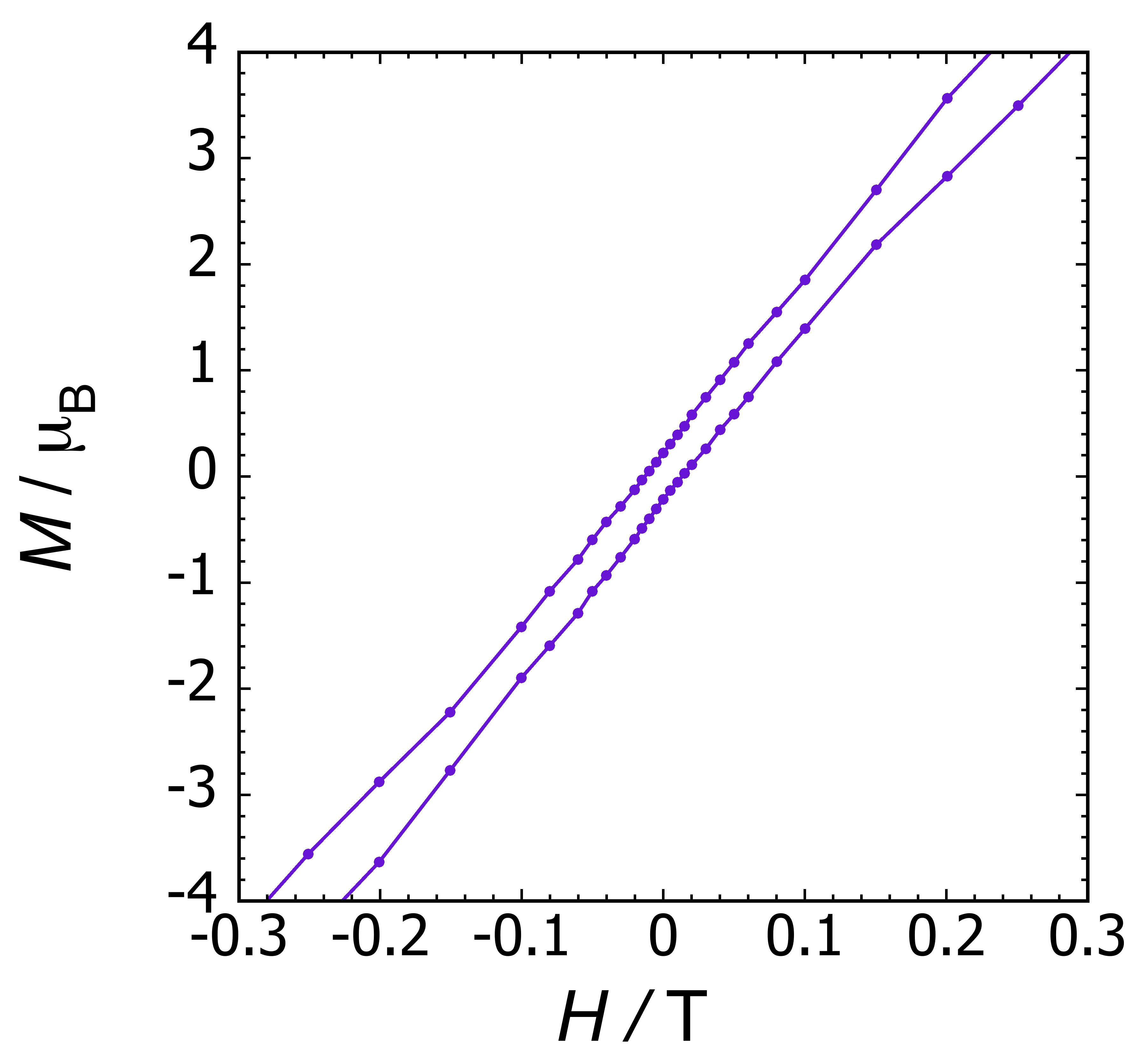


**Figure S37.** Magnetic hysteresis plot for **1_Dy_**·toluene at 2K. Sweep rates: 0.23 mT s^–1^ │0.00-0.02│T; 0.47 mT s^–1^ │0.02-0.06│ T; 0.95 mT s^–1^ │0.06-0.1│ T; 2.17 mT s^–1^ │0.1-0.4│ T; 4 mT s^–1^ │0.4-1.6│ T; 6.66 mT s^–1^ │1.6-3│ T; 11.6 mT s^–1^ │3-5.0│ T.

**
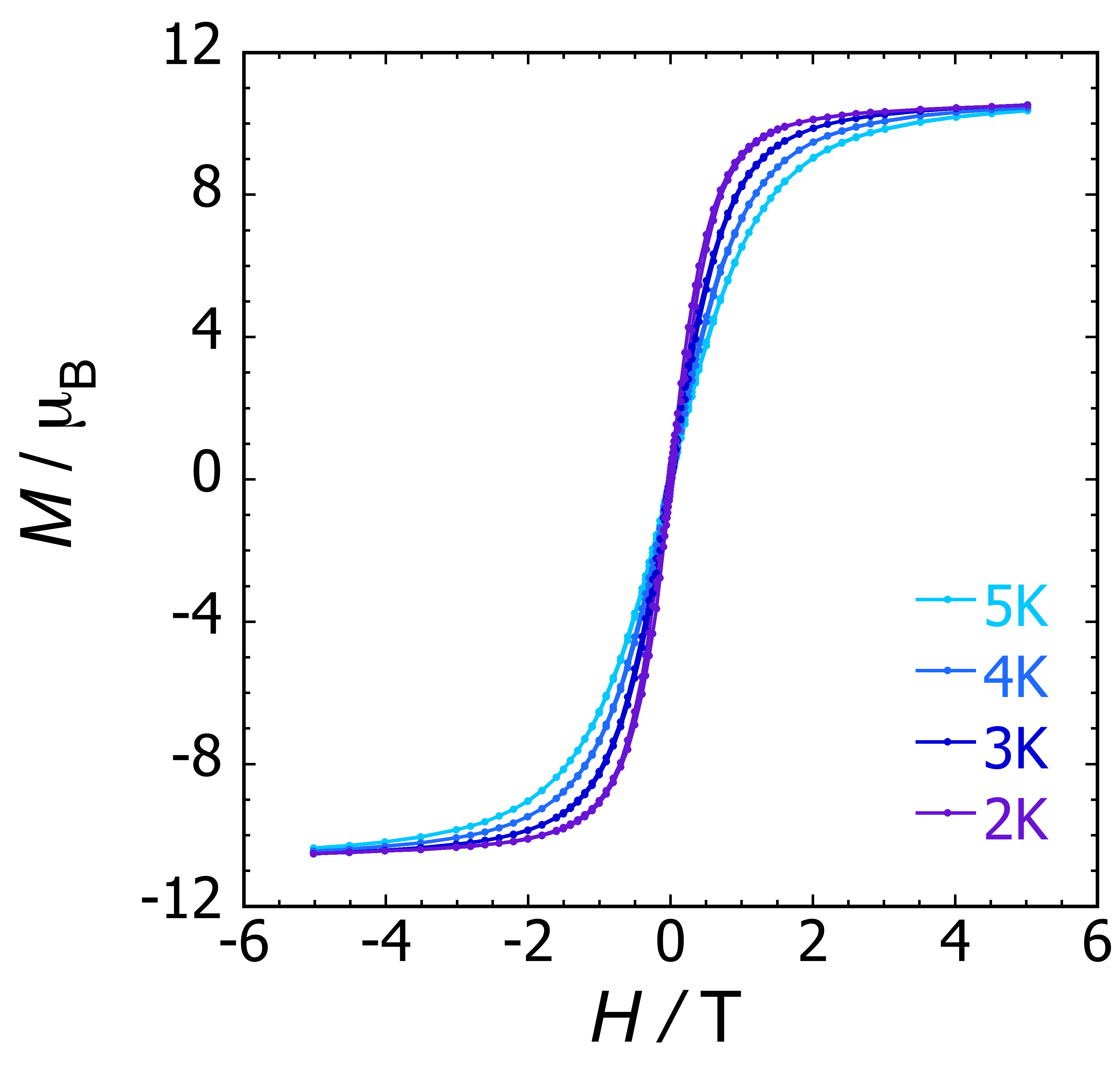
**

**Figure S38.** Magnetic hysteresis plot for **1_Dy_**·toluene at 2K, 3K, 4K, and 5K. Sweep rates: 0.23 mT s^–1^ │0.00-0.02│T; 0.47 mT s^–1^ │0.02-0.06│ T; 0.95 mT s^–1^ │0.06-0.1│ T; 2.17 mT s^–1^ │0.1-0.4│ T; 4 mT s^–1^ │0.4-1.6│ T; 6.6 mT s^–1^ │1.6-3│ T; 11.6 mT s^–1^ │3-5.0│ T.


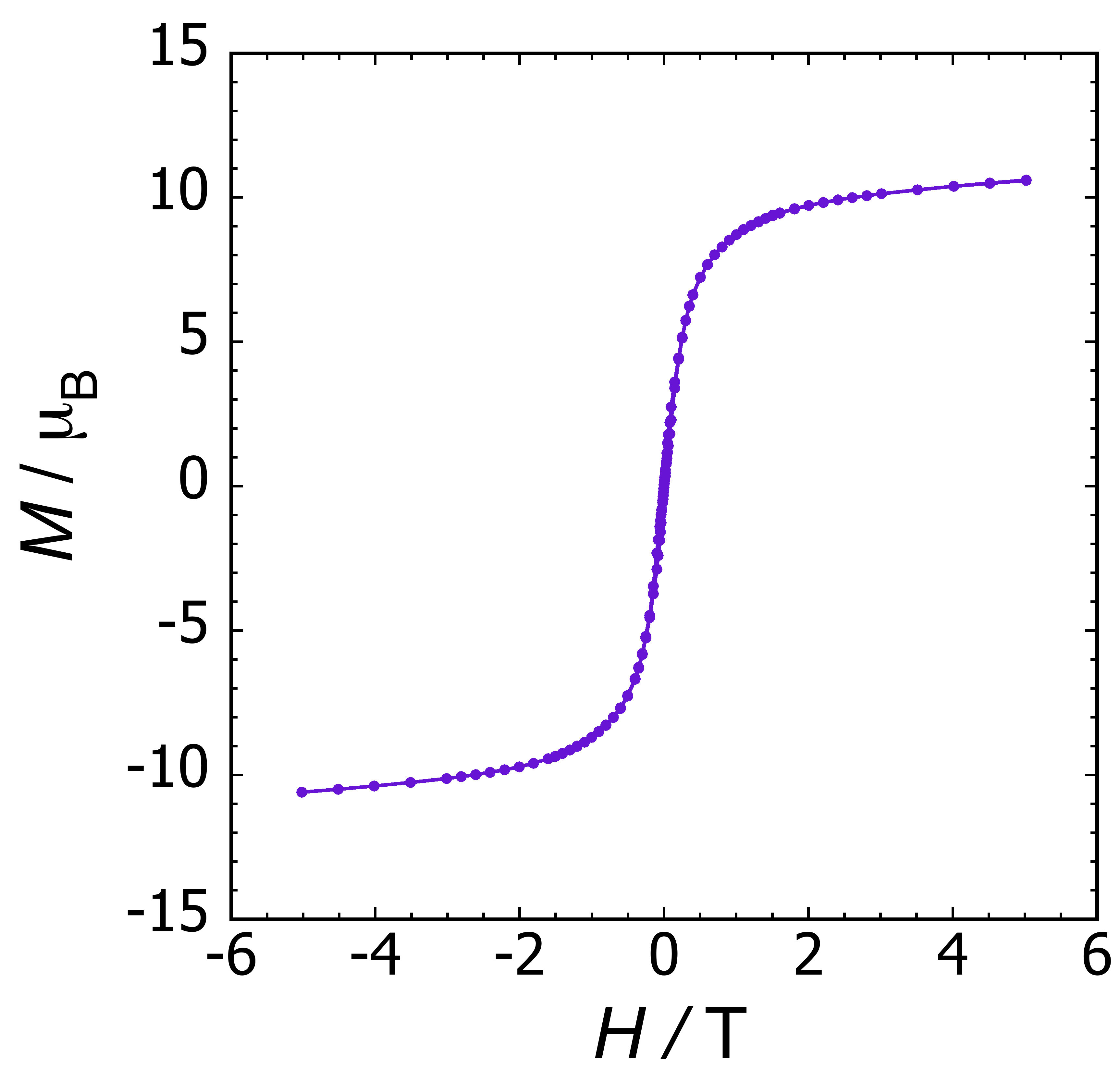

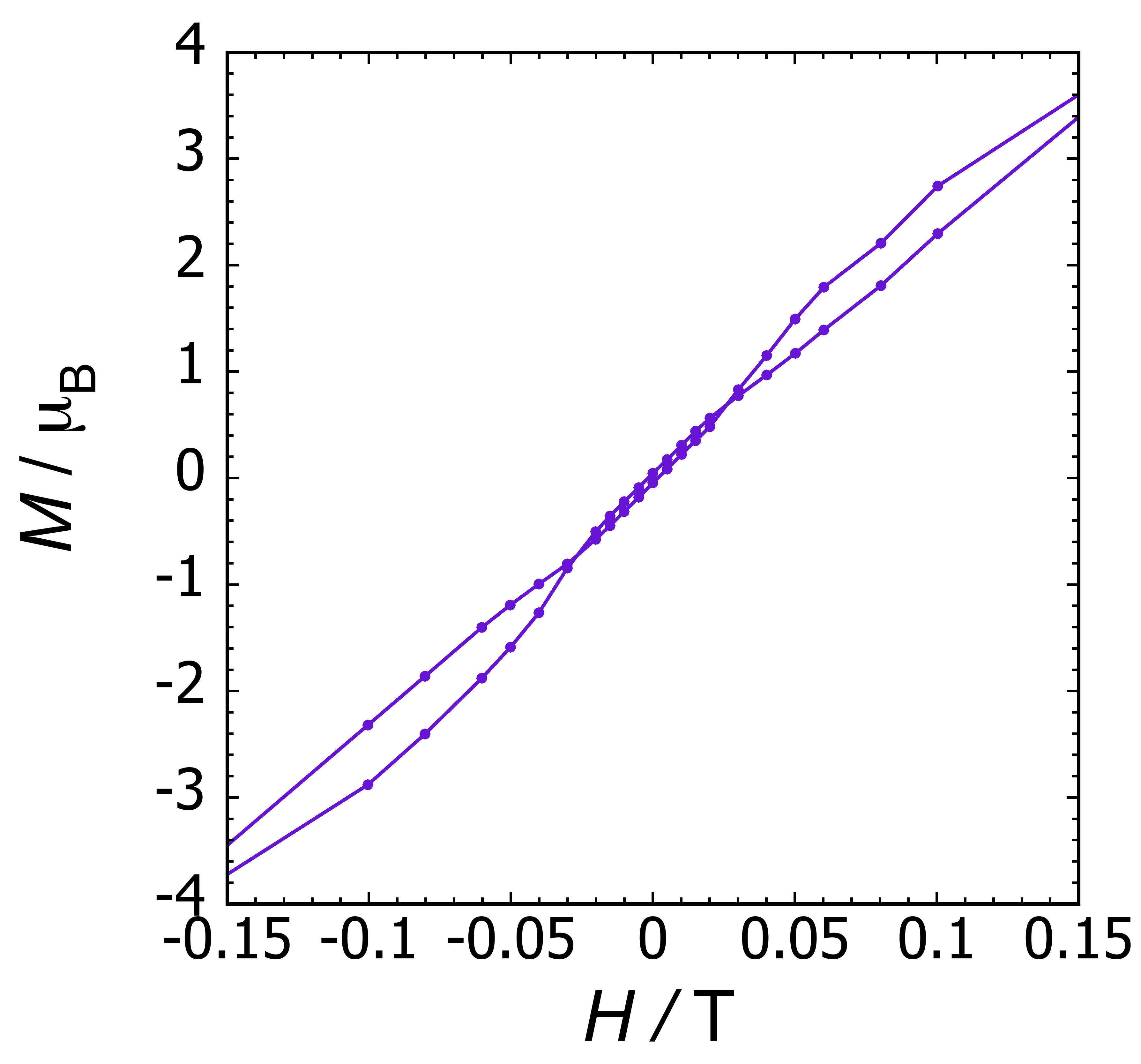


**Figure S39.** Magnetic hysteresis plot for [K(2.2.2-crypt)][**2_Dy_**]·1.75(hexane) at 2K. Sweep rates: 0.25 mT s^–1^ │0.00-0.02│T; 0.45 mT s^–1^ │0.02-0.06│ T; 0.86 mT s^–1^ │0.06-0.1│ T; 2.08 mT s^–1^ │0.1-0.4│ T; 4 mT s^–1^ │0.4-1.6│ T; 6.66 mT s^–1^ │1.6-3│ T; 11.4 mT s^–1^ │3-5.0│ T.


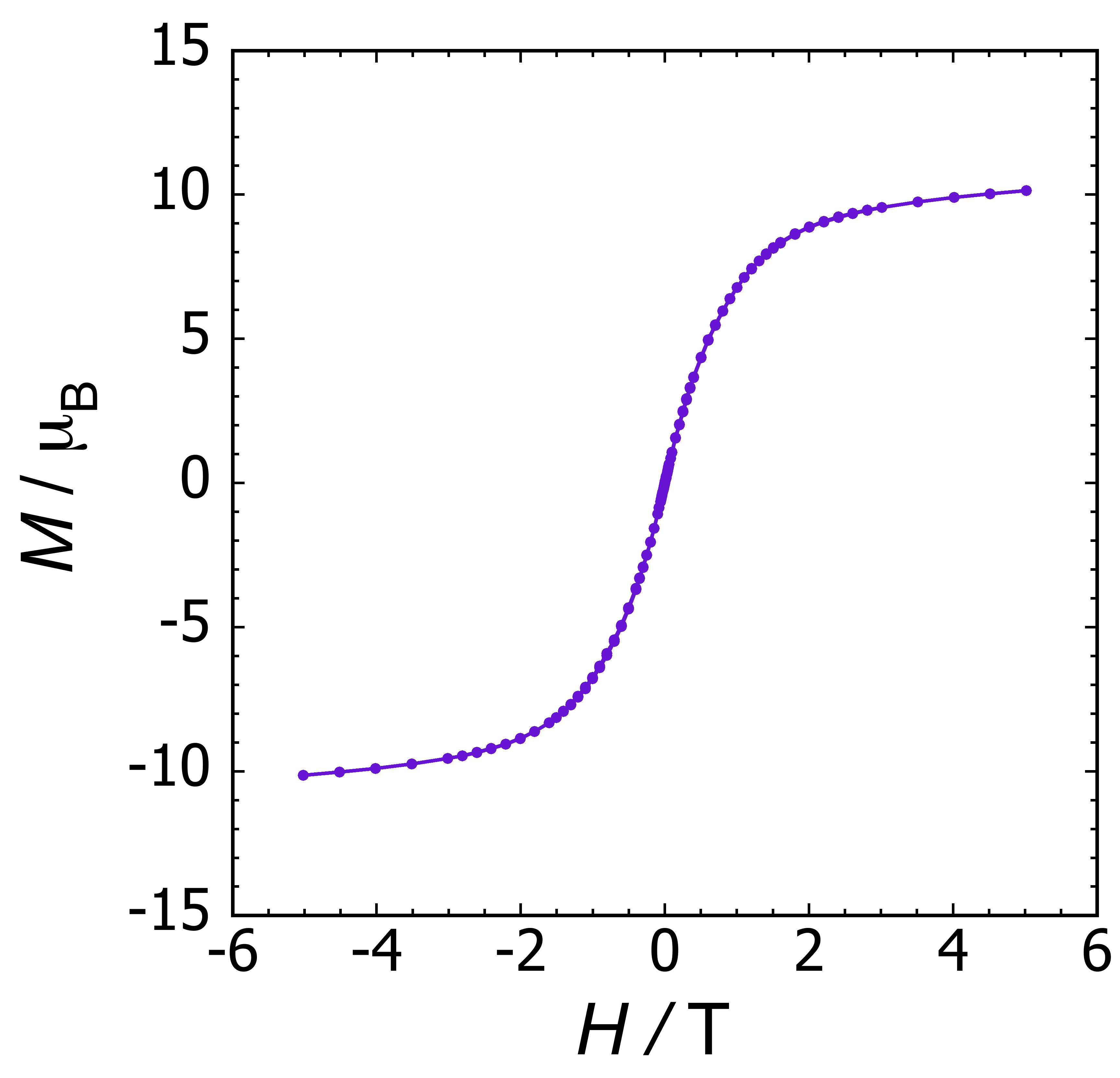

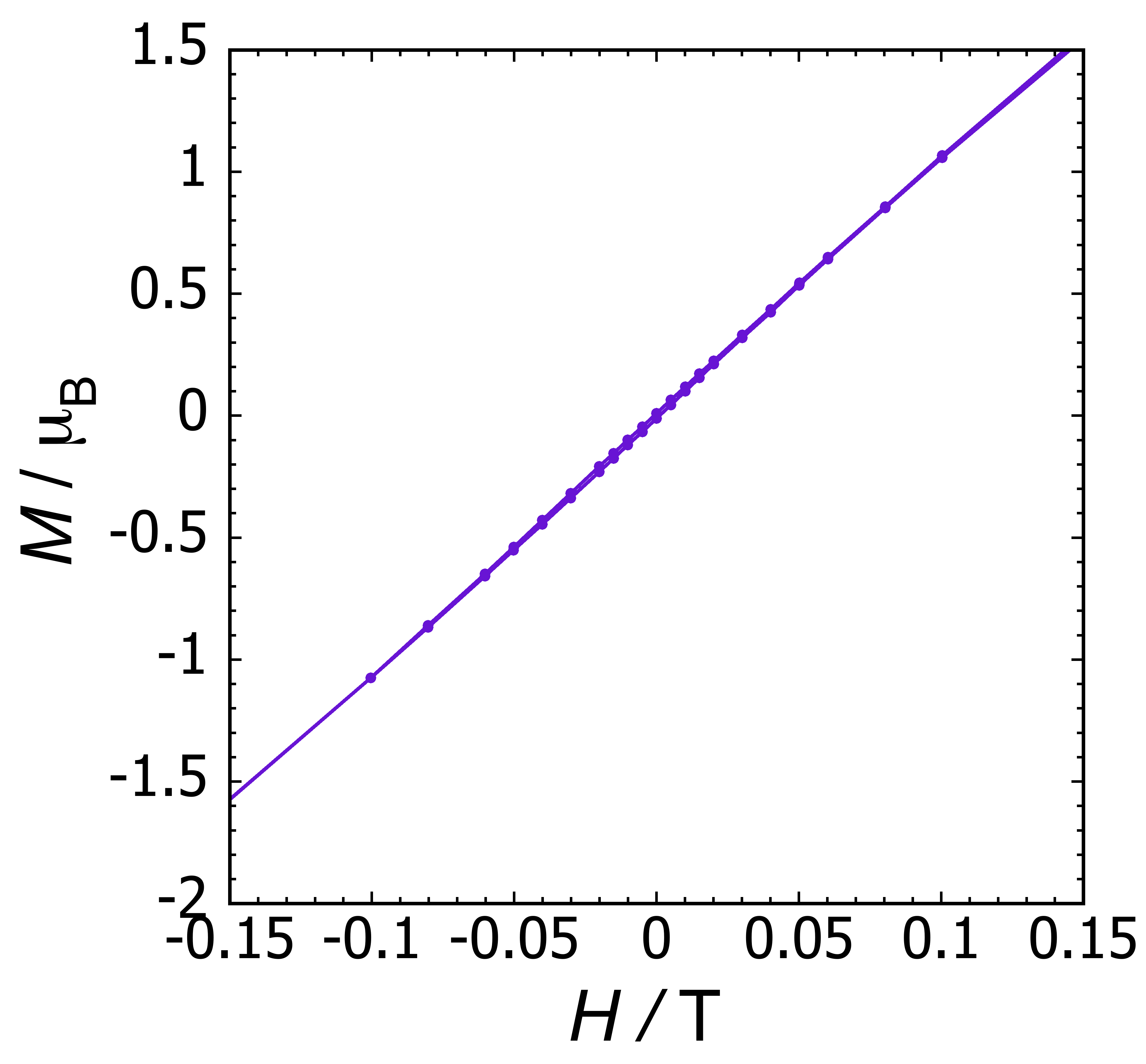


**Figure S40.** Magnetic hysteresis plot for [K(2.2.2-crypt)]_2_[**3_Dy_**]·2(hexane) at 2K. Sweep rates: 0.20 mT s^–1^ │0.00-0.02│T; 0.47 mT s^–1^ │0.02-0.06│ T; 0.95 mT s^–1^ │0.06-0.1│ T; 1.66 mT s^–1^ │0.1-0.4│ T; 4 mT s^–1^ │0.4-1.6│ T; 6.66 mT s^–1^ │1.6-3│ T; 11.4 mT s^–1^ │3-5.0│ T.

**Multireference Calculations**

Multireference calculations were carried out on the coordinates obtained from the X-ray structures using the ORCA 6.0.0 software package.^[104]^ Hydrogen atom positions were optimized at the DFT level using the pure GGA PBE exchange correlation functional,^[105,106]^ keeping constant the position of other atoms. The def2-TZVP basis sets with effective core potential (ECP) were used to treat the core electrons of yttrium throughout the DFT calculations.^[117,118]^ The calculations were of the CASSCF/QDPT/SINGLE_ANISO type, and the DKH (Douglas-Kroll-Hess) Hamiltonian was used throughout the calculations to consider relativistic effects. We employed the SARC2-DKH-QZVP basis set for dysprosium, the SARC-DKH-TZVP basis set for yttrium and tin, DKH-def2-TZVP basis set for silicon, and all other atoms were treated with the DKH-def2-SVP basis set in combination with ‘AutoAux’ auxiliary basis set.^[108,109]^ In the active space, we considered 9 electrons in 7 f-orbitals CAS (9,7) for all complexes. Further, 21 sextets, 128 quartets, and 130 doublets states were solved in the state-averaged (SA) calculations. To consider the spin-orbit coupling, we used the quasi-degenerate perturbation theory (QDPT) approach using SA-CASSCF wave functions.^[119]^ The SINGLE_ANISO and POLY_ANISO module as implemented in ORCA has been used to compute the g-tensor and crystal field parameters of the low-lying excited state using previously calculated spin-orbit states.^[120]^

To quantify the exchange interaction between the Dy^3+^ centers in **1_Dy_** and **3_Dy_**, we simulated the molar magnetic susceptibility using POLY_ANISO with the following Ising-type Hamiltonian (equation 4 in the main text).

$\hat{H}=-(J_{dip}+J_{ex})\hat{\tilde{S}_{1,z}}\cdot\hat{\tilde{S}_{2,z}}$ (eqn. S3)

The dipolar part is considered exactly (*J*_dip_ = –0.006 cm^–1^ for **1_Dy_** and +0.020 cm^–1^ for **3_Dy_**), while the exchange part was obtained from the best fitting of the susceptibility (*J*_ex_ = –0.028 cm^–1^ for **1_Dy_** and –0.32 cm^–1^ for **3_Dy_**). The best simulation of magnetic data results in non-magnetic ground states (Table S13), indicating antiferromagnetic interactions for both complexes, with *J*_tot_ = –0.034 cm^–1^ for **1_Dy_** and *J*_tot_ = –0.30 cm^–1^ for **3_Dy_**.

**Table S13.** Energies, tunnelling gaps and *g*_Z_ values of four low-lying exchange doublets for **1_Dy_** and **3_Dy_**.

| **Complex** | **Energy / cm^–1^** | **Δ_tun_** | ***g*_z_** |
| --- | --- | --- | --- |
| **1_Dy_** | 0.000000  0.000000 | 2.3×10^-8^ | 0.460 |
|  | 0.412114  0.412114 | 4.1×10^-8^ | 39.39 |
|  | 199.0545  199.0545 | 2.9×10^-7^ | 3.980 |
|  | 199.4885  199.4885 | 7.5×10^-7^ | 36.36 |
| **3_Dy_** | 0.000000  0.185192 | 0.185 | 0.044 |
|  | 1.852101  2.184207 | 0.332 | 27.28 |
|  | 8.869096  9.581371 | 0.712 | 0.205 |
|  | 10.13554  10.26929 | 0.133 | 0.156 |


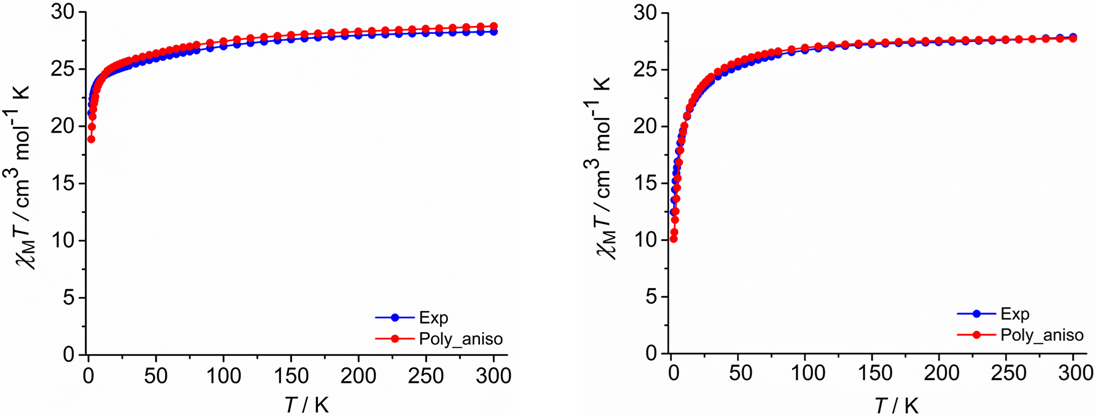


**Figure S41.** Simulation of experimental magnetic susceptibility data using the Lines model for **1_Dy_** (left) and **3_Dy_** (right).

**Table S14.** SINGLE_ANISO computed crystal-field parameters $B_{k}^{q}$ for **1_Dy_** and **3_Dy_**.

|  |  | **1_Dy_** | | **3_Dy_** | |
| --- | --- | --- | --- | --- | --- |
| *k* | *q* | Dy1 | Dy2 | Dy1 | Dy2 |
|  | -2 | 0.2147E+00 | -0.31787E+00 | -0.5317E+00 | -0.5337E+00 |
|  | -1 | 0.6853E+00 | 0.7102E+00 | 0.1523E+01 | 0.1533E+01 |
| 2 | 0 | -0.3945E+01 | -0.3988E+01 | -0.1221E+01 | -0.1218E+01 |
|  | 1 | 0.2845E+00 | -0.2986E+00 | 0.4255E+00 | 0.4245E+00 |
|  | 2 | 0.4102E+01 | 0.4231E+01 | 0.2095E+01 | 0.2098E+01 |
|  |  |  |  |  |  |
|  | -4 | 0.1861E-03 | -0.3869E-03 | -0.1596E-01 | -0.1595E-01 |
|  | -3 | -0.1112E-01 | -0.9227E-02 | -0.3302E-02 | -0.3229E-02 |
|  | -2 | 0.1858E-02 | -0.2092E-02 | 0.6556E-02 | 0.6551E-02 |
|  | -1 | 0.2042E-02 | 0.1981E-02 | -0.2578E-01 | -0.2571E-01 |
| 4 | 0 | 0.5100E-04 | -0.3072E-04 | 0.2150E-05 | -0.4635E-04 |
|  | 1 | 0.2795E-02 | -0.3247E-02 | 0.5493E-02 | 0.5559E-02 |
|  | 2 | -0.6005E-02 | -0.5542E-02 | -0.5829E-02 | -0.5975E-02 |
|  | 3 | -0.4877E-02 | 0.4876E-02 | -0.5532E-02 | -0.5571E-02 |
|  | 4 | -0.3874E-02 | -0.3623E-02 | 0.4265E-02 | 0.4236E-02 |
|  | | |  |  |  |
|  | -6 | 0.2918E-04 | -0.3373E-04 | -0.8925E-04 | -0.9029E-04 |
|  | -5 | -0.6715E-04 | -0.5509E-04 | 0.8125E-04 | 0.8324E-04 |
|  | -4 | 0.2204E-04 | -0.3154E-04 | 0.6368E-04 | 0.6277E-04 |
|  | -3 | 0.3143E-03 | 0.3023E-03 | 0.1407E-03 | 0.1420E-03 |
|  | -2 | 0.4446E-04 | -0.4218E-04 | 0.7724E-06 | 0.3566E-05 |
|  | -1 | -0.9107E-04 | -0.9377E-04 | 0.1489E-03 | 0.1484E-03 |
| 6 | 0 | -0.1876E-04 | -0.1826E-04 | 0.6163E-05 | 0.6418E-05 |
|  | 1 | -0.6799E-04 | 0.7497E-04 | -0.2844E-03 | -0.2844E-03 |
|  | 2 | 0.1822E-03 | 0.1818E-03 | -0.2310E-04 | -0.2078E-04 |
|  | 3 | -0.5231E-04 | 0.4872E-04 | -0.4934E-04 | -0.4793E-04 |
|  | 4 | -0.5693E-04 | -0.5554E-04 | -0.3833E-04 | -0.3719E-04 |
|  | 5 | 0.4136E-04 | -0.4826E-04 | 0.6226E-03 | 0.6233E-03 |
|  | 6 | -0.8934E-04 | -0.9091E-04 | -0.7382E-04 | -0.7392E-04 |

**Table S15.** Calculated energy (cm^–1^) of the low-lying spin-orbit states for **1_Dy_** and **3_Dy_**.

| **1_Dy_** | | **3_Dy_** | |
| --- | --- | --- | --- |
| **Dy1** | **Dy2** | **Dy1** | **Dy2** |
| 0.000  0.000  199.0658  199.0658  324.3940  324.3940  403.1616  403.1616  459.4381  459.4381  543.5965  543.5965  694.2534  694.2534  908.9528  908.9528 | 0.000  0.000  200.2774  200.2774  328.2422  328.2422  407.0839  407.0839  465.5924  465.5924  553.3522  553.3522  704.9530  704.9530  921.8398  921.8398 | 0.000  0.000  9.3282  9.3282  56.9914  56.9914  100.0384  100.0384  152.9208  152.9208  199.7521  199.7521  239.9340  239.9340  392.3203  392.3203 | 0.000  0.000  9.3483  9.3483  56.9839  56.9839  100.0674  100.0674  152.9505  152.9505  199.7964  199.7964  239.9785  239.9785  392.3609  392.3609 |

**Table S16.** Computed energy of the KDs, g-tensors and wavefunction compositions for Dy1 in **1_Dy_**.

| **KD** | ***E* / (cm^–1^)** | ***g_x_*** | ***g_y_*** | ***g_z_*** | **Wavefunction composition** |
| --- | --- | --- | --- | --- | --- |
| 1 | 0.000 | 0.0018 | 0.0026 | 19.70 | 96.6%\|±15/2>+3.0%\|±11/2 |
| 2 | 199.066 | 0.0407 | 0.0585 | 16.77 | 88.8%\|±13/2>+2.5%\|±11/2>+7.6%\|±9/2>+0.8%\|±5/2> |
| 3 | 324.394 | 0.5095 | 0.7400 | 13.64 | 2.2%\|±15/2>+4.4%\|±13/2>+65.9%\|±11/2>+6.1%\|±9/2>+14.3%\|±7/2>+1.7%\|±5/2>+3.9%\|±3/2>+1.4%\|±1/2> |
| 4 | 403.162 | 3.999 | 5.161 | 8.811 | 0.80%\|±15/2>+3.2%\|±13/2>+14.7%\|±11/2>+33.30%\|±9/2>+3.6%\|±7/2>+21.60%\|±5/2>+7.9%\|±3/2>+14.7%\|±1/2> |
| 5 | 459.438 | 2.780 | 3.924 | 10.10 | 0.2%\|±15/2>+2.80%\|±13/2>+8.70%\|±11/2>+29.90%\|±9/2>+22.90%\|±7/2>+2.80%\|±5/2>+22.50%\|±3/2>+10.2%\|±1/2> |
| 6 | 543.597 | 0.3014 | 0.4210 | 14.167 | 0.10%\|±15/2>+0.60%\|±13/2>+4.60%\|±11/2>+18.20%\|±9/2>+37.50%\|±7/2>+23.2%\|±5/2>+0.40%\|±3/2>+15.30%\|±1/2> |
| 7 | 694.253 | 0.0346 | 0.0459 | 16.72 | 0.50%\|±11/2>+4.50%\|±9/2>+18.9%\|±7/2>+38.00%\|±5/2>+32.60%\|±3/2>+5.7%\|±1/2> |
| 8 | 908.953 | 0.0029 | 0.0049 | 19.47 | 0.3%\|±9/2>+2.5%\|±7/2>+12.00%\|±5/2>+32.60%\|±3/2>+52.7%\|±1/2> |


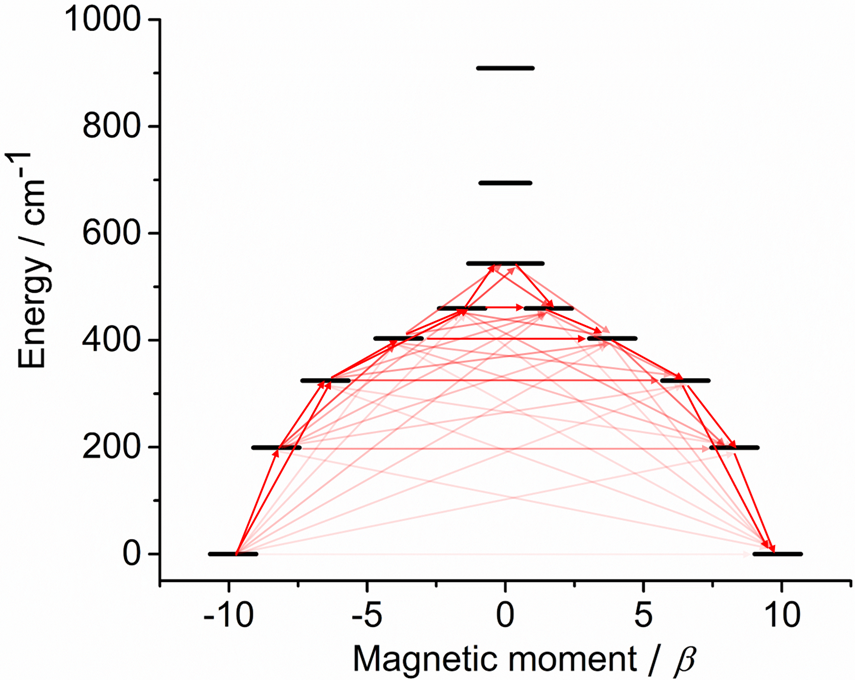


**Figure S42.** Calculated relaxation barrier for Dy1 in **1_Dy_**. Stronger red arrows indicate larger absolute value of the transition magnetic moment matrix elements between the respective states. Transitions involving higher-energy states not involved in the relaxation mechanism are omitted for clarity.

**Table S17.** Computed energy of the KDs, g-tensors and wavefunction compositions for Dy2 in **1_Dy_**.

| **KD** | ***E* / (cm^–1^)** | ***g_x_*** | ***g_y_*** | ***g_z_*** | **Wavefunction composition** |
| --- | --- | --- | --- | --- | --- |
| 1 | 0.000 | 0.0020 | 0.0029 | 19.69 | 96.4%\|±15/2>+3.2%\|±11/2> |
| 2 | 200.277 | 0.0478 | 0.0689 | 16.74 | 88.3%\|±13/2>+2.6%\|±11/2>+8.0%\|±9/2>+0.8%\|±5/2> |
| 3 | 328.242 | 0.6217 | 0.9059 | 13.53 | 2.3%\|±15/2>+4.4%\|±13/2>+65.0%\|±11/2>+6.0%\|±9/2>+15.20%\|±7/2>+1.7%\|±5/2>+4.10%\|±3/2>+1.5%\|±1/2> |
| 4 | 407.084 | 4.367 | 5.546 | 8.55 | 0.80%\|±15/2>+3.40%\|±13/2>+14.0%\|±11/2>+31.40%\|±9/2>+3.80%\|±7/2>+22.70%\|±5/2>+8.40%\|±3/2>+15.50%\|±1/2> |
| 5 | 465.592 | 2.351 | 3.337 | 10.39 | 0.2%\|±15/2>+3.10%\|±13/2>+9.60%\|±11/2>+30.40%\|±9/2>+21.50%\|±7/2>+2.80%\|±5/2>+22.50%\|±3/2>+9.90%\|±1/2> |
| 6 | 553.352 | 0.2571 | 0.3564 | 14.24 | 0.10%\|±15/2>+0.70%\|±13/2>+5.10%\|±11/2>+19.10%\|±9/2>+37.50%\|±7/2>+22.0%\|±5/2>+0.40%\|±3/2>+15.20%\|±1/2> |
| 7 | 704.953 | 0.0326 | 0.0429 | 16.77 | 0.50%\|±11/2>+4.80%\|±9/2>+19.30%\|±7/2>+37.80%\|±5/2>+32.0%\|±3/2>+5.60%\|±1/2> |
| 8 | 921.840 | 0.0029 | 0.0048 | 19.50 | 0.20%\|±9/2>+2.5%\|±7/2>+12.30%\|±5/2>+32.60%\|±3/2>+52.20%\|±1/2> |


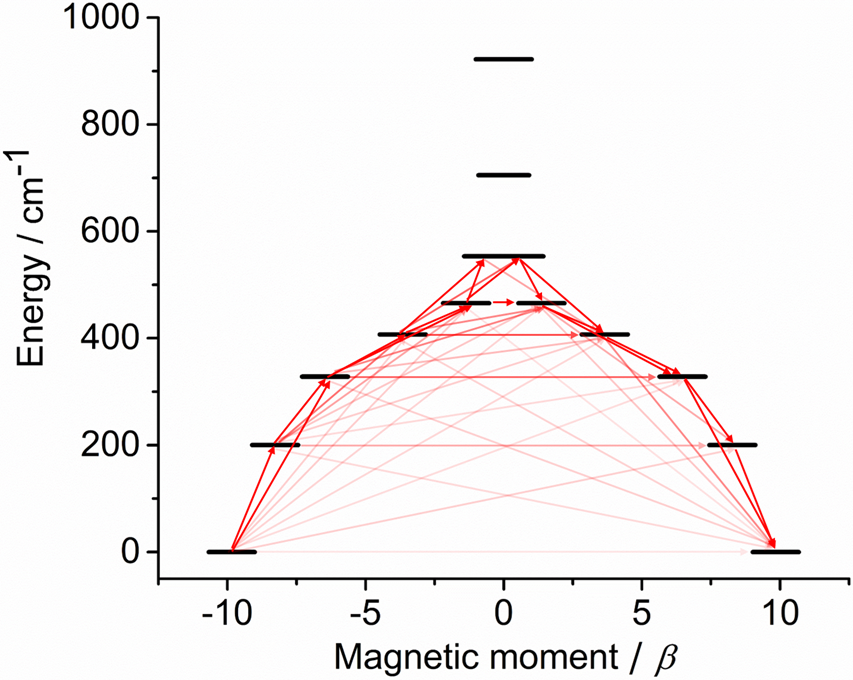


**Figure S43.** Calculated relaxation barrier for Dy2 in **1_Dy_**. Stronger red arrows indicate larger absolute value of the transition magnetic moment matrix elements between the respective states. Transitions involving higher-energy states not involved in the relaxation mechanism are omitted for clarity.

**Table S18.** Computed energy of the KDs, g-tensors and wavefunction compositions for Dy1 in **3_Dy_**.

| **KD** | ***E* / (cm^–1^)** | ***g_x_*** | ***g_y_*** | ***g_z_*** | **Wavefunction composition** |
| --- | --- | --- | --- | --- | --- |
| 1 | 0.000 | 0.0365 | 4.693 | 14.51 | 44.30%\|±15/2>+20.2%\|±13/2>+9.60%\|±11/2>+3.20%\|±9/2>+7.10%\|±7/2>+7.0%\|±5/2>+4.80%\|±3/2>+3.80%\|±1/2> |
| 2 | 9.328 | 0.0040 | 4.2503 | 14.48 | 25.20%\|±15/2>+9.30%\|±13/2>+3.50%\|±11/2>+12.80%\|±9/2>+15.30%\|±7/2>+15.80%\|±5/2>+11.80%\|±3/2>+6.10%\|±1/2> |
| 3 | 56.991 | 0.2721 | 0.7984 | 16.88 | 28.40%\|±15/2>+47.60%\|±13/2>+13.60%\|±11/2>+5.30%\|±9/2>+0.40%\|±7/2>+2.40%\|±5/2>+0.90%\|±3/2>+1.30%\|±1/2> |
| 4 | 100.038 | 2.4178 | 3.4409 | 13.68 | 1.50%\|±15/2>+14.40%\|±13/2>+43.10%\|±11/2>+11.50%\|±9/2>+2.90%\|±7/2>+2.60%\|±5/2>+12.20%\|±3/2>+12.0%\|±1/2> |
| 5 | 152.921 | 8.8054 | 5.750 | 0.090 | 0.30%\|±15/2>+6.50%\|±13/2>+16.60%\|±11/2>+28.0%\|±9/2>+11.30%\|±7/2>+2.90%\|±5/2>+5.90%\|±3/2>+28.50%\|±1/2> |
| 6 | 199.752 | 1.4356 | 3.835 | 12.60 | 0.10%\|±15/2>+0.90%\|±13/2>+4.50%\|±11/2>+14.90%\|±9/2>+16.90%\|±7/2>+20.40%\|±5/2>+28.90%\|±3/2>+13.50%\|±1/2> |
| 7 | 239.934 | 1.404 | 4.309 | 14.22 | 0.10%\|±15/2>+0.90%\|±13/2>+4.50%\|±11/2>+14.90%\|±9/2>+16.90%\|±7/2>+20.40%\|±5/2>+28.90%\|±3/2>+13.50%\|±1/2> |
| 8 | 392.320 | 0.0612 | 0.1243 | 19.49 | 0.10%\|±15/2>+0.90%\|±13/2>+8.40%\|±11/2>+21.20%\|±9/2>+35.10%\|±7/2>+24.80%\|±5/2>+4.20%\|±3/2>+5.40%\|±1/2> |


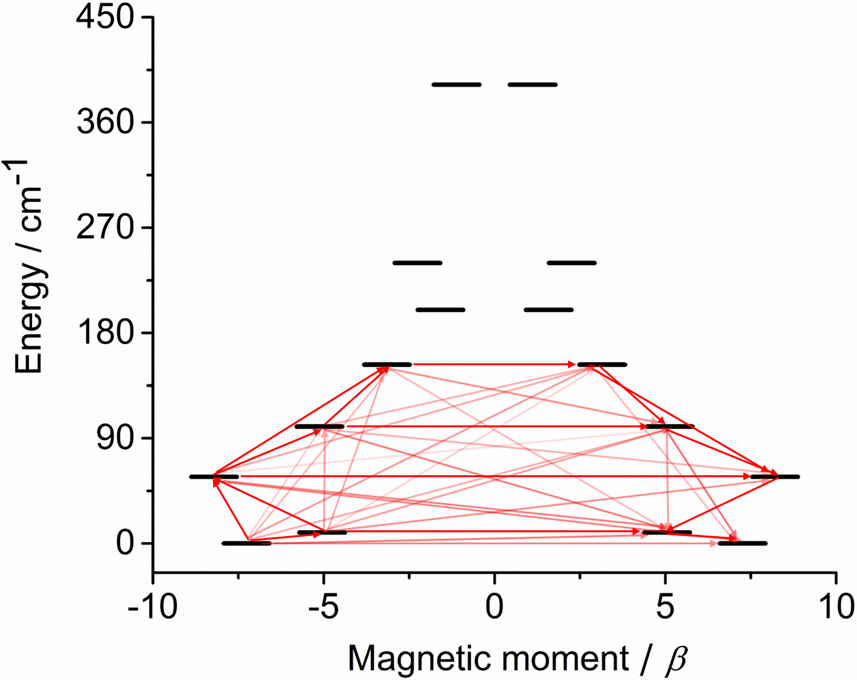


**Figure S44.** Calculated relaxation barrier for Dy1 in **3_Dy_**. Stronger red arrows indicate larger absolute value of the transition magnetic moment matrix elements between the respective states. Transitions involving higher-energy states not involved in the relaxation mechanism are omitted for clarity.

**Table S19.** Computed energy of the KDs, g-tensors and wavefunction compositions for Dy2 in **3_Dy_**.

| **KD** | ***E* / (cm^–1^)** | ***g_x_*** | ***g_y_*** | ***g_z_*** | **Wavefunction composition** |
| --- | --- | --- | --- | --- | --- |
| 1 | 0.000 | 0.0366 | 4.665 | 14.54 | 44.80%\|±15/2>+20.0%\|±13/2>+9.50%\|±11/2>+3.20%\|±9/2>+6.90%\|±7/2>+7.0%\|±5/2>+4.80%\|±3/2>+3.90%\|±1/2> |
| 2 | 9.348 | 0.004 | 4.226 | 14.51 | 25.20%\|±15/2>+9.0%\|±13/2>+3.40%\|±11/2>+12.60%\|±9/2>+15.30%\|±7/2>+15.90%\|±5/2>+12.10%\|±3/2>+6.30%\|±1/2> |
| 3 | 56.984 | 0.270 | 0.797 | 16.88 | 27.90%\|±15/2>+48.60%\|±13/2>+13.20%\|±11/2>+5.10%\|±9/2>+0.40%\|±7/2>+2.40%\|±5/2>+0.90%\|±3/2>+1.40%\|±1/2> |
| 4 | 100.067 | 2.419 | 3.440 | 13.68 | 1.50%\|±15/2>+14.00%\|±13/2>+43.10%\|±11/2>+11.90%\|±9/2>+2.80%\|±7/2>+2.40%\|±5/2>+12.30%\|±3/2>+12.10%\|±1/2> |
| 5 | 152.951 | 8.802 | 5.751 | 0.088 | 0.30%\|±15/2>+6.40%\|±13/2>+17.10%\|±11/2>+27.50%\|±9/2>+11.40%\|±7/2>+3.10%\|±5/2>+5.70%\|±3/2>+28.50%\|±1/2> |
| 6 | 199.796 | 1.441 | 3.835 | 12.60 | 0.20%\|±15/2>+0.90%\|±13/2>+4.50%\|±11/2>+15.20%\|±9/2>+16.80%\|±7/2>+20.40%\|±5/2>+29.0%\|±3/2>+13.00%\|±1/2> |
| 7 | 239.978 | 1.403 | 4.301 | 14.23 | 0.10%\|±15/2>+0.90%\|±13/2>+8.40%\|±11/2>+21.40%\|±9/2>+35.10%\|±7/2>+24.50%\|±5/2>+4.10%\|±3/2>+5.40%\|±1/2> |
| 8 | 392.361 | 0.061 | 0.124 | 19.49 | 0.10%\|±13/2>+0.80%\|±11/2>+3.10%\|±9/2>+11.20%\|±7/2>+24.20%\|±5/2>+31.10%\|±3/2>+29.40%\|±1/2> |


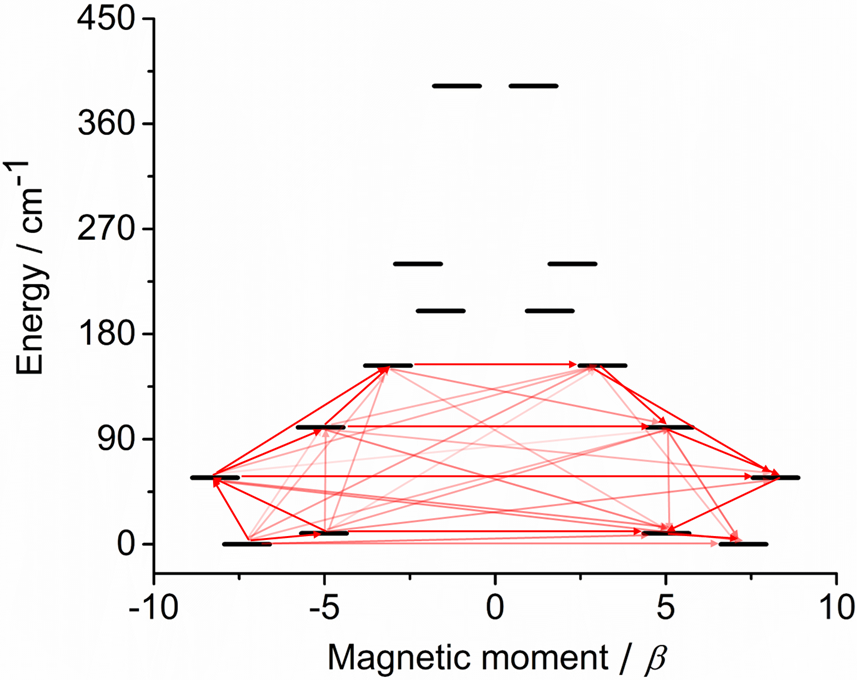


**Figure S45.** Calculated relaxation barrier for Dy2 in **3_Dy_**. Stronger red arrows indicate larger absolute value of the transition magnetic moment matrix elements between the respective states. Transitions involving higher-energy states not involved in the relaxation mechanism are omitted for clarity.

**Table S20.** Magnitudes of transition magnetic moment matrix elements (in Bohr magneton) for Dy1 in **1_Dy_**.

| **Climbing Transition** | | | **Crossing Transition** | | |
| --- | --- | --- | --- | --- | --- |
| **Initial KD** | **Final KD** | **Magnitude** | **Initial KD** | **Final KD** | **Magnitude** |
| 1 | 2 | 1.773 | 1 | 1 | 0.0007537 |
| 1 | 3 | 0.4649 | 1 | 2 | 0.00375 |
| 1 | 4 | 0.1408 | 1 | 3 | 0.008407 |
| 1 | 5 | 0.08024 | 1 | 4 | 0.03009 |
| 1 | 6 | 0.06942 | 1 | 5 | 0.05906 |
| 1 | 7 | 0.3730 | 1 | 6 | 0.05209 |
| 1 | 8 | 0.00463 | 1 | 7 | 0.02150 |
| 2 | 3 | 2.450 | 1 | 8 | 0.007718 |
| 2 | 4 | 0.6496 | 2 | 2 | 0.0166 |
| 2 | 5 | 0.2289 | 2 | 3 | 0.03882 |
| 2 | 6 | 0.1269 | 2 | 4 | 0.1366 |
| 2 | 7 | 0.09516 | 2 | 5 | 0.1038 |
| 2 | 8 | 0.03144 | 2 | 6 | 0.07622 |
| 3 | 4 | 2.946 | 2 | 7 | 0.08557 |
| 3 | 5 | 0.7386 | 2 | 8 | 0.02987 |
| 3 | 6 | 0.2711 | 3 | 3 | 0.2141 |
| 3 | 7 | 0.1719 | 3 | 4 | 0.3056 |
| 3 | 8 | 0.06992 | 3 | 5 | 0.7339 |
| 4 | 5 | 2.952 | 3 | 6 | 0.3129 |
| 4 | 6 | 0.5673 | 3 | 7 | 0.1527 |
| 4 | 7 | 0.2228 | 3 | 8 | 0.06541 |
| 4 | 8 | 0.09133 | 4 | 4 | 1.646 |
| 5 | 6 | 2.394 | 4 | 5 | 1.405 |
| 5 | 7 | 0.3808 | 4 | 6 | 0.7272 |
| 5 | 8 | 0.1212 | 4 | 7 | 0.2588 |
| 6 | 7 | 2.285 | 4 | 8 | 0.08907 |
| 6 | 8 | 0.5542 | 5 | 5 | 2.180 |
| 7 | 8 | 0.2464 | 5 | 6 | 1.458 |
|  |  |  | 5 | 7 | 0.2836 |
|  |  |  | 5 | 8 | 0.1681 |
|  |  |  | 6 | 6 | 0.8508 |
|  |  |  | 6 | 7 | 0.3783 |
|  |  |  | 6 | 8 | 0.3025 |
|  |  |  | 7 | 7 | 0.7592 |
|  |  |  | 7 | 8 | 1.778 |
|  |  |  | 8 | 8 | 0.03418 |

**Table S21.** Magnitudes of transition magnetic moment matrix elements (in Bohr magneton) for Dy2 in **1_Dy_**.

| **Climbing Transition** | | | **Crossing Transition** | | |
| --- | --- | --- | --- | --- | --- |
| **Initial KD** | **Final KD** | **Magnitude** | **Initial KD** | **Final KD** | **Magnitude** |
| 1 | 2 | 1.777 | 1 | 1 | 0.0008333 |
| 1 | 3 | 0.4686 | 1 | 2 | 0.004196 |
| 1 | 4 | 0.1359 | 1 | 3 | 0.01041 |
| 1 | 5 | 0.8439 | 1 | 4 | 0.03663 |
| 1 | 6 | 0.07413 | 1 | 5 | 0.0596 |
| 1 | 7 | 0.03710 | 1 | 6 | 0.0464 |
| 1 | 8 | 0.00459 | 1 | 7 | 0.0217 |
| 2 | 3 | 2.451 | 1 | 8 | 0.007659 |
| 2 | 4 | 0.6404 | 2 | 2 | 0.01961 |
| 2 | 5 | 0.2509 | 2 | 3 | 0.04685 |
| 2 | 6 | 0.1198 | 2 | 4 | 0.1701 |
| 2 | 7 | 0.09606 | 2 | 5 | 0.1202 |
| 2 | 8 | 0.03051 | 2 | 6 | 0.07162 |
| 3 | 4 | 2.926 | 2 | 7 | 0.07775 |
| 3 | 5 | 0.7714 | 2 | 8 | 0.02943 |
| 3 | 6 | 0.2639 | 3 | 3 | 0.2614 |
| 3 | 7 | 0.1672 | 3 | 4 | 0.3546 |
| 3 | 8 | 0.06649 | 3 | 5 | 0.7972 |
| 4 | 5 | 2.881 | 3 | 6 | 0.3203 |
| 4 | 6 | 0.5560 | 3 | 7 | 0.1473 |
| 4 | 7 | 0.2065 | 3 | 8 | 0.06438 |
| 4 | 8 | 0.08716 | 4 | 4 | 1.809 |
| 5 | 6 | 2.437 | 4 | 5 | 1.516 |
| 5 | 7 | 0.4182 | 4 | 6 | 0.6581 |
| 5 | 8 | 0.1146 | 4 | 7 | 0.2456 |
| 6 | 7 | 2.311 | 4 | 8 | 0.08891 |
| 6 | 8 | 0.04138 | 5 | 5 | 2.092 |
| 7 | 8 | 0.1716 | 5 | 6 | 1.388 |
|  |  |  | 5 | 7 | 0.2700 |
|  |  |  | 5 | 8 | 0.1778 |
|  |  |  | 6 | 6 | 0.6270 |
|  |  |  | 6 | 7 | 0.2865 |
|  |  |  | 6 | 8 | 0.3121 |
|  |  |  | 7 | 7 | 0.5276 |
|  |  |  | 7 | 8 | 1.789 |
|  |  |  | 8 | 8 | 0.02827 |

**Table S22.** Magnitudes of transition magnetic moment matrix elements (in Bohr magneton) for Dy1 in **3_Dy_**.

| **Climbing Transition** | | | **Crossing Transition** | | |
| --- | --- | --- | --- | --- | --- |
| **Initial KD** | **Final KD** | **Magnitude** | **Initial KD** | **Final KD** | **Magnitude** |
| 1 | 2 | 2.449 | 1 | 1 | 0.7882 |
| 1 | 3 | 1.577 | 1 | 2 | 1.596 |
| 1 | 4 | 1.424 | 1 | 3 | 0.4073 |
| 1 | 5 | 0.6579 | 1 | 4 | 0.3455 |
| 1 | 6 | 0.2166 | 1 | 5 | 0.3646 |
| 1 | 7 | 0.1233 | 1 | 6 | 0.1437 |
| 1 | 8 | 0.1192 | 1 | 7 | 0.09766 |
| 2 | 3 | 1.452 | 1 | 8 | 0.06105 |
| 2 | 4 | 1.599 | 2 | 2 | 1.144 |
| 2 | 5 | 0.8056 | 2 | 3 | 0.6426 |
| 2 | 6 | 0.1808 | 2 | 4 | 0.5049 |
| 2 | 7 | 0.2718 | 2 | 5 | 0.3508 |
| 2 | 8 | 0.08135 | 2 | 6 | 0.3736 |
| 3 | 4 | 1.6098 | 2 | 7 | 0.1111 |
| 3 | 5 | 1.433 | 2 | 8 | 0.1617 |
| 3 | 6 | 0.6512 | 3 | 3 | 0.1860 |
| 3 | 7 | 0.2685 | 3 | 4 | 0.9754 |
| 3 | 8 | 0.2619 | 3 | 5 | 0.2765 |
| 4 | 5 | 2.587 | 3 | 6 | 0.3200 |
| 4 | 6 | 0.6692 | 3 | 7 | 0.2443 |
| 4 | 7 | 0.5372 | 3 | 8 | 0.08624 |
| 4 | 8 | 0.240 | 4 | 4 | 1.113 |
| 5 | 6 | 2.507 | 4 | 5 | 1.386 |
| 5 | 7 | 1.454 | 4 | 6 | 0.6171 |
| 5 | 8 | 0.5109 | 4 | 7 | 0.3825 |
| 6 | 7 | 1.958 | 4 | 8 | 0.3509 |
| 6 | 8 | 0.4816 | 5 | 5 | 1.542 |
| 7 | 8 | 0.7083 | 5 | 6 | 1.622 |
|  |  |  | 5 | 7 | 1.250 |
|  |  |  | 5 | 8 | 0.5069 |
|  |  |  | 6 | 6 | 2.626 |
|  |  |  | 6 | 7 | 2.085 |
|  |  |  | 6 | 8 | 0.6745 |
|  |  |  | 7 | 7 | 1.816 |
|  |  |  | 7 | 8 | 1.641 |
|  |  |  | 8 | 8 | 0.1499 |

**Table S23.** Magnitudes of transition magnetic moment matrix elements (in Bohr magneton) for Dy2 in **3_Dy_**.

| **Climbing Transition** | | | **Crossing Transition** | | |
| --- | --- | --- | --- | --- | --- |
| **Initial KD** | **Final KD** | **Magnitude** | **Initial KD** | **Final KD** | **Magnitude** |
| 1 | 2 | 2.445 | 1 | 1 | 0.7836 |
| 1 | 3 | 1.577 | 1 | 2 | 1.595 |
| 1 | 4 | 1.422 | 1 | 3 | 0.4062 |
| 1 | 5 | 0.6574 | 1 | 4 | 0.3450 |
| 1 | 6 | 0.2161 | 1 | 5 | 0.3640 |
| 1 | 7 | 0.1237 | 1 | 6 | 0.1437 |
| 1 | 8 | 0.1194 | 1 | 7 | 0.09764 |
| 2 | 3 | 1.450 | 1 | 8 | 0.06067 |
| 2 | 4 | 1.600 | 2 | 2 | 1.146 |
| 2 | 5 | 0.8063 | 2 | 3 | 0.6434 |
| 2 | 6 | 0.1795 | 2 | 4 | 0.5043 |
| 2 | 7 | 0.2718 | 2 | 5 | 0.3511 |
| 2 | 8 | 0.08122 | 2 | 6 | 0.3744 |
| 3 | 4 | 1.609 | 2 | 7 | 0.1108 |
| 3 | 5 | 1.433 | 2 | 8 | 0.1619 |
| 3 | 6 | 0.6499 | 3 | 3 | 0.1857 |
| 3 | 7 | 0.2685 | 3 | 4 | 0.9747 |
| 3 | 8 | 0.2619 | 3 | 5 | 0.2768 |
| 4 | 5 | 2.588 | 3 | 6 | 0.3210 |
| 4 | 6 | 0.6690 | 3 | 7 | 0.2445 |
| 4 | 7 | 0.5367 | 3 | 8 | 0.08624 |
| 4 | 8 | 0.2401 | 4 | 4 | 1.115 |
| 5 | 6 | 2.500 | 4 | 5 | 1.387 |
| 5 | 7 | 1.454 | 4 | 6 | 0.6176 |
| 5 | 8 | 0.5110 | 4 | 7 | 0.3826 |
| 6 | 7 | 1.952 | 4 | 8 | 0.3510 |
| 6 | 8 | 0.4799 | 5 | 5 | 1.544 |
| 7 | 8 | 0.7092 | 5 | 6 | 1.634 |
|  |  |  | 5 | 7 | 1.249 |
|  |  |  | 5 | 8 | 0.5073 |
|  |  |  | 6 | 6 | 2.613 |
|  |  |  | 6 | 7 | 2.089 |
|  |  |  | 6 | 8 | 0.6752 |
|  |  |  | 7 | 7 | 1.818 |
|  |  |  | 7 | 8 | 1.641 |
|  |  |  | 8 | 8 | 0.1490 |

**References:**

1. C. G. T. Price, A. Mondal, J. P. Durrant, J. Tang, R. A. Layfield, *Inorg. Chem.* **2023**, *62*, 9924-9933.
2. O. V. Dolomanov, L. J. Bourhis, R. Gildea, J. A. K. Howard, H. Puschmann. *J. Appl. Cryst.* **2009,** *42*, 339-341.
3. L. J. Bourhis, O. V. Dolomanov, R. J. Gildea, J. A. K. Howard, H. Puschmann. *Acta Cryst. Section A* **2015,** *71*, 59-75.
4. G. Sheldrick. *Acta Cryst. Section C* **2015,** *71*, 3-8.
5. F. Neese, F. Wennmohs, U. Becker, C. Riplinger, *J. Chem. Phys.* **2020**, *152*, 224108.
6. J. P. Perdew, K. Burke, M. Ernzerhof, *Phys. Rev. Lett.* **1997**, *78*, 1396-1396.
7. J. P. Perdew, K. Burke and M. Ernzerhof, *Phys. Rev. Lett.* **1996**, *77*, 3865-3868.
8. J. Tao, J. P. Perdew, V. N. Staroverov, G. E. Scuseria, *Phys. Rev. Lett.* **2003**, *91*, 146401.
9. D. Aravena, F. Neese and D. A. Pantazis, *J. Chem. Theory Comput.* **2016**, *12*, 1148-1156.
10. J. Chmela and M. E. Harding, *Mol. Phys.* **2018**, *116*, 1523-1538.
11. J. D. Rolfes, F. Neese and D. A. Pantazis, *J. Comput. Chem.* **2020**, *41*, 1842–1849.
12. R. Izsák and F. Neese, *J. Chem. Phys.* **2011**, *135*, 144105.
13. F. Neese, F. Wennmohs, A. Hansen and U. Becker, *Chem. Phys.* **2009**, *356*, 98–109.
14. V. Barone, M. Cossi, *J. Phys. Chem. A* **1998**, *102*, 1995–2001.
15. M. Cossi, N. Rega, G. Scalmani, V. Barone, J. Comput. Chem. **2003**, *24*, 669–681.
16. M. J. Frisch, G. W. Trucks, et al. (2016) Gaussian 16.
17. G. A. Bain, J. F. Berry, *J. Chem. Educ*. **2008**, *85*, 532-536.
18. A. Schäfer, H. Horn, R. Ahlrichs, *J. Chem. Phys.* **1992**, *97*, 2571-2577.
19. F. Weigend, R. Ahlrichs, *Phys. Chem. Chem. Phys.* **2005**, *7*, 3297-3305.
20. D. Ganyushin and F. Neese, *J. Chem. Phys.* **2006**, *125*, 024103.
21. L. F. Chibotaru and L. Ungur, *J. Chem. Phys.* **2012**, *137*, 064112.
